# Supplementary material for: Design, Synthesis and Antifungal Evaluation of Novel Pyrylium Salt In Vitro and In Vivo
Source: Molecules. 2022 Jul 12;27(14):4450. doi: 10.3390/molecules27144450 (PMC9315806; doi:10.3390/molecules27144450)

Supplementary Materials

Design, Synthesis and Antifungal Evaluation of Novel Pyrylium Salt In Vitro and In Vivo

Yue Zhang [1,†], Qiuhao Li [1,†], Wen Chao [2,†], Yulin Qin [3], Jiayan Chen [1], Yingwen Wang [1], Runhui Liu [1,*], Quanzhen Lv [1,*] and Jinxin Wang [1,*]

[1] School of Pharmacy, Naval Medical University, Shanghai 200433, China; yzhang_moon@163.com (Y.Z.); lqh20191034@163.com (Q.L.); chenjiayan_alice@outlook.com (J.C.); wyw20010625@163.com (Y.W.)
[2] Experimental Teaching Center of Basic Medicine College, Navel Medical University, Shanghai 200433, China; chaowen_2010@163.com
[3] Fudan University Minhang Hospital, Shanghai 201199, China; qinyulin1990@126.com
* Correspondence: lyliurh@126.com (R.L.); lvquanzhen2011@163.com (Q.L.); jxwang2013@126.com (J.W.)
† These authors contributed equally to this work.

CONTENTS:

- HR-MS, ^{1}H NMR, ^{13}C NMR and ^{19}F NMR Spectra of Compounds 2

1. HR-MS, ^{1}H, ^{13}C NMR and ^{19}F NMR Spectra of Compounds

HR-MS Spectra of XY3

Best	ID Source	Name	Formula	Species	m/z	Score	Score (RT)	RT Diff	Diff (ppm)	Score (Lib)	Score (DB)	Score (MFG)
TRUE	MFG		C22 H29 O2	M+	325.2166	98.69			-1.17			98.69

Species	m/z	Score (iso. abund)	Score (mass)	Score (MFG, MS/MS)	Score (MS)	Score (MFG)	Score (iso. spacing)	Height	Ion Formula
M+	325.2166	97.13	99.01		98.69	98.69	99.92	1776356.8	C22 H29 O2

Height (Calc)	Height Sum% (Calc)	Height % (Calc)	m/z (Calc)	Diff (mDa)	Height	Height %	Height Sum %	m/z	Diff (ppm)
1746403.3	78.5	100	325.2162	-0.4	1776356.8	100	79.8	325.2166	-1.11
422705.2	19	24.2	326.2196	-0.5	393755.5	22.2	17.7	326.2201	-1.48
56086.3	2.5	3.2	327.2227	-0.3	55082.6	3.1	2.5	327.223	-1.03

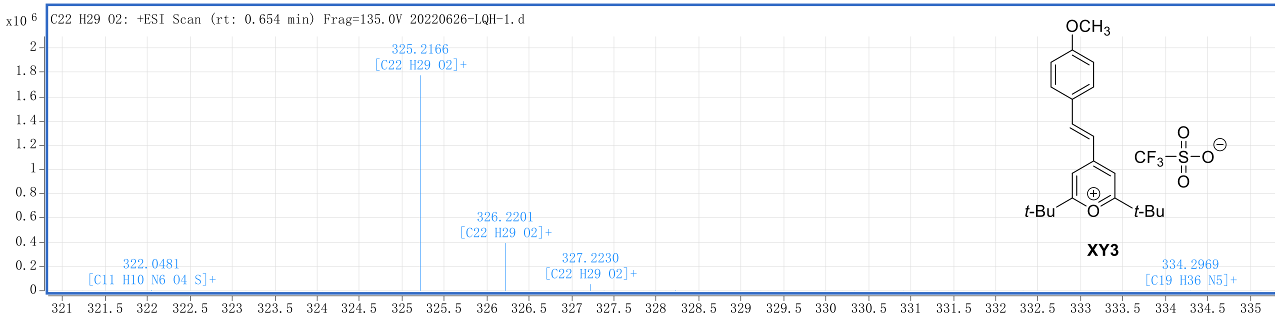

Best	ID Source	Name	Formula	Species	m/z	Score	Score (RT)	RT Diff	Diff (ppm)	Score (Lib)	Score (DB)	Score (MFG)
TRUE	MFG		C F3 O3 S	M-	148.9529	99.22			-2.16			99.22

Species	m/z	Score (iso. abund)	Score (mass)	Score (MFG, MS/MS)	Score (MS)	Score (MFG)	Score (iso. spacing)	Height	Ion Formula
M-	148.9529	99.98	98.72		99.22	99.22	99.29	5554232.5	C F3 O3 S

Height (Calc)	Height Sum% (Calc)	Height % (Calc)	m/z (Calc)	Diff (mDa)	Height	Height %	Height Sum %	m/z	Diff (ppm)
5562054.4	93.4	100	148.9526	-0.4	5554232.5	100	93.3	148.9529	-2.45
110429.4	1.9	2	149.9544	0.5	113172.8	2	1.9	149.9539	3.26
283741.2	4.8	5.1	150.9494	0.2	288819.8	5.2	4.8	150.9492	1.39

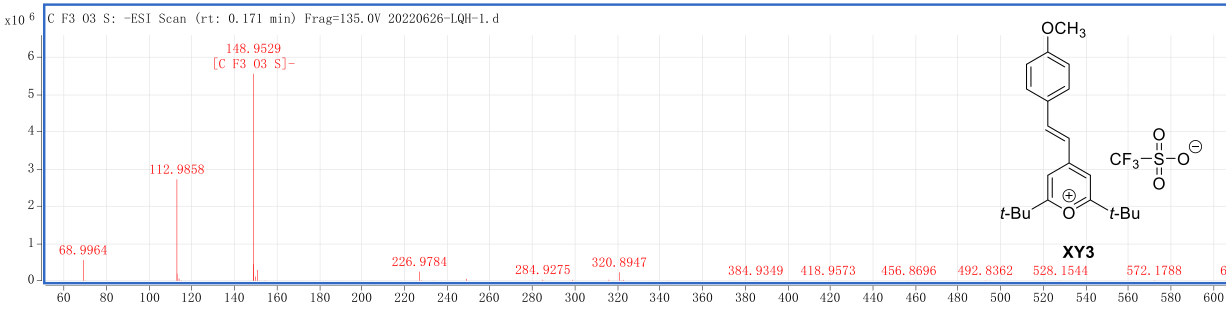

^{1}H NMR Spectrum of XY3 (500 MHz, $CDCl_3$)

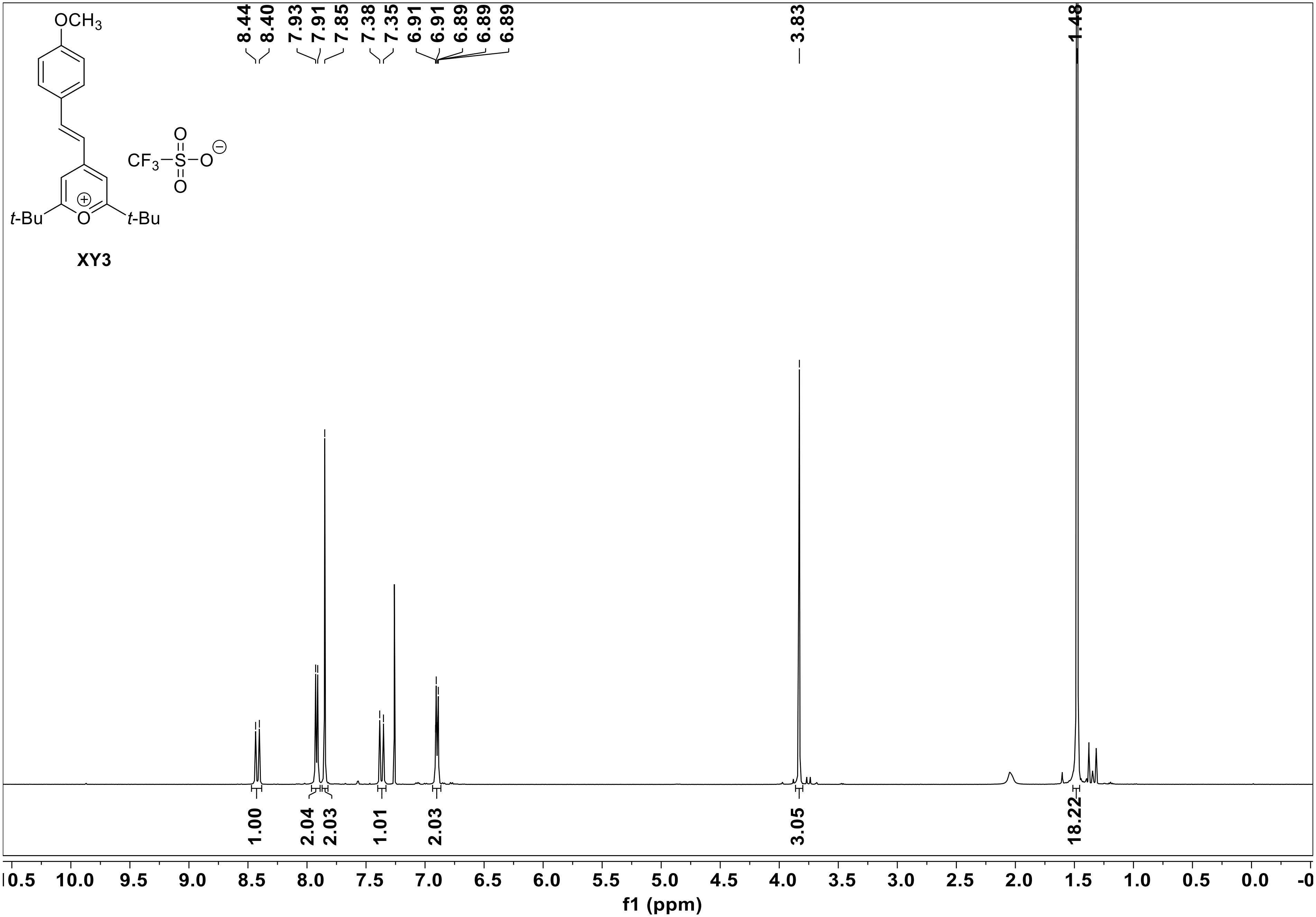

^{13}C NMR Spectrum of XY3 (126 MHz, $CDCl_3$)

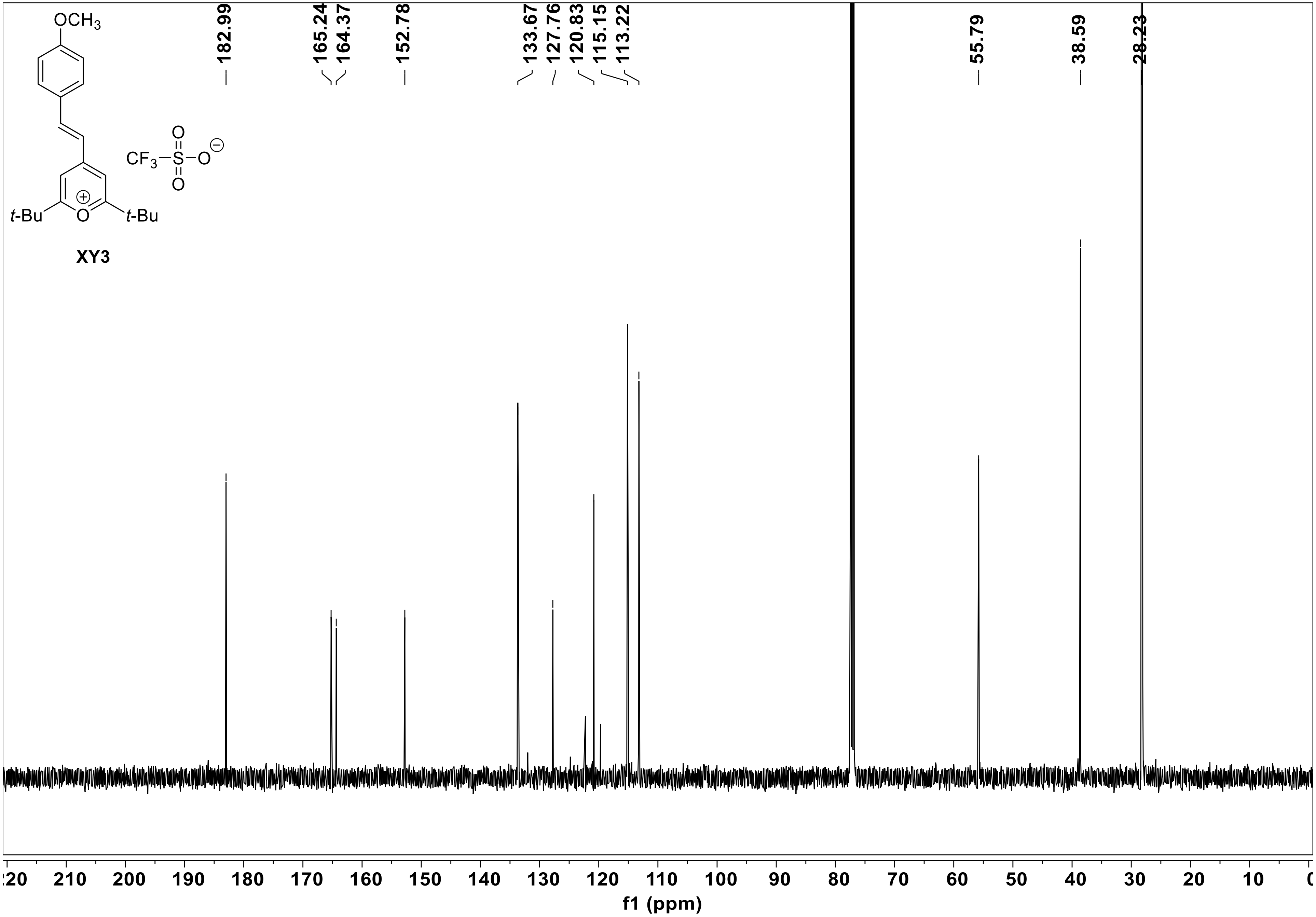

^{19}F NMR Spectrum of XY3 (282 MHz, $CDCl_3$)

XY3

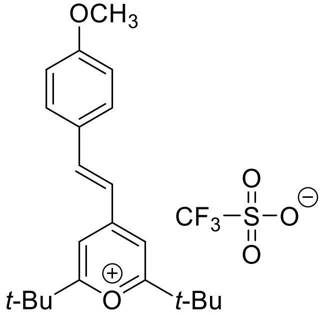

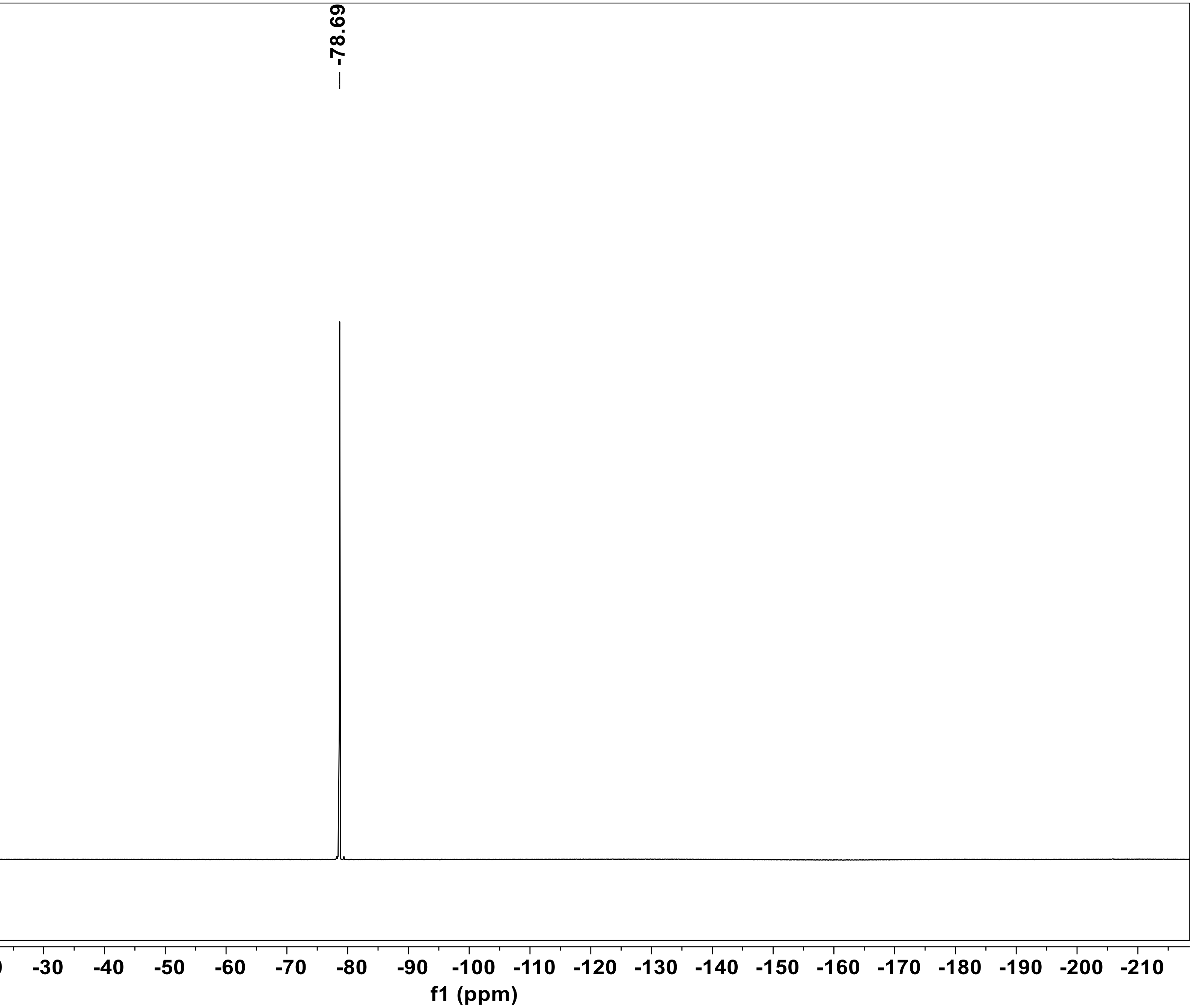

HR-MS Spectra of XY4

Best	ID Source	Name	Formula	Species	m/z	Score	Score (RT)	RT Diff	Diff (ppm)	Score (Lib)	Score (DB)	Score (MFG)
TRUE	MFG		C21 H27 O2	M+	311.2012	91.83			-1.18			91.83

Species	m/z	Score (iso. abund)	Score (mass)	Score (MFG, MS/MS)	Score (MS)	Score (MFG)	Score (iso. spacing)	Height	Ion Formula
M+	311.2012	81.37	99.04		91.83	91.83	89.95	5152438	C21 H27 O2

Height (Calc)	Height Sum% (Calc)	Height % (Calc)	m/z (Calc)	Diff (mDa)	Height	Height %	Height Sum %	m/z	Diff (ppm)
5397520.7	79.3	100	311.2006	-0.7	5152438	100	75.7	311.2012	-2.23
1246813.8	18.3	23.1	312.204	0.7	1474705	28.6	21.7	312.2033	2.23
159557.7	2.3	3	313.207	0.2	176749.2	3.4	2.6	313.2068	0.77

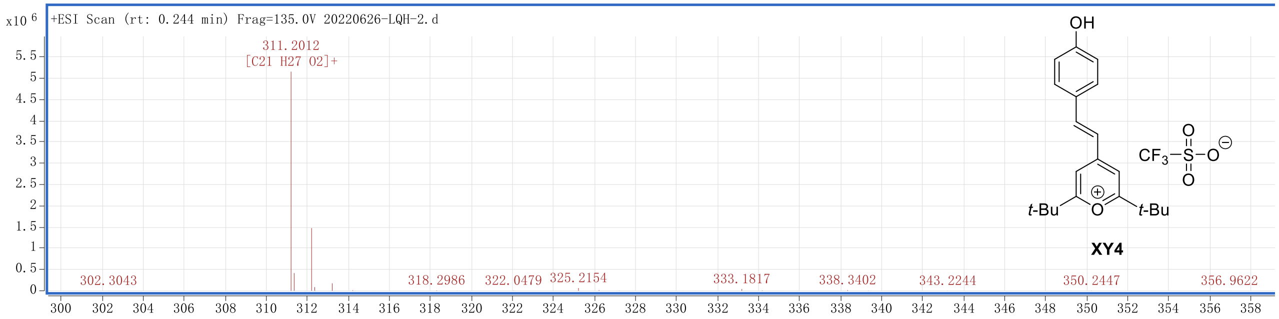

Best	ID Source	Name	Formula	Species	m/z	Score	Score (RT)	RT Diff	Diff (ppm)	Score (Lib)	Score (DB)	Score (MFG)
TRUE	MFG		C F3 O3 S	M-	148.9528	99.38			-1.64			99.38

Species	m/z	Score (iso. abund)	Score (mass)	Score (MFG, MS/MS)	Score (MS)	Score (MFG)	Score (iso. spacing)	Height	Ion Formula
M-	148.9528	99.12	99.26		99.38	99.38	99.9	1967462.6	C F3 O3 S

Height (Calc)	Height Sum% (Calc)	Height % (Calc)	m/z (Calc)	Diff (mDa)	Height	Height %	Height Sum %	m/z	Diff (ppm)
1952559.7	93.4	100	148.9526	-0.3	1967462.6	100	94.1	148.9528	-1.74
38766.3	1.9	2	149.9544	0.2	38592.9	2	1.8	149.9542	1.16
99607.4	4.8	5.1	150.9494	-0.1	84877.8	4.3	4.1	150.9495	-0.6

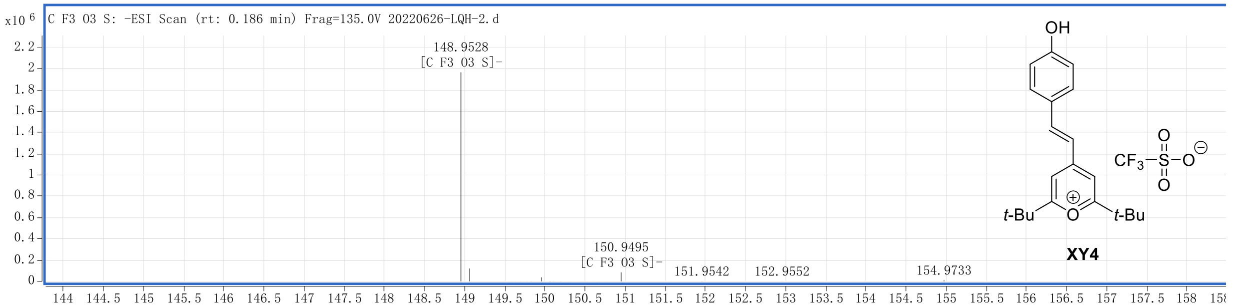

^{1}H NMR Spectrum of XY4 (500 MHz, $CDCl_3$)

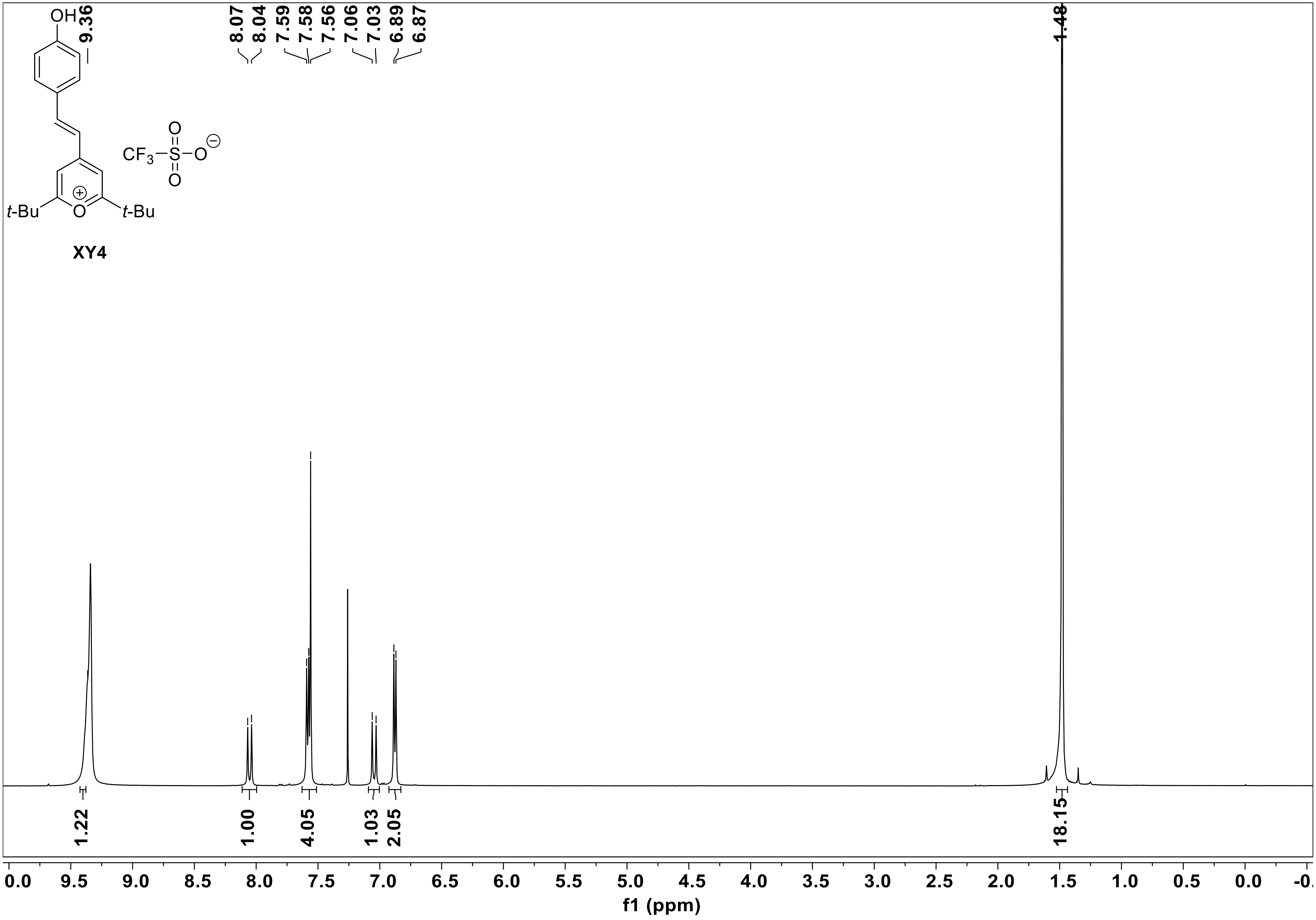

^{13}C NMR Spectrum of XY4 (126 MHz, $CDCl_3$+TFA)

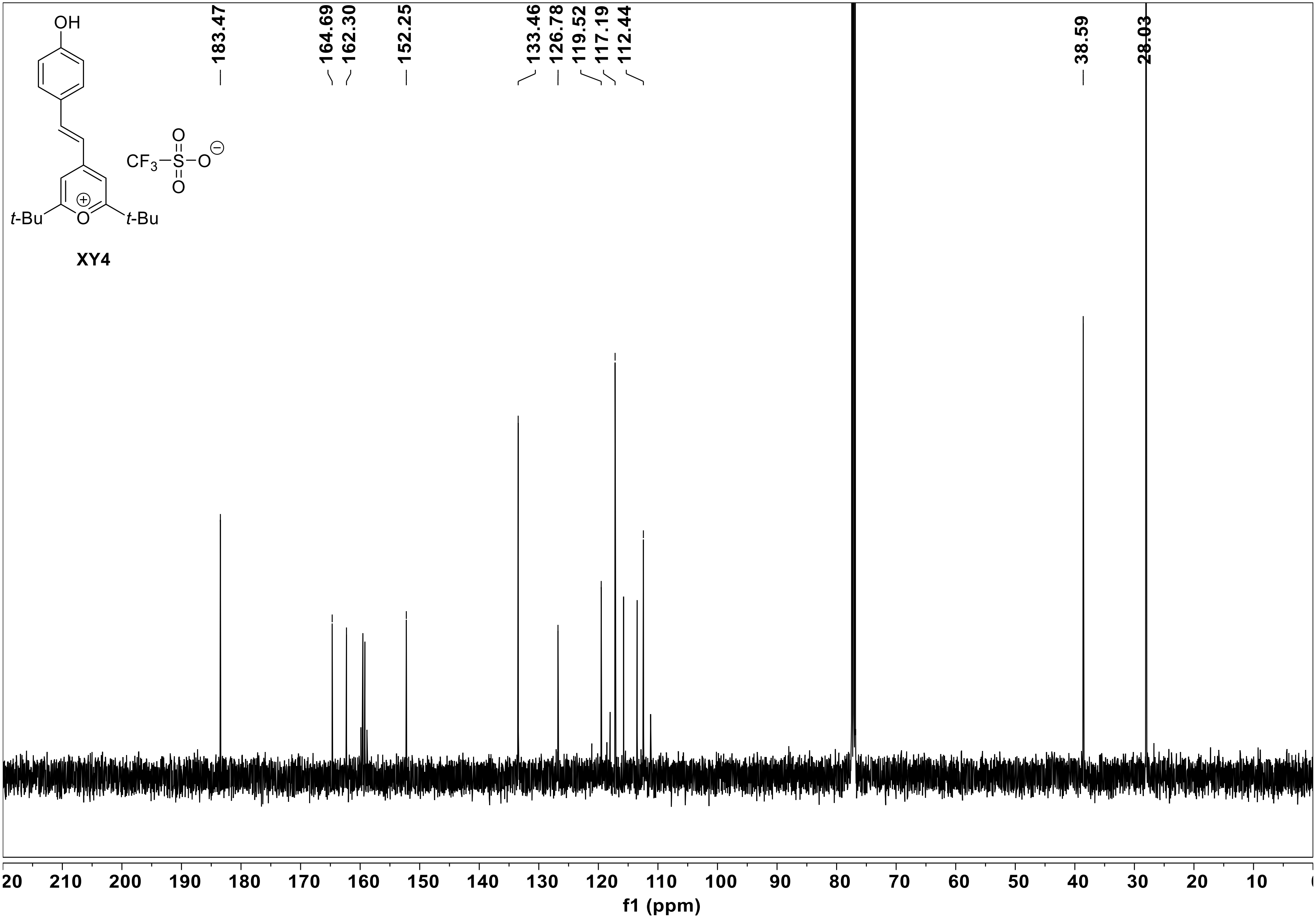

^{19}F NMR Spectrum of XY4 (282 MHz, $CDCl_3$)

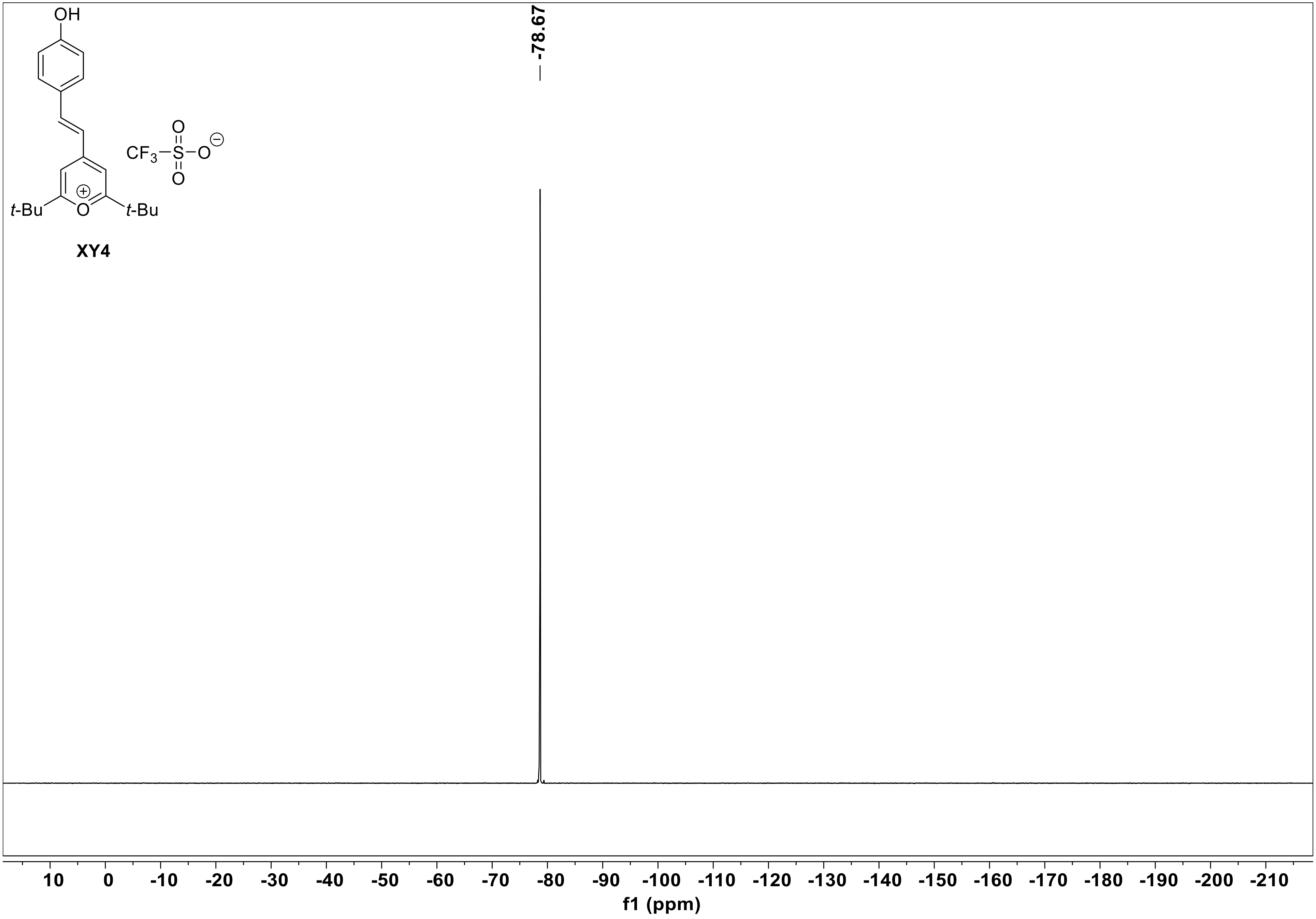

HR-MS Spectra of XY6

Best	ID Source	Name	Formula	Species	m/z	Score	Score (RT)	RT Diff	Diff (ppm)	Score (Lib)	Score (DB)	Score (MFG)
TRUE	MFG		C21 H26 F O	M+	313.1972	92.05			-1.76			92.05

Species	m/z	Score (iso. abund)	Score (mass)	Score (MFG, MS/MS)	Score (MS)	Score (MFG)	Score (iso. spacing)	Height	Ion Formula
M+	313.1972	91.16	97.87		92.05	92.05	81.5	4891910.5	C21 H26 F O

Height (Calc)	Height Sum% (Calc)	Height % (Calc)	m/z (Calc)	Diff (mDa)	Height	Height %	Height Sum %	m/z	Diff (ppm)
5083429.4	79.5	100	313.1962	-1	4891910.5	100	76.5	313.1972	-3.16
1171738.4	18.3	23.1	314.1996	0.5	1301017.5	26.6	20.3	314.1991	1.48
139245	2.2	2.7	315.2028	3.5	201484.8	4.1	3.2	315.1993	11.15

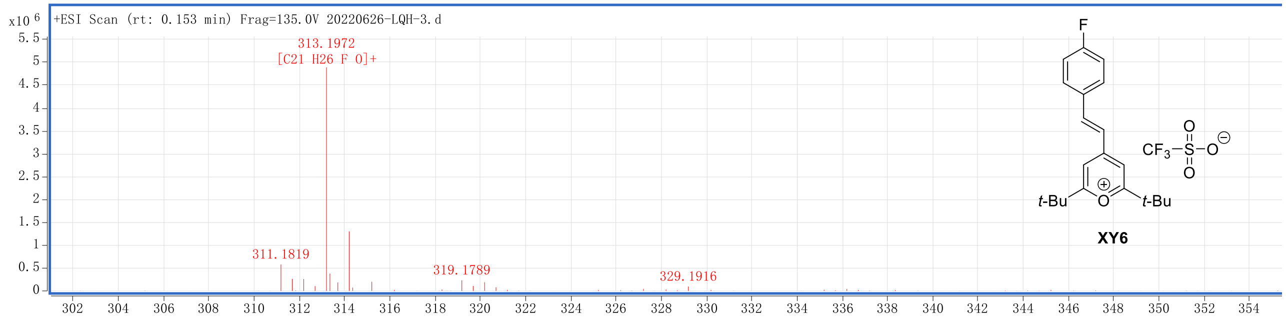

Best	ID Source	Name	Formula	Species	m/z	Score	Score (RT)	RT Diff	Diff (ppm)	Score (Lib)	Score (DB)	Score (MFG)
TRUE	MFG		C F3 O3 S	M-	148.9527	99.32			-1.14			99.32

Species	m/z	Score (iso. abund)	Score (mass)	Score (MFG, MS/MS)	Score (MS)	Score (MFG)	Score (iso. spacing)	Height	Ion Formula
M-	148.9527	98.25	99.64		99.32	99.32	99.96	1339427.4	C F3 O3 S

Height (Calc)	Height Sum% (Calc)	Height % (Calc)	m/z (Calc)	Diff (mDa)	Height	Height %	Height Sum %	m/z	Diff (ppm)
1322174.5	93.4	100	148.9526	-0.2	1339427.4	100	94.6	148.9527	-1.08
26250.5	1.9	2	149.9544	-0.2	22640.9	1.7	1.6	149.9546	-1.06
67449.1	4.8	5.1	150.9494	-0.4	53805.9	4	3.8	150.9498	-2.63

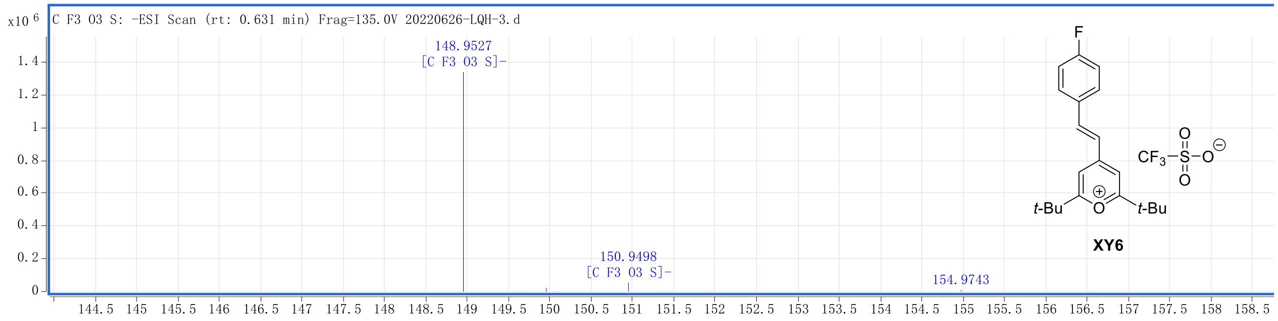

^{1}H NMR Spectrum of XY6 (500 MHz, $CDCl_3$)

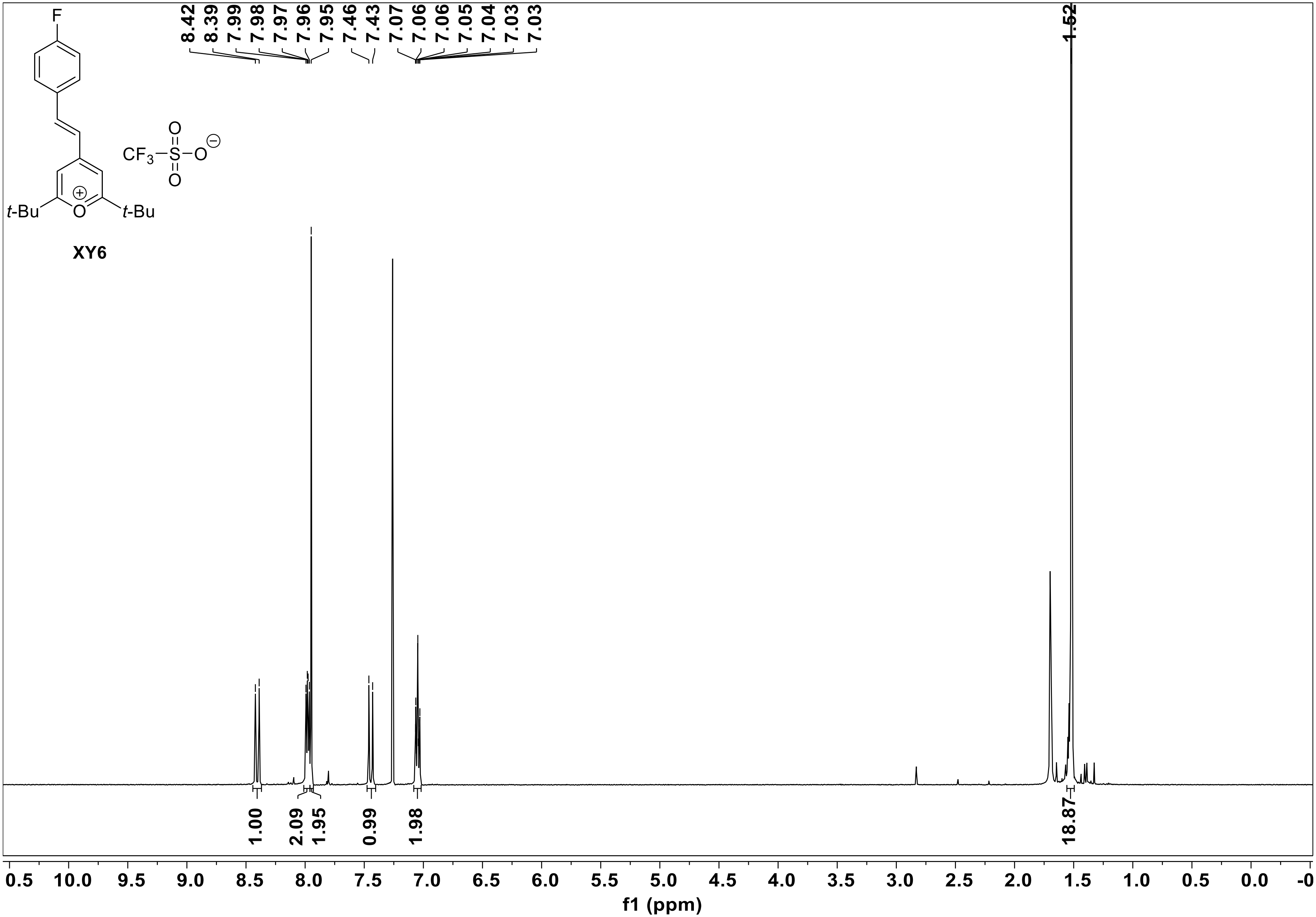

^{13}C NMR Spectrum of XY6 (126 MHz, $CDCl_3$)

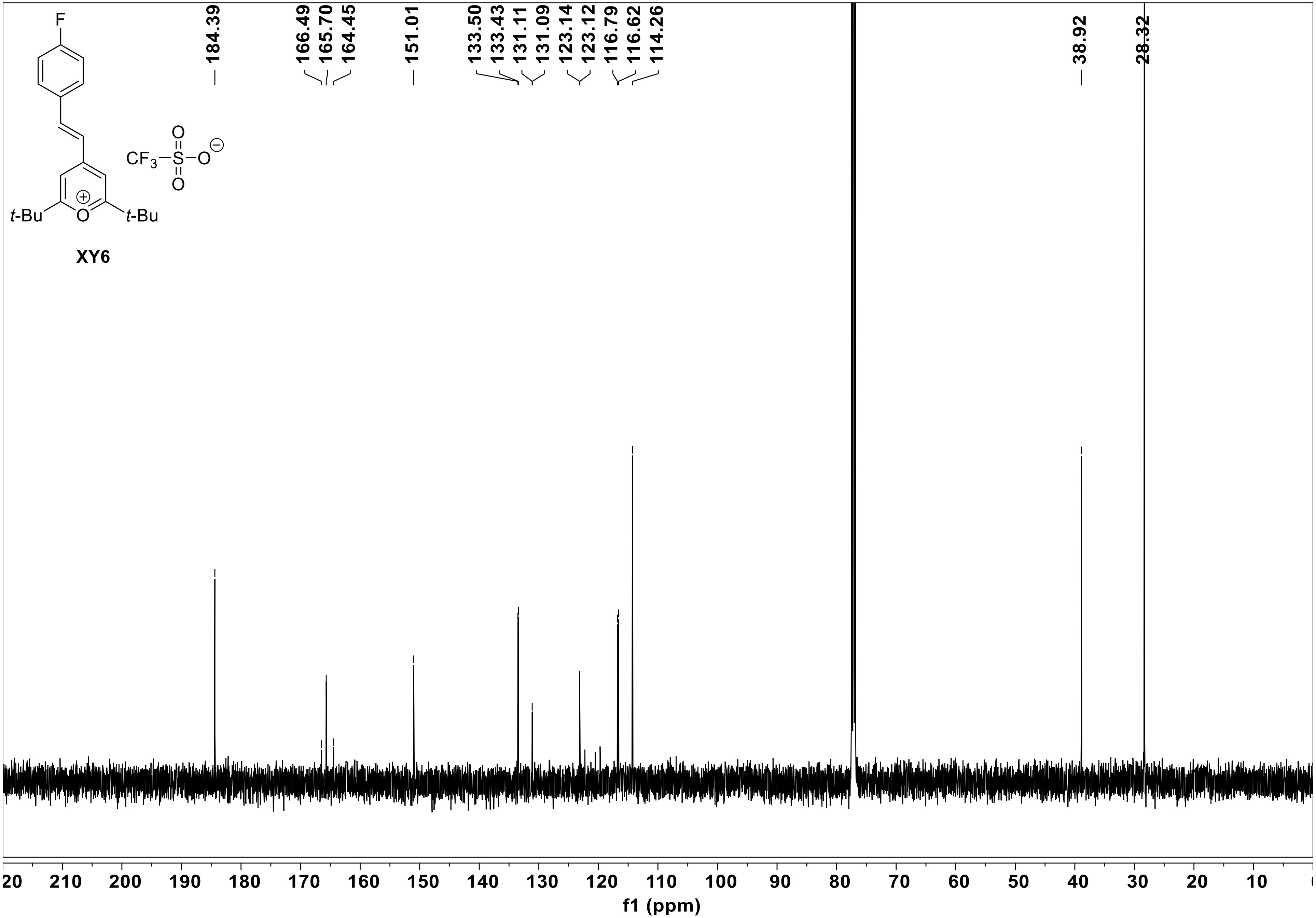

^{19}F NMR Spectrum of XY6 (282 MHz, $CDCl_3$)

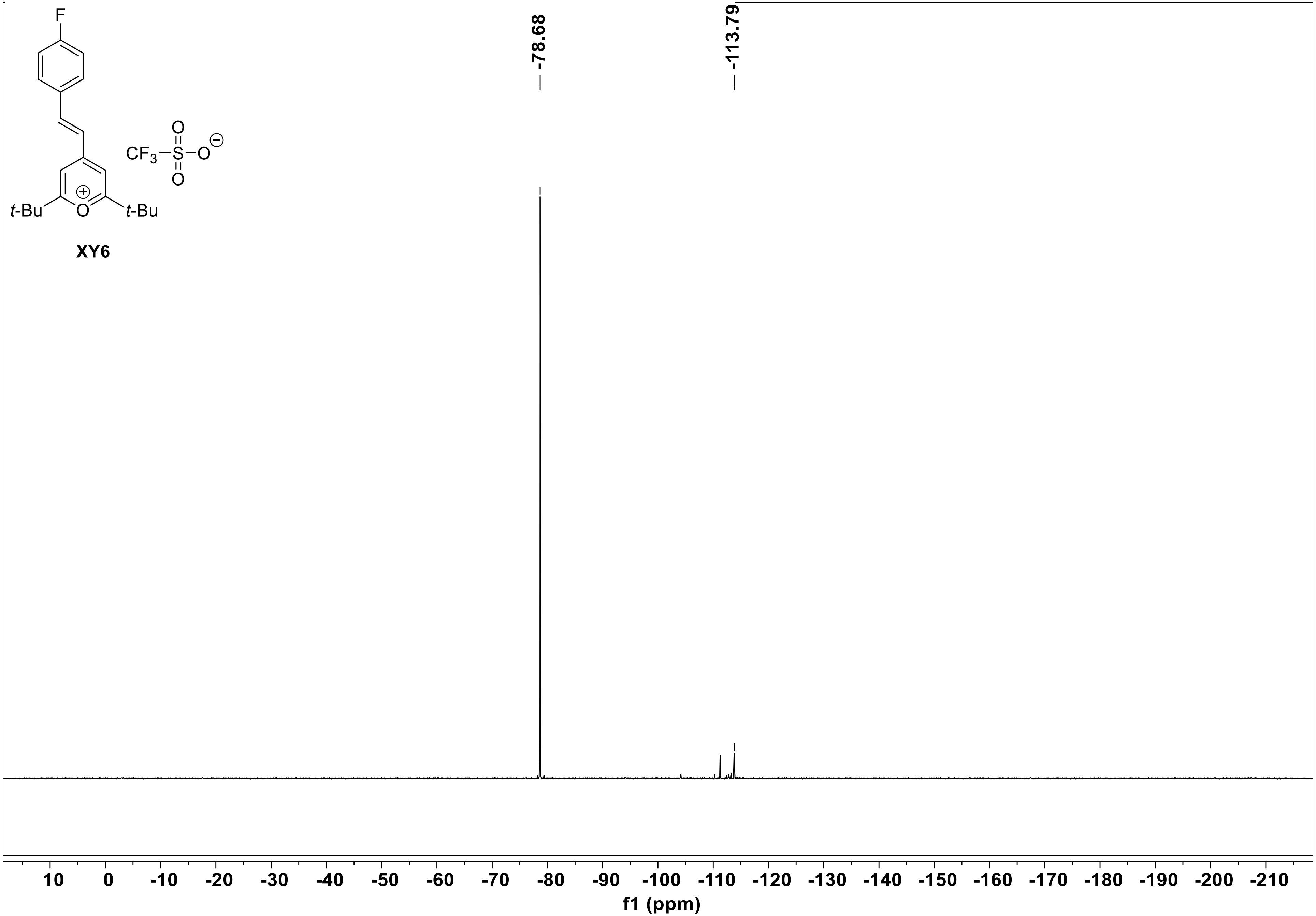

HR-MS Spectra of XY7

Best	ID Source	Name	Formula	Species	m/z	Score	Score (RT)	RT Diff	Diff (ppm)	Score (Lib)	Score (DB)	Score (MFG)
TRUE	MFG		C22 H26 N O	M+	320.2015	94.47			-1.23			94.47

Species	m/z	Score (iso. abund)	Score (mass)	Score (MFG, MS/MS)	Score (MS)	Score (MFG)	Score (iso. spacing)	Height	Ion Formula
M+	320.2015	85.1	98.93		94.47	94.47	96.8	4409625.5	C22 H26 N O

Height (Calc)	Height Sum% (Calc)	Height % (Calc)	m/z (Calc)	Diff (mDa)	Height	Height %	Height Sum %	m/z	Diff (ppm)
4596146.4	78.4	100	320.2009	-0.6	4409625.5	100	75.2	320.2015	-1.81
1125920.8	19.2	24.5	321.2042	0.1	1299314.4	29.5	22.2	321.204	0.46
141407.8	2.4	3.1	322.2073	0.4	154535	3.5	2.6	322.2069	1.3

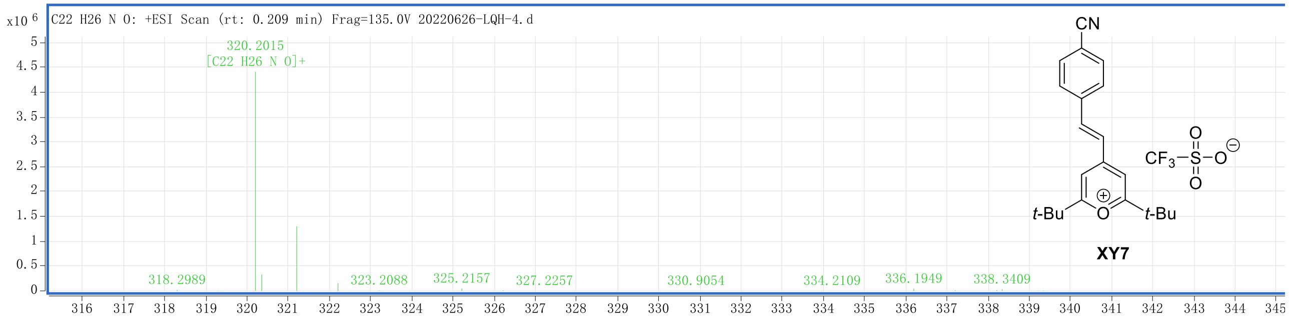

Best	ID Source	Name	Formula	Species	m/z	Score	Score (RT)	RT Diff	Diff (ppm)	Score (Lib)	Score (DB)	Score (MFG)
TRUE	MFG		C F3 O3 S	M-	148.9530	99.09			-2.45			99.09

Species	m/z	Score (iso. abund)	Score (mass)	Score (MFG, MS/MS)	Score (MS)	Score (MFG)	Score (iso. spacing)	Height	Ion Formula
M-	148.953	99.73	98.36		99.09	99.09	99.79	3362944.5	C F3 O3 S

Height (Calc)	Height Sum% (Calc)	Height % (Calc)	m/z (Calc)	Diff (mDa)	Height	Height %	Height Sum %	m/z	Diff (ppm)
3348067.5	93.4	100	148.9526	-0.4	3362944.5	100	93.8	148.953	-2.6
66472.8	1.9	2	149.9544	-0.2	65488.8	1.9	1.8	149.9546	-1.57
170797.5	4.8	5.1	150.9494	0.1	156904.5	4.7	4.4	150.9493	0.46

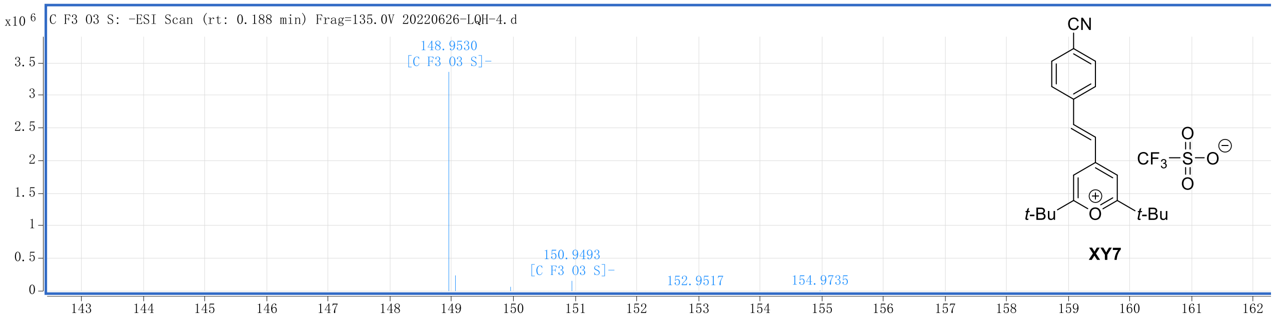

^{1}H NMR Spectrum of XY7 (500 MHz, $CDCl_3$)

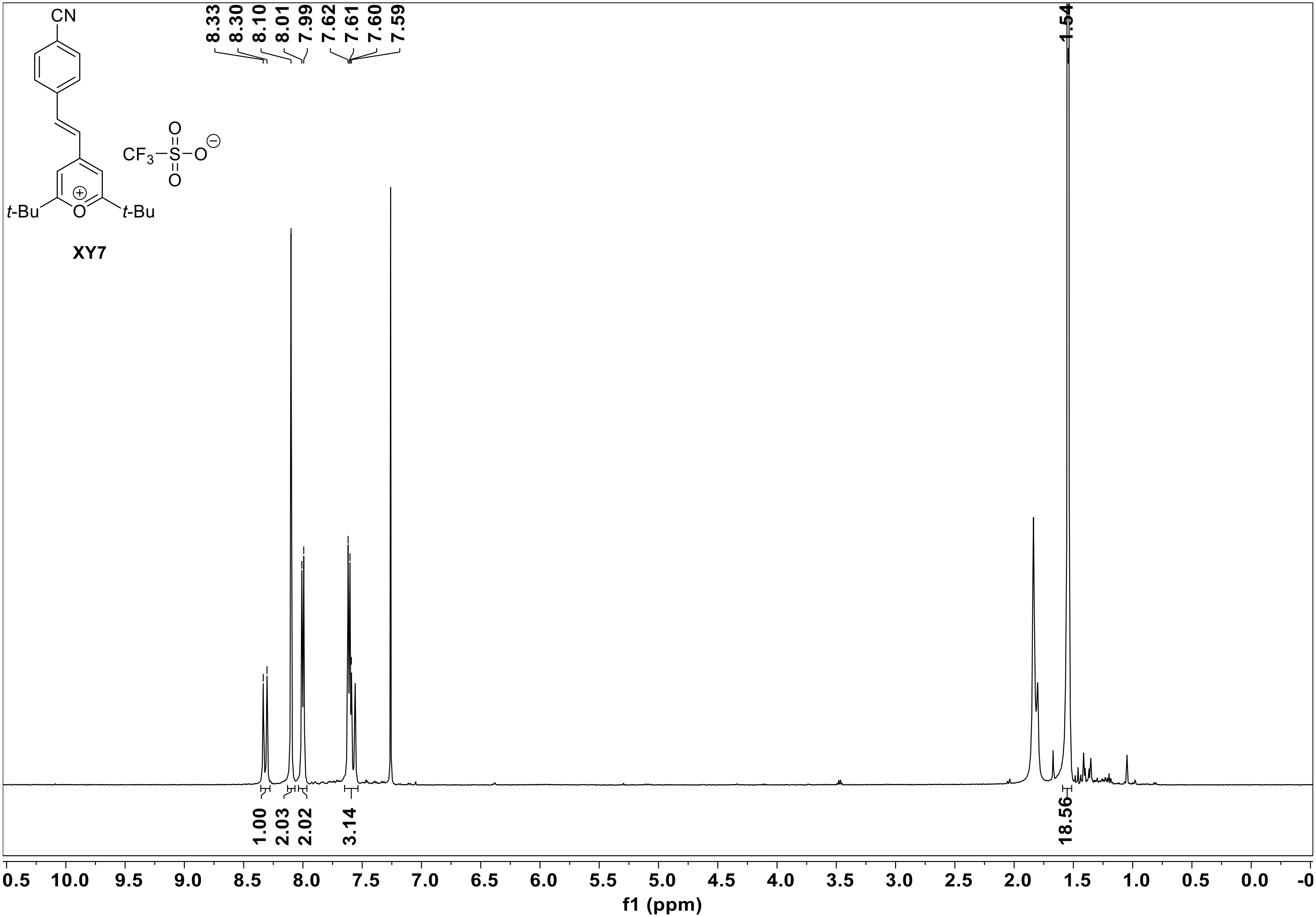

^{13}C NMR Spectrum of XY7 (126 MHz, $CDCl_3$)

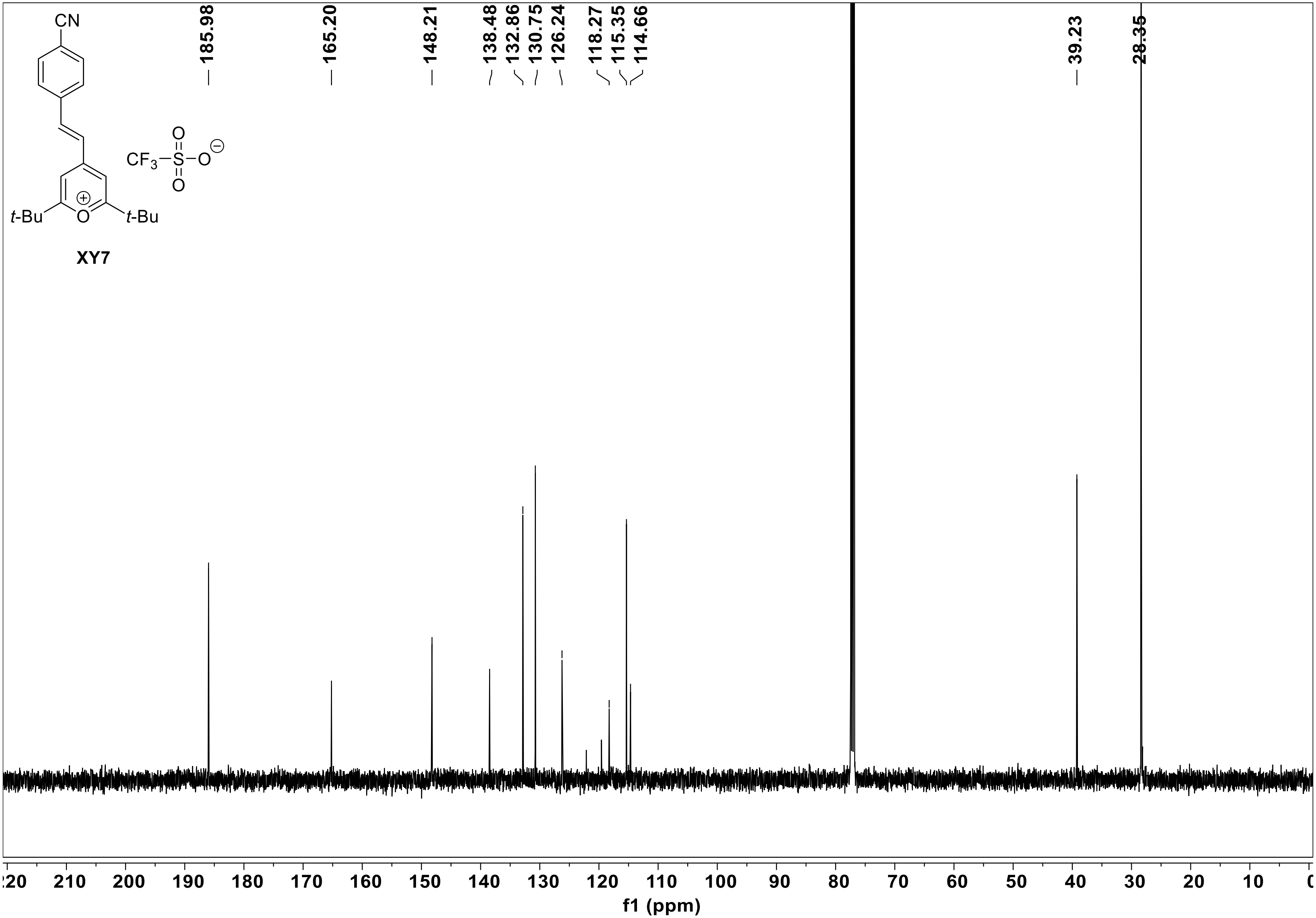

^{19}F NMR Spectrum of XY7 (282 MHz, $CDCl_3$)

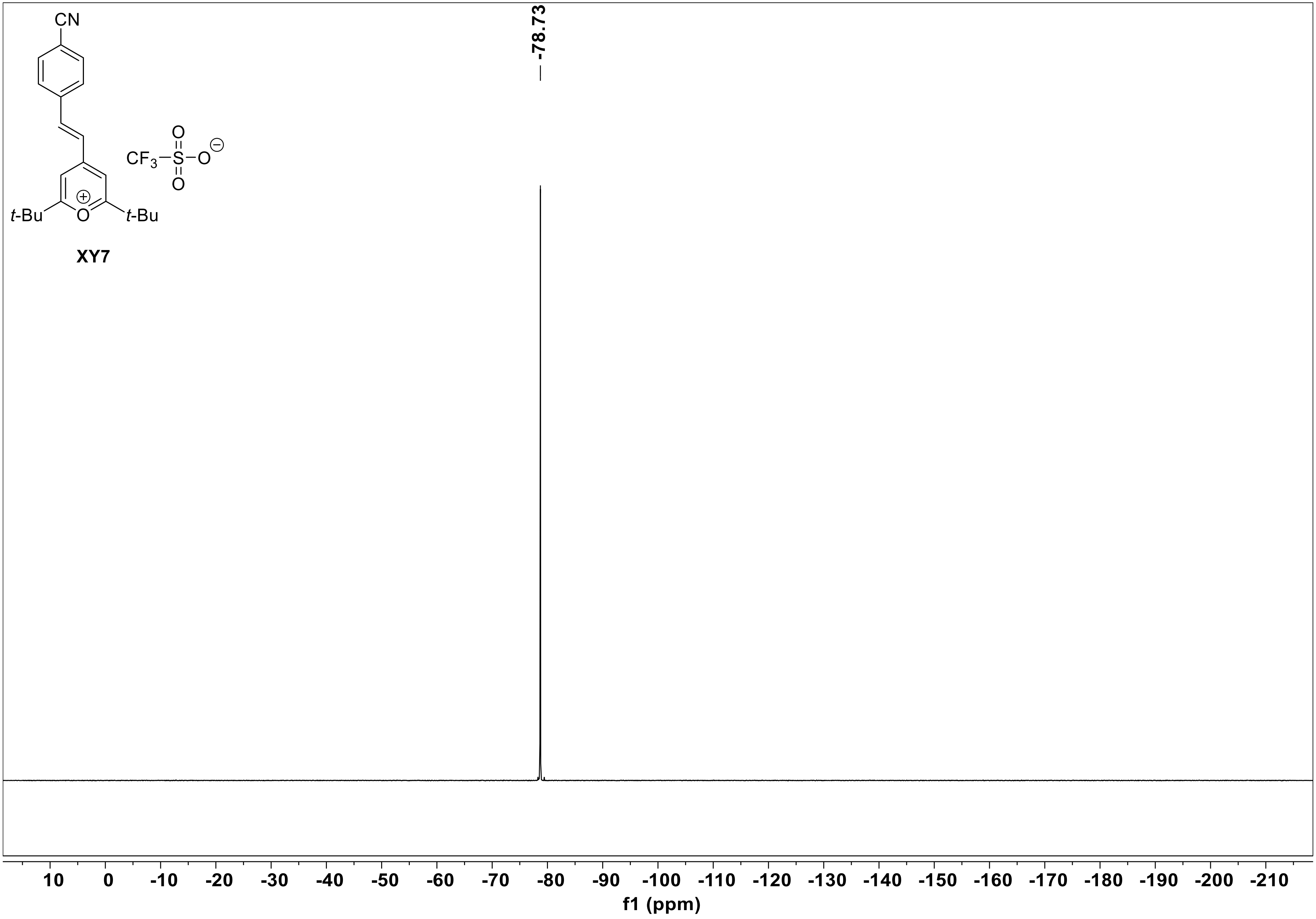

HR-MS Spectra of XY8

Best	ID Source	Name	Formula	Species	m/z	Score	Score (RT)	RT Diff	Diff (ppm)	Score (Lib)	Score (DB)	Score (MFG)
TRUE	MFG		C22 H29 O3 S	M+	373.1839	92.1			-0.85			92.1

Species	m/z	Score (iso. abund)	Score (mass)	Score (MFG, MS/MS)	Score (MS)	Score (MFG)	Score (iso. spacing)	Height	Ion Formula
M+	373.1839	81.71	99.4		92.1	92.1	89.96	5030566.5	C22 H29 O3 S

Height (Calc)	Height Sum% (Calc)	Height % (Calc)	m/z (Calc)	Diff (mDa)	Height	Height %	Height Sum %	m/z	Diff (ppm)
5250349	75.1	100	373.1832	-0.7	5030566.5	100	72	373.1839	-1.79
1314266.1	18.8	25	374.1865	0.9	1522209.8	30.3	21.8	374.1856	2.28
424848.6	6.1	8.1	375.1836	-0.3	436687.4	8.7	6.2	375.1839	-0.88

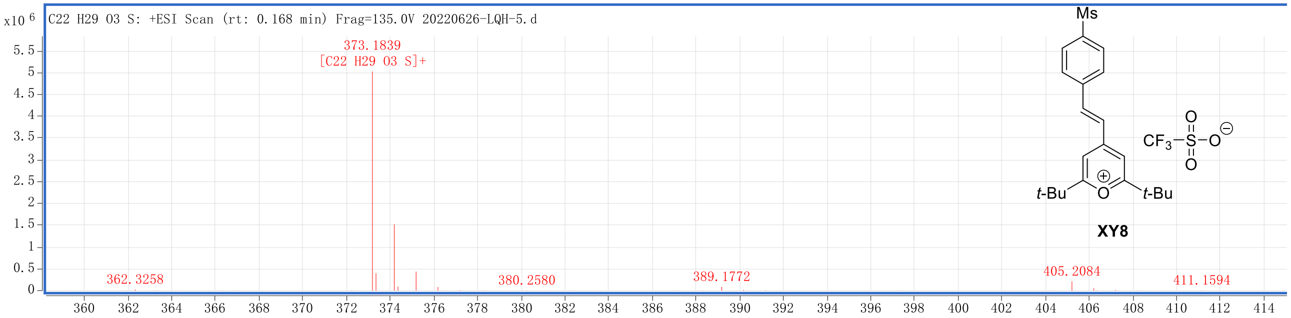

Best	ID Source	Name	Formula	Species	m/z	Score	Score (RT)	RT Diff	Diff (ppm)	Score (Lib)	Score (DB)	Score (MFG)
TRUE	MFG		C F3 O3 S	M-	148.9527	99.17			-1.06			99.17

Species	m/z	Score (iso. abund)	Score (mass)	Score (MFG, MS/MS)	Score (MS)	Score (MFG)	Score (iso. spacing)	Height	Ion Formula
M-	148.9527	97.67	99.69		99.17	99.17	99.94	1273708.1	C F3 O3 S

Height (Calc)	Height Sum% (Calc)	Height % (Calc)	m/z (Calc)	Diff (mDa)	Height	Height %	Height Sum %	m/z	Diff (ppm)
1255680.6	93.4	100	148.9526	-0.1	1273708.1	100	94.7	148.9527	-0.99
24930.4	1.9	2	149.9544	-0.6	22163.2	1.7	1.6	149.955	-3.8
64057	4.8	5.1	150.9494	-0.3	48796.7	3.8	3.6	150.9497	-1.66

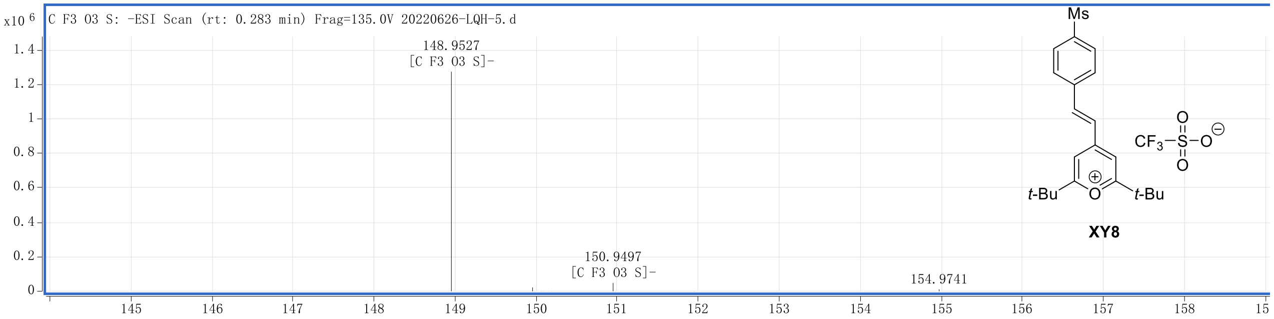

^{1}H NMR Spectrum of XY8 (500 MHz, $CDCl_3$)

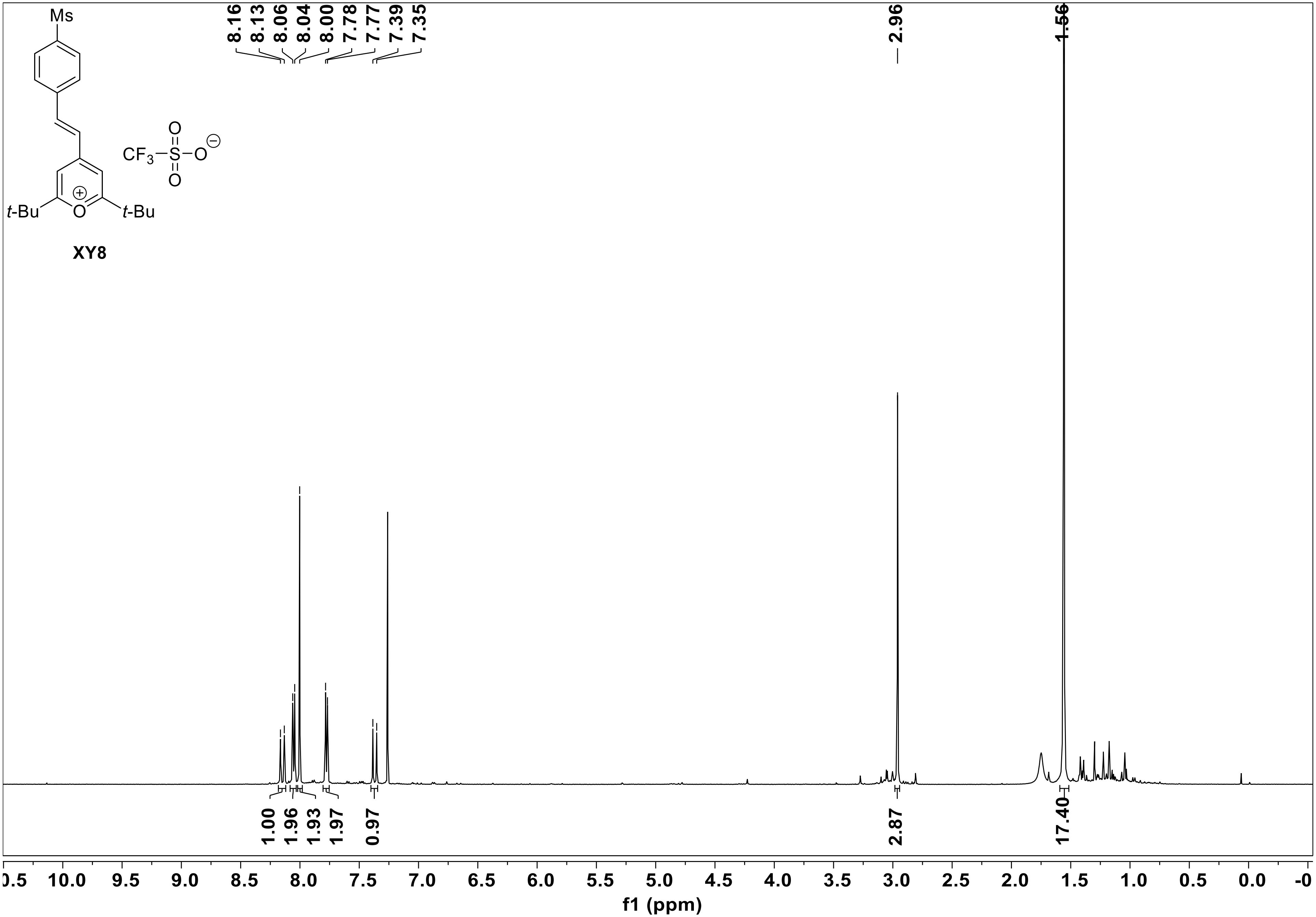

^{13}C NMR Spectrum of XY8 (126 MHz, $CDCl_3$)

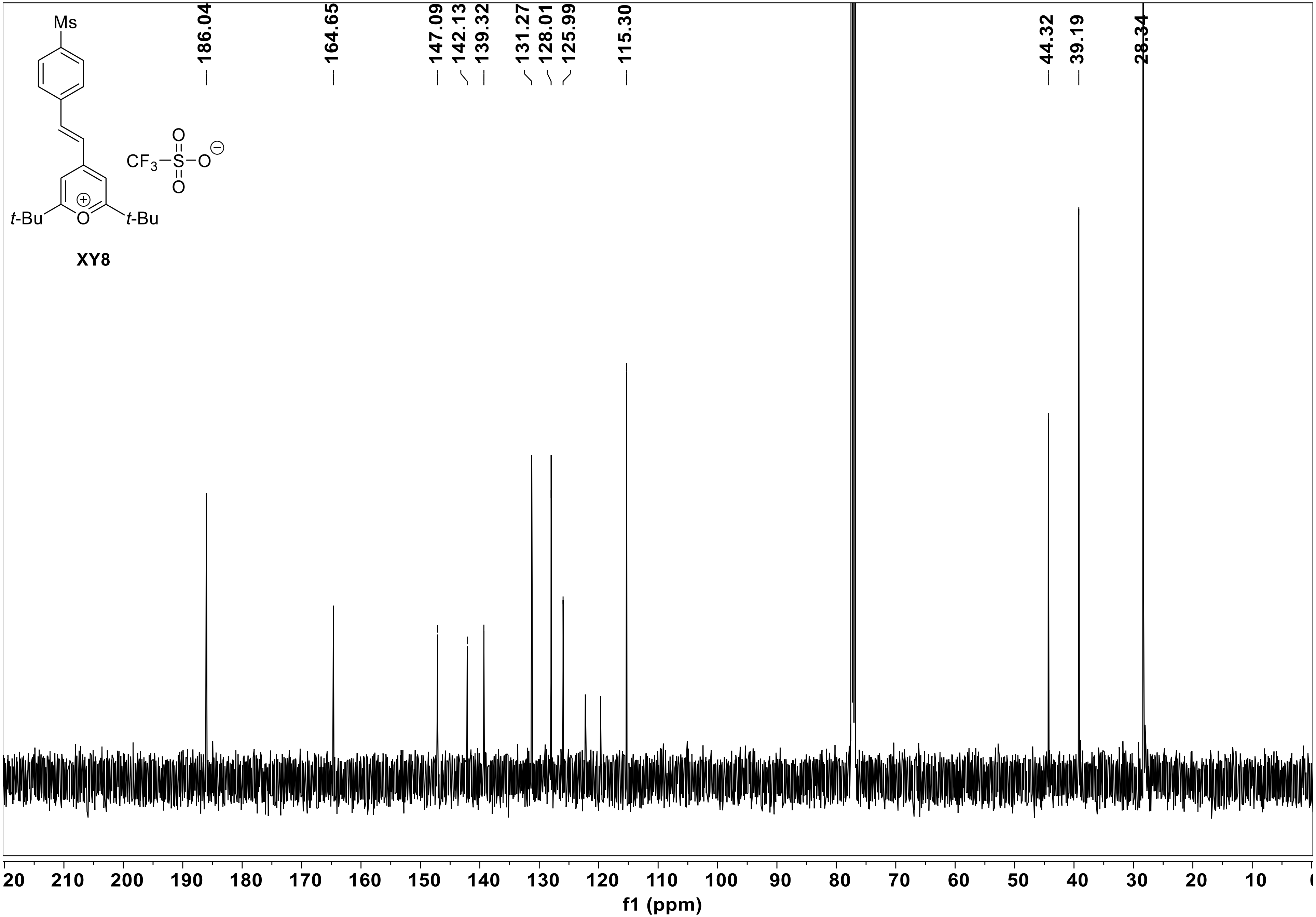

^{19}F NMR Spectrum of XY8 (282 MHz, $CDCl_3$)

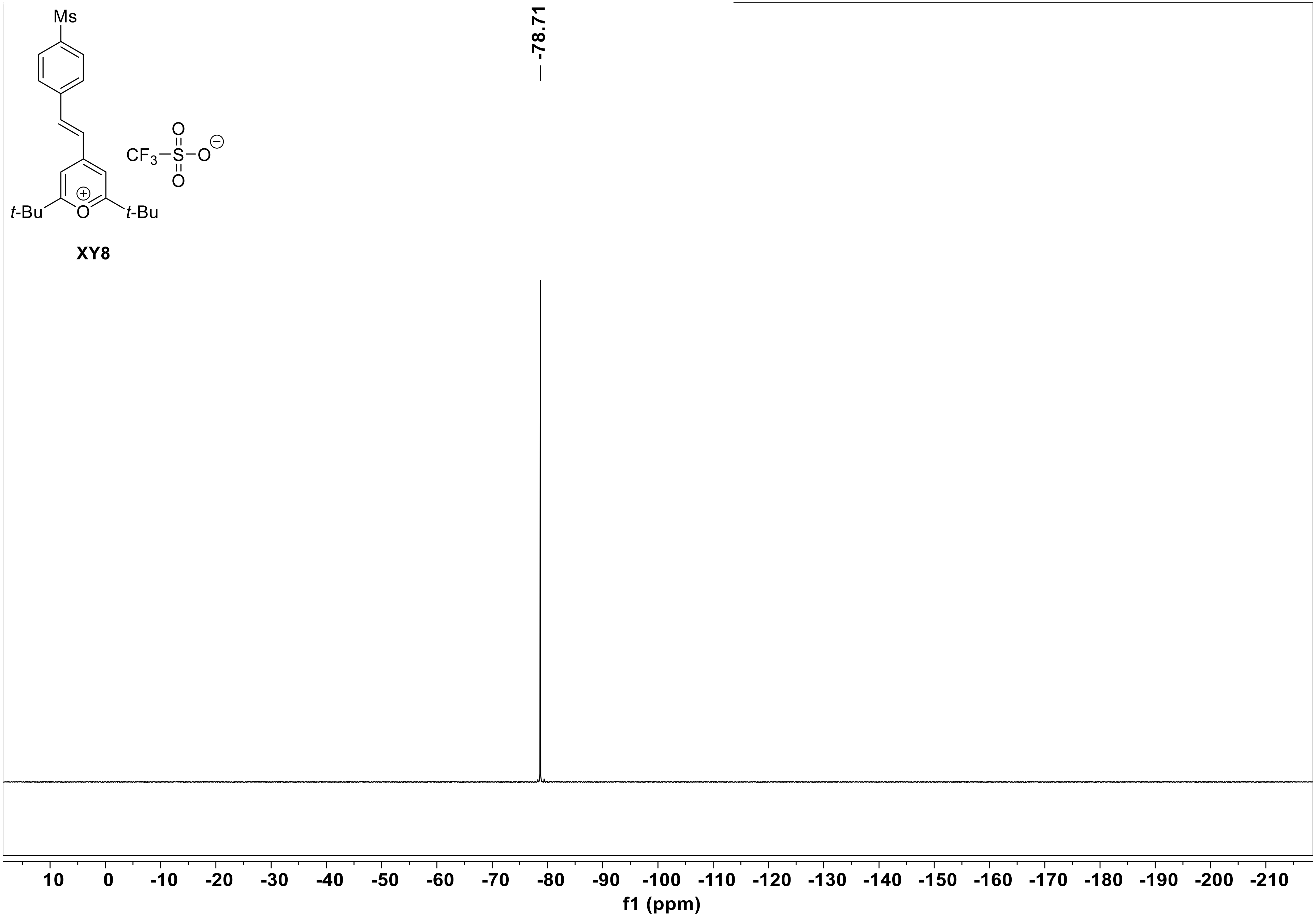

HR-MS Spectra of XY9

Best	ID Source	Name	Formula	Species	m/z	Score	Score (RT)	RT Diff	Diff (ppm)	Score (Lib)	Score (DB)	Score (MFG)
TRUE	MFG		C23 H29 O3	M+	353.2118	91.69			-0.89			91.69

Species	m/z	Score (iso. abund)	Score (mass)	Score (MFG, MS/MS)	Score (MS)	Score (MFG)	Score (iso. spacing)	Height	Ion Formula
M+	353.2118	78.48	99.38		91.69	91.69	92.16	6726917.5	C23 H29 O3

Height (Calc)	Height Sum% (Calc)	Height % (Calc)	m/z (Calc)	Diff (mDa)	Height	Height %	Height Sum %	m/z	Diff (ppm)
7113221.6	77.5	100	353.2111	-0.6	6726917.5	100	73.3	353.2118	-1.82
1801351.5	19.6	25.3	354.2145	0.5	2128113.2	31.6	23.2	354.214	1.51
262367.8	2.9	3.7	355.2175	0.9	321910.2	4.8	3.5	355.2166	2.65

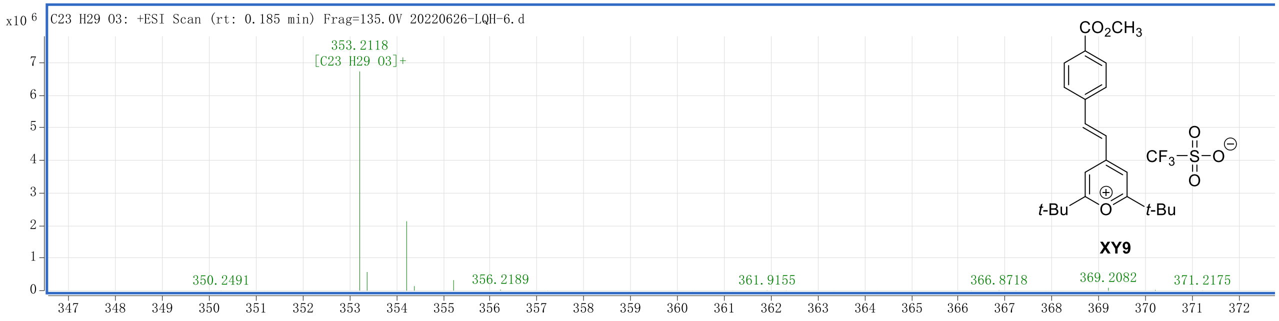

Best	ID Source	Name	Formula	Species	m/z	Score	Score (RT)	RT Diff	Diff (ppm)	Score (Lib)	Score (DB)	Score (MFG)
TRUE	MFG		C F3 O3 S	M-	148.9528	99.33			-1.38			99.33

Species	m/z	Score (iso. abund)	Score (mass)	Score (MFG, MS/MS)	Score (MS)	Score (MFG)	Score (iso. spacing)	Height	Ion Formula
M-	148.9528	98.53	99.47		99.33	99.33	100	1429328.8	C F3 O3 S

Height (Calc)	Height Sum% (Calc)	Height % (Calc)	m/z (Calc)	Diff (mDa)	Height	Height %	Height Sum %	m/z	Diff (ppm)
1414476.8	93.4	100	148.9526	-0.2	1429328.8	100	94.4	148.9528	-1.36
28083.1	1.9	2	149.9544	-0.3	27051.9	1.9	1.8	149.9547	-1.75
72157.8	4.8	5.1	150.9494	-0.2	58337	4.1	3.9	150.9496	-1.62

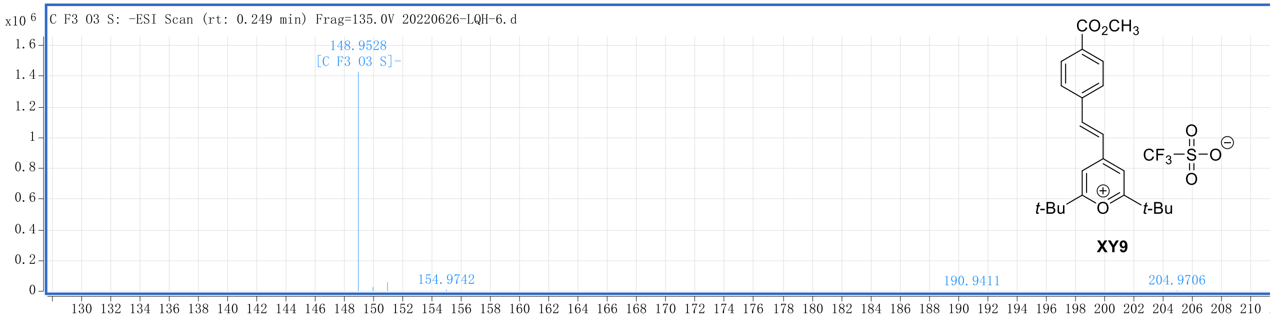

^{1}H NMR Spectrum of XY9 (500 MHz, $CDCl_3$)

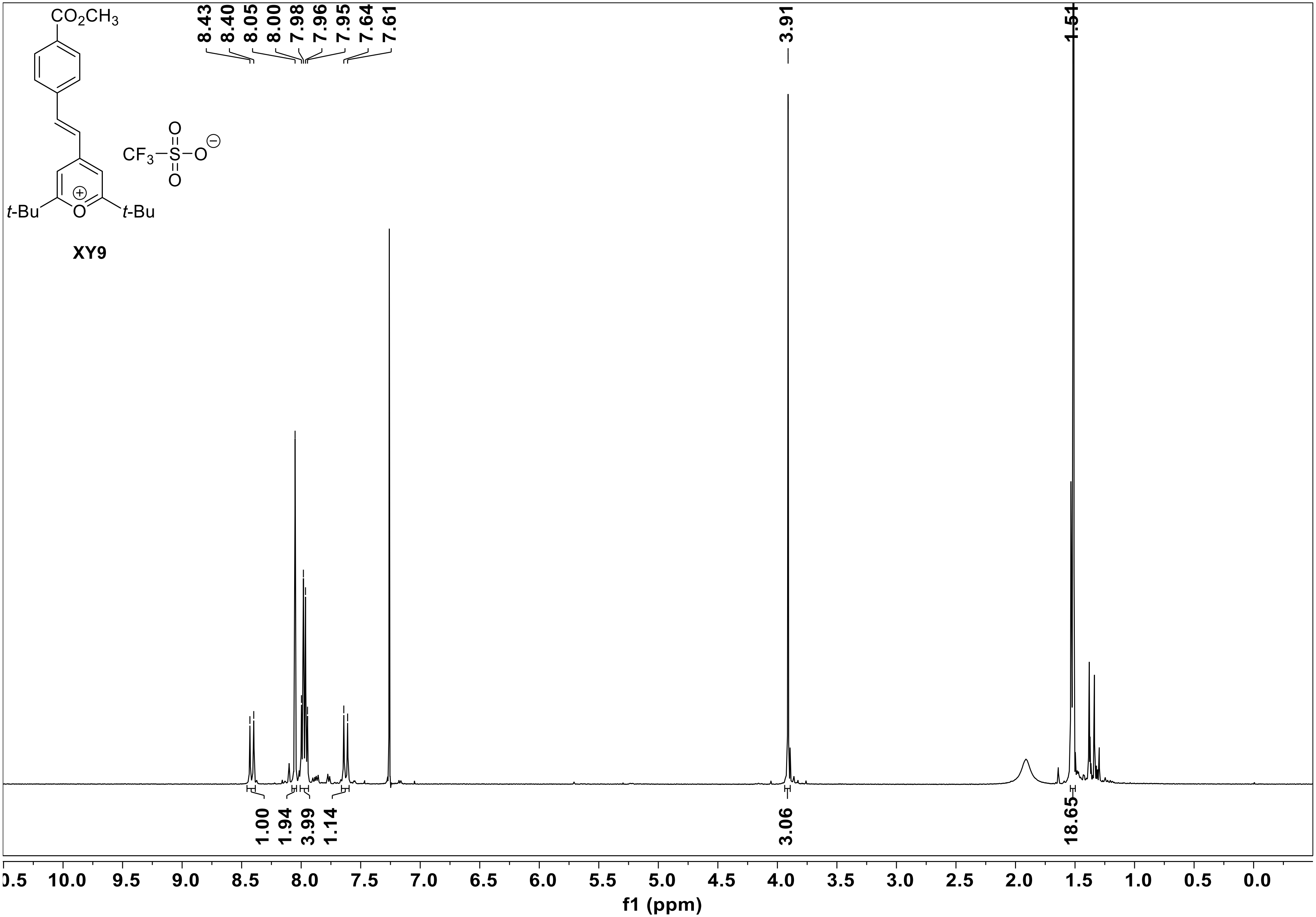

^{13}C NMR Spectrum of XY9 (126 MHz, $CDCl_3$)

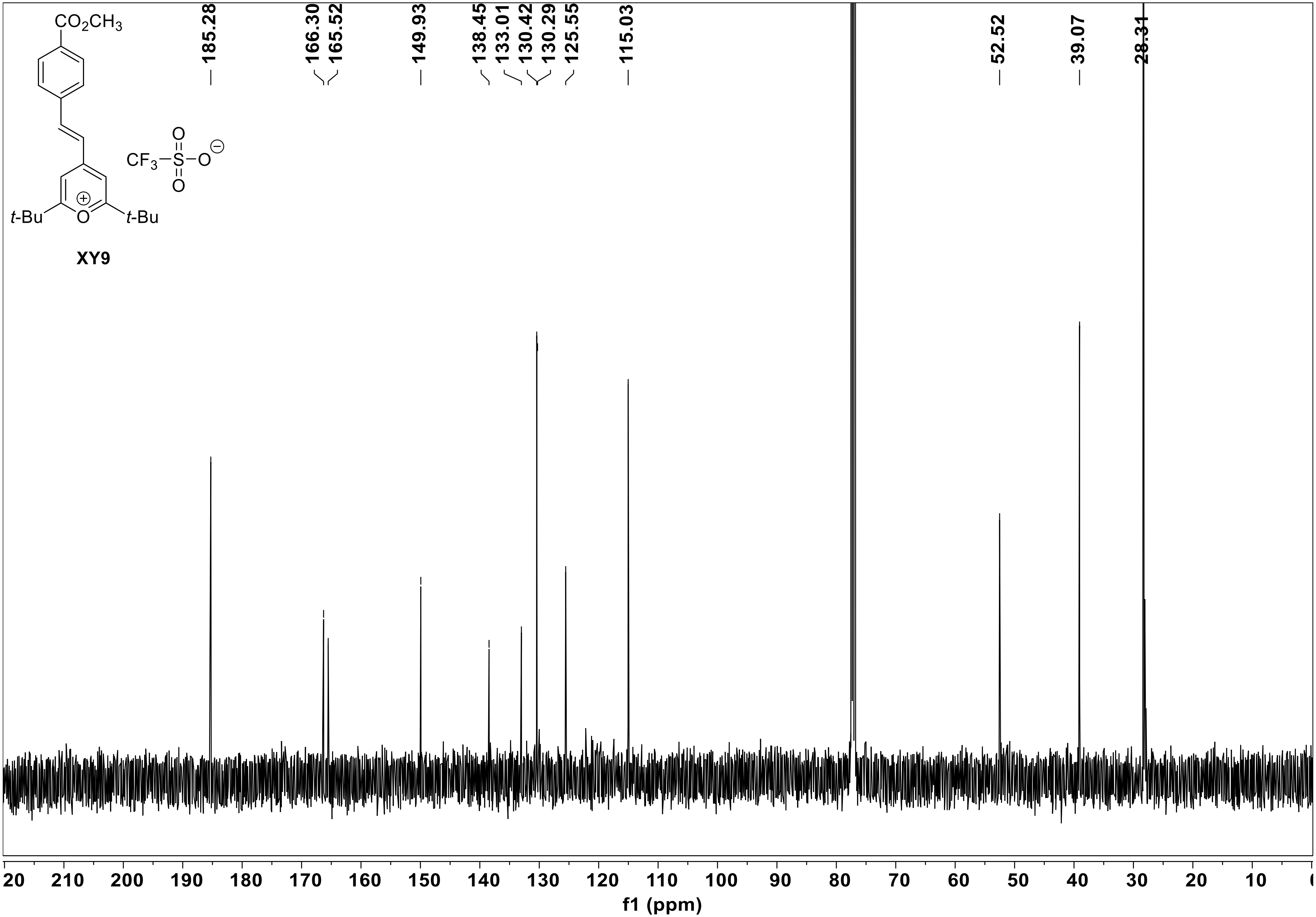

^{19}F NMR Spectrum of XY9 (282 MHz, $CDCl_3$)

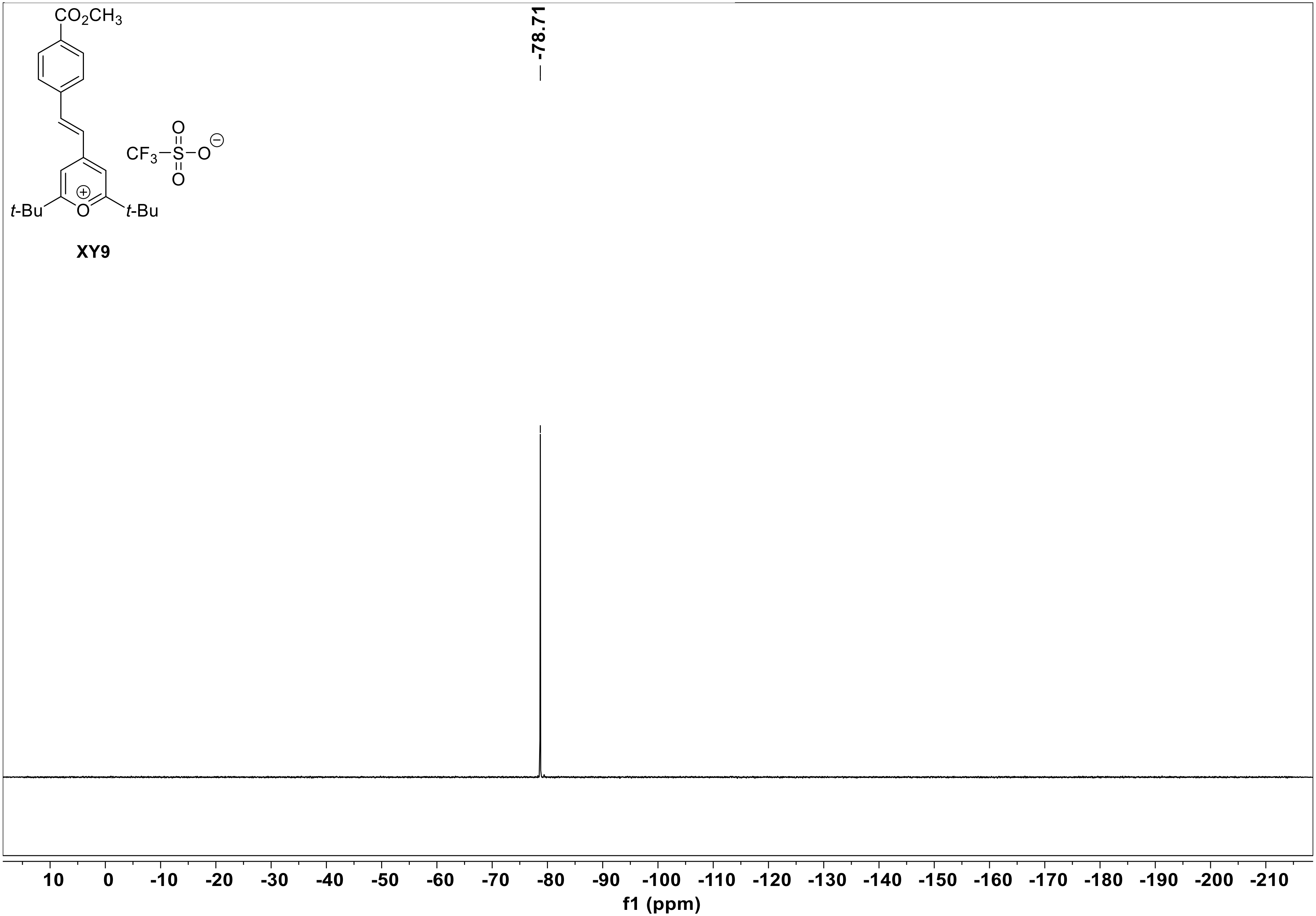

HR-MS Spectra of XY10

Best	ID Source	Name	Formula	Species	m/z	Score	Score (RT)	RT Diff	Diff (ppm)	Score (Lib)	Score (DB)	Score (MFG)
TRUE	MFG		C25 H36 N O	M+	366.2793	90.02			0.52			90.02

Species	m/z	Score (iso. abund)	Score (mass)	Score (MFG, MS/MS)	Score (MS)	Score (MFG)	Score (iso. spacing)	Height	Ion Formula
M+	366.2793	70.9	99.78		90.02	90.02	93.43	7046445.5	C25 H36 N O

Height (Calc)	Height Sum% (Calc)	Height % (Calc)	m/z (Calc)	Diff (mDa)	Height	Height %	Height Sum %	m/z	Diff (ppm)
7537940.9	75.9	100	366.2791	-0.1	7046445.5	100	70.9	366.2793	-0.37
2099828.4	21.1	27.9	367.2825	1	2524554	35.8	25.4	367.2815	2.67
296887.9	3	3.9	368.2856	1.1	363657.7	5.2	3.7	368.2846	2.89

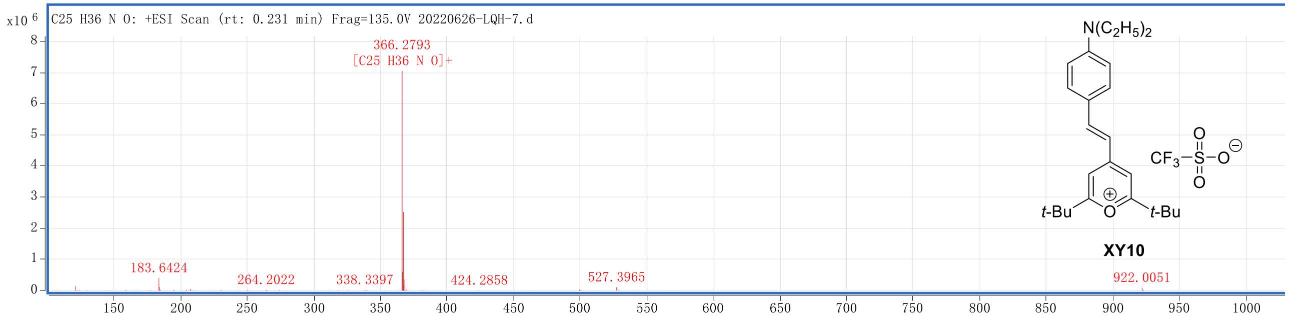

Best	ID Source	Name	Formula	Species	m/z	Score	Score (RT)	RT Diff	Diff (ppm)	Score (Lib)	Score (DB)	Score (MFG)
TRUE	MFG		C F3 O3 S	M-	148.9530	98.6			-2.92			98.6

Species	m/z	Score (iso. abund)	Score (mass)	Score (MFG, MS/MS)	Score (MS)	Score (MFG)	Score (iso. spacing)	Height	Ion Formula
M-	148.953	99.07	97.67		98.6	98.6	99.91	2596434	C F3 O3 S

Height (Calc)	Height Sum% (Calc)	Height % (Calc)	m/z (Calc)	Diff (mDa)	Height	Height %	Height Sum %	m/z	Diff (ppm)
2573064.9	93.4	100	148.9526	-0.5	2596434	100	94.2	148.953	-3.03
51085.8	1.9	2	149.9544	-0.3	47328.5	1.8	1.7	149.9547	-2.04
131261.7	4.8	5.1	150.9494	-0.1	111649.9	4.3	4.1	150.9495	-0.86

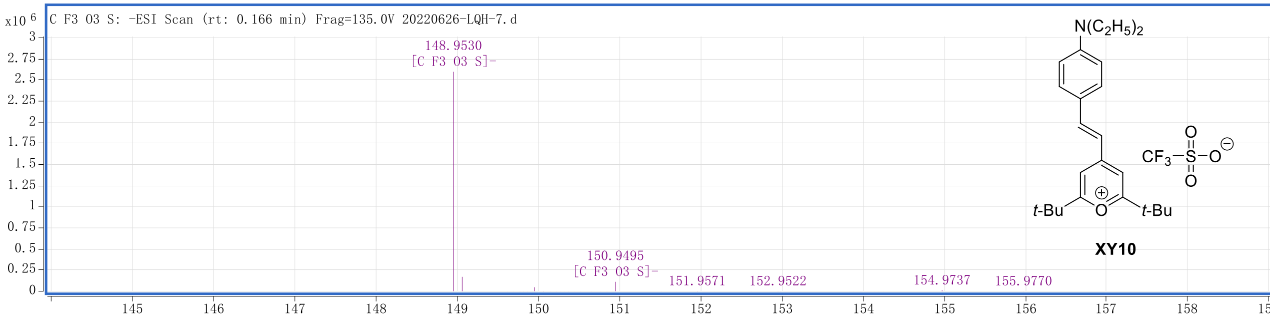

^{1}H NMR Spectrum of XY10 (500 MHz, $CDCl_3$)

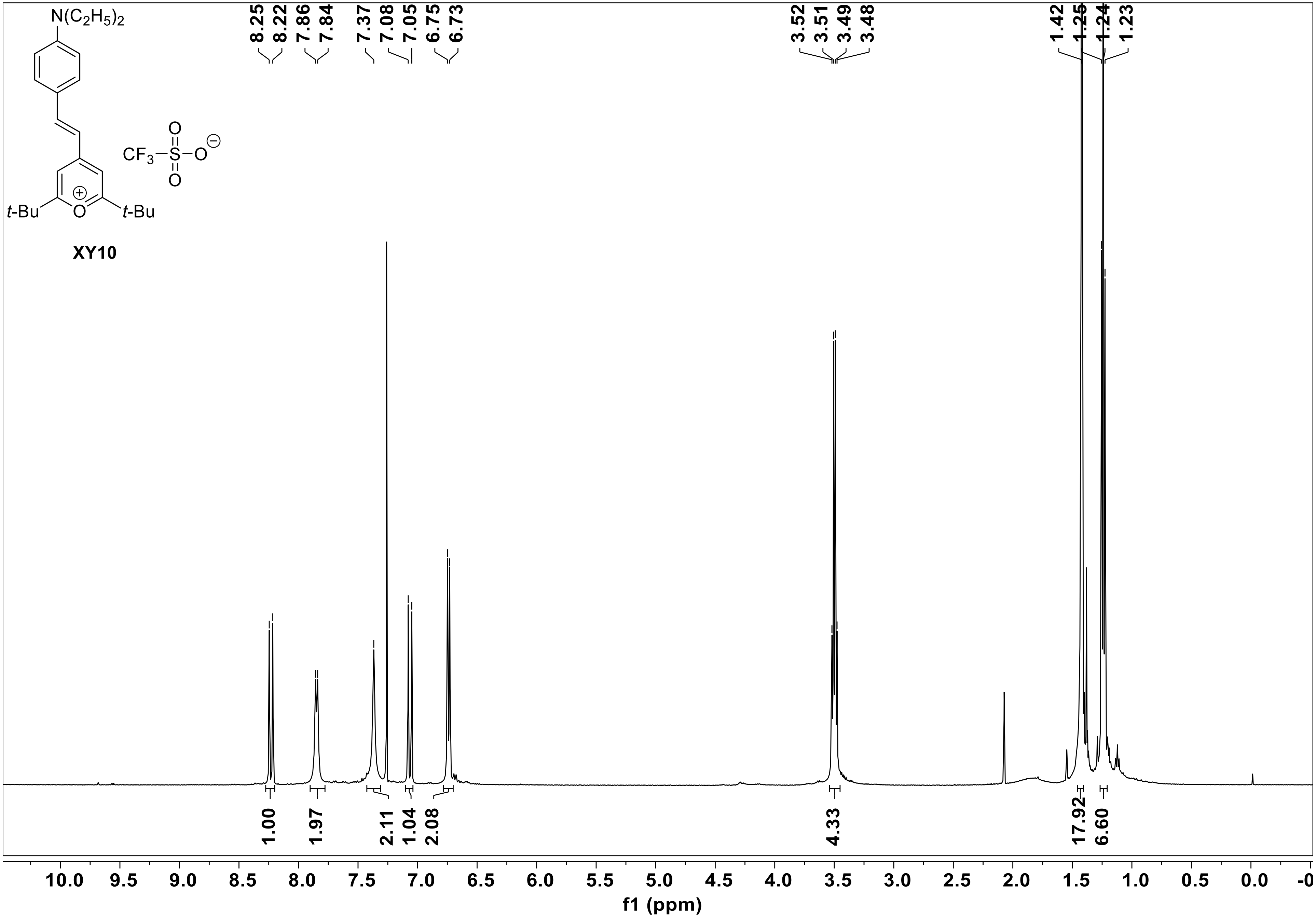

^{13}C NMR Spectrum of XY10 (126 MHz, $CDCl_3$)

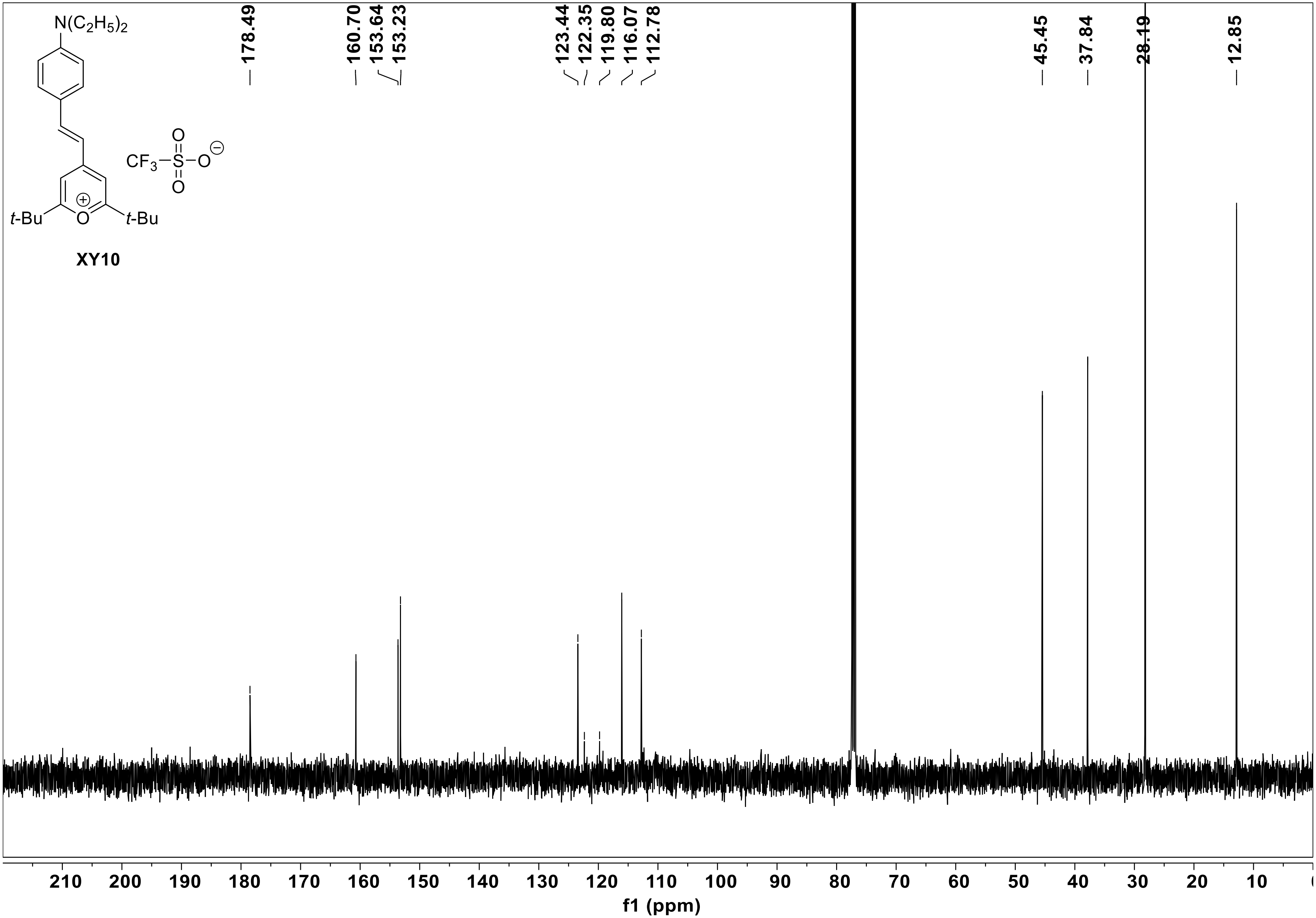

^{19}F NMR Spectrum of XY10 (282 MHz, $CDCl_3$)

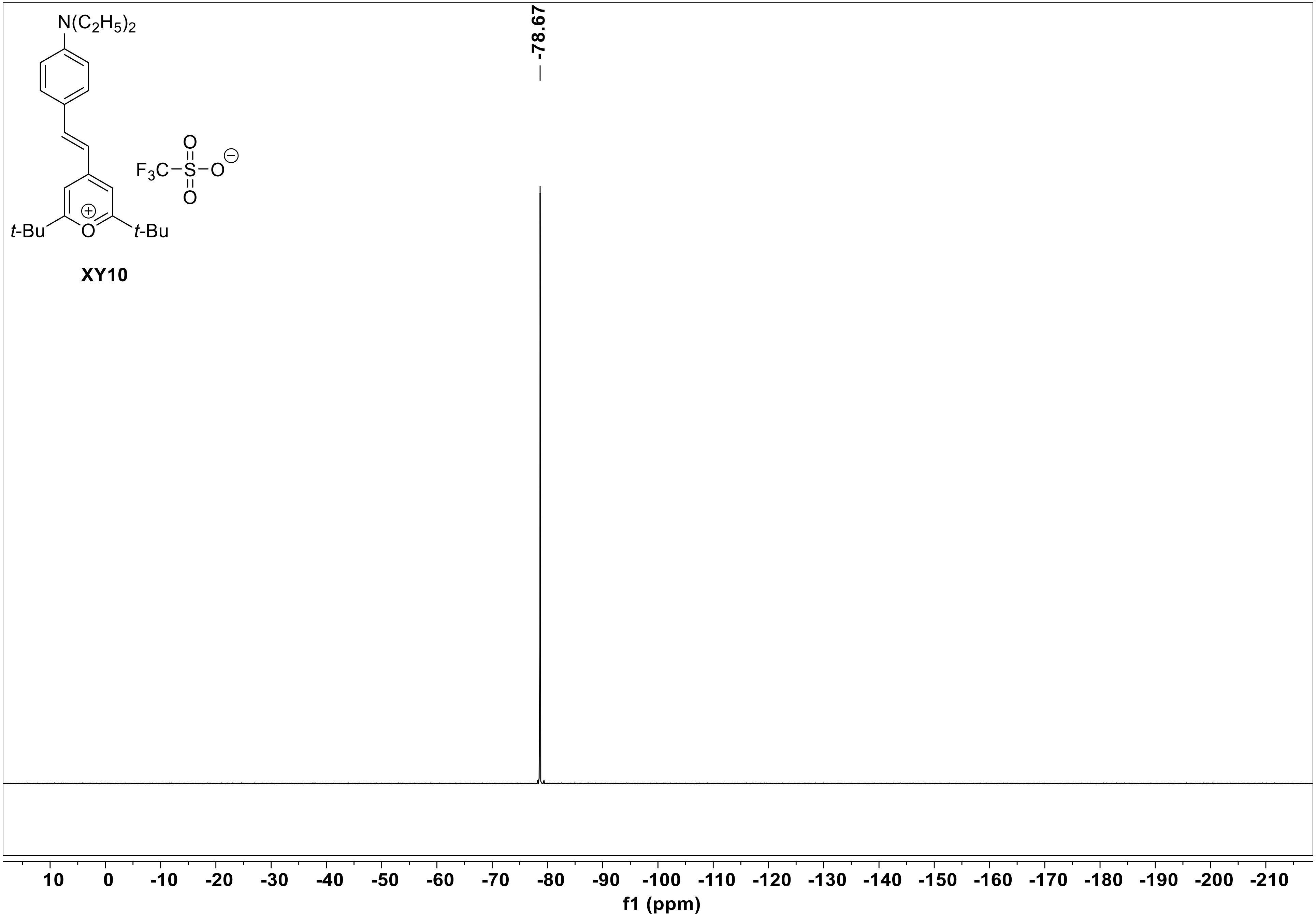

HR-MS Spectra of XY11

Best	ID Source	Name	Formula	Species	m/z	Score	Score (RT)	RT Diff	Diff (ppm)	Score (Lib)	Score (DB)	Score (MFG)
TRUE	MFG		C33 H36 N O	M+	462.2795	77.59			1.07			77.59

Species	m/z	Score (iso. abund)	Score (mass)	Score (MFG, MS/MS)	Score (MS)	Score (MFG)	Score (iso. spacing)	Height	Ion Formula
M+	462.2795	49.33	98.84		77.59	77.59	68.98	10208785	C33 H36 N O

Height (Calc)	Height Sum% (Calc)	Height % (Calc)	m/z (Calc)	Diff (mDa)	Height	Height %	Height Sum %	m/z	Diff (ppm)
11646951.8	69.4	100	462.2791	-0.4	10208785	100	60.9	462.2795	-0.87
4252229.2	25.4	36.5	463.2825	1.5	5201277.5	50.9	31	463.281	3.18
777603.6	4.6	6.7	464.2857	3.5	1238064.1	12.1	7.4	464.2822	7.6
95120	0.6	0.8	465.2889	3	123777.9	1.2	0.7	465.2859	6.47

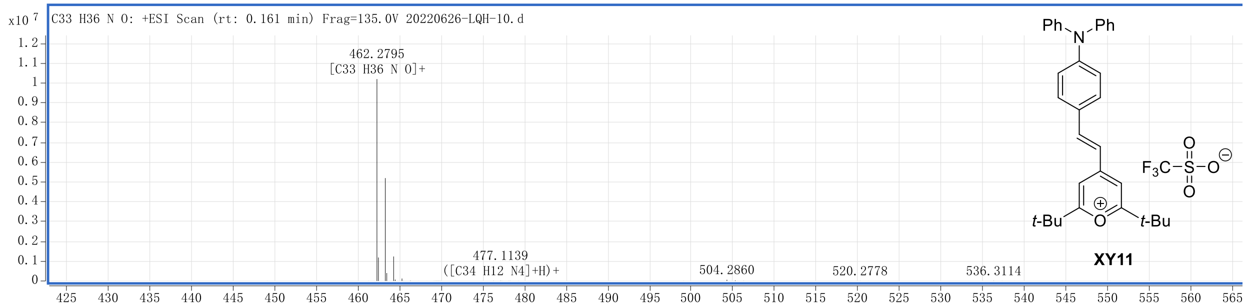

Best	ID Source	Name	Formula	Species	m/z	Score	Score (RT)	RT Diff	Diff (ppm)	Score (Lib)	Score (DB)	Score (MFG)
TRUE	MFG		C F3 O3 S	M-	148.9526	99.45			0.08			99.45

Species	m/z	Score (iso. abund)	Score (mass)	Score (MFG, MS/MS)	Score (MS)	Score (MFG)	Score (iso. spacing)	Height	Ion Formula
M-	148.9526	98.1	100		99.45	99.45	99.98	1273258.4	C F3 O3 S

Height (Calc)	Height Sum% (Calc)	Height % (Calc)	m/z (Calc)	Diff (mDa)	Height	Height %	Height Sum %	m/z	Diff (ppm)
1256377.7	93.4	100	148.9526	0	1273258.4	100	94.6	148.9526	0.13
24944.2	1.9	2	149.9544	-0.1	21644.6	1.7	1.6	149.9545	-0.97
64092.5	4.8	5.1	150.9494	-0.1	50511.4	4	3.8	150.9495	-0.72

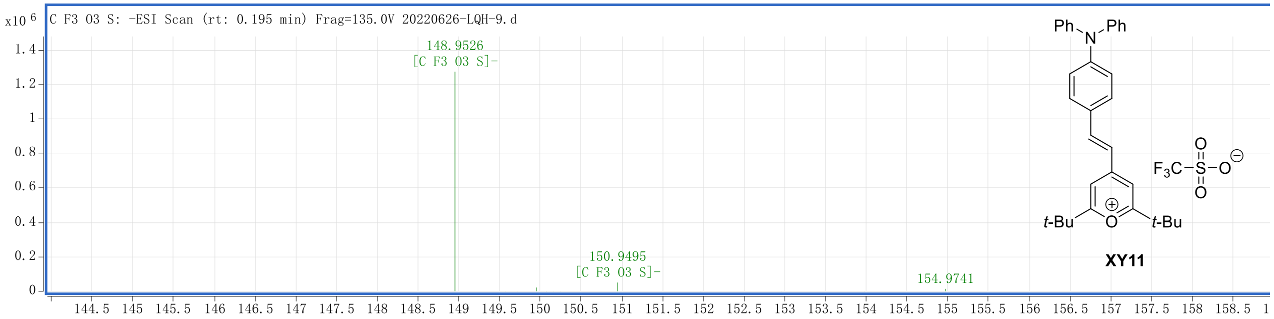

^{1}H NMR Spectrum of XY11 (500 MHz, $CDCl_3$)

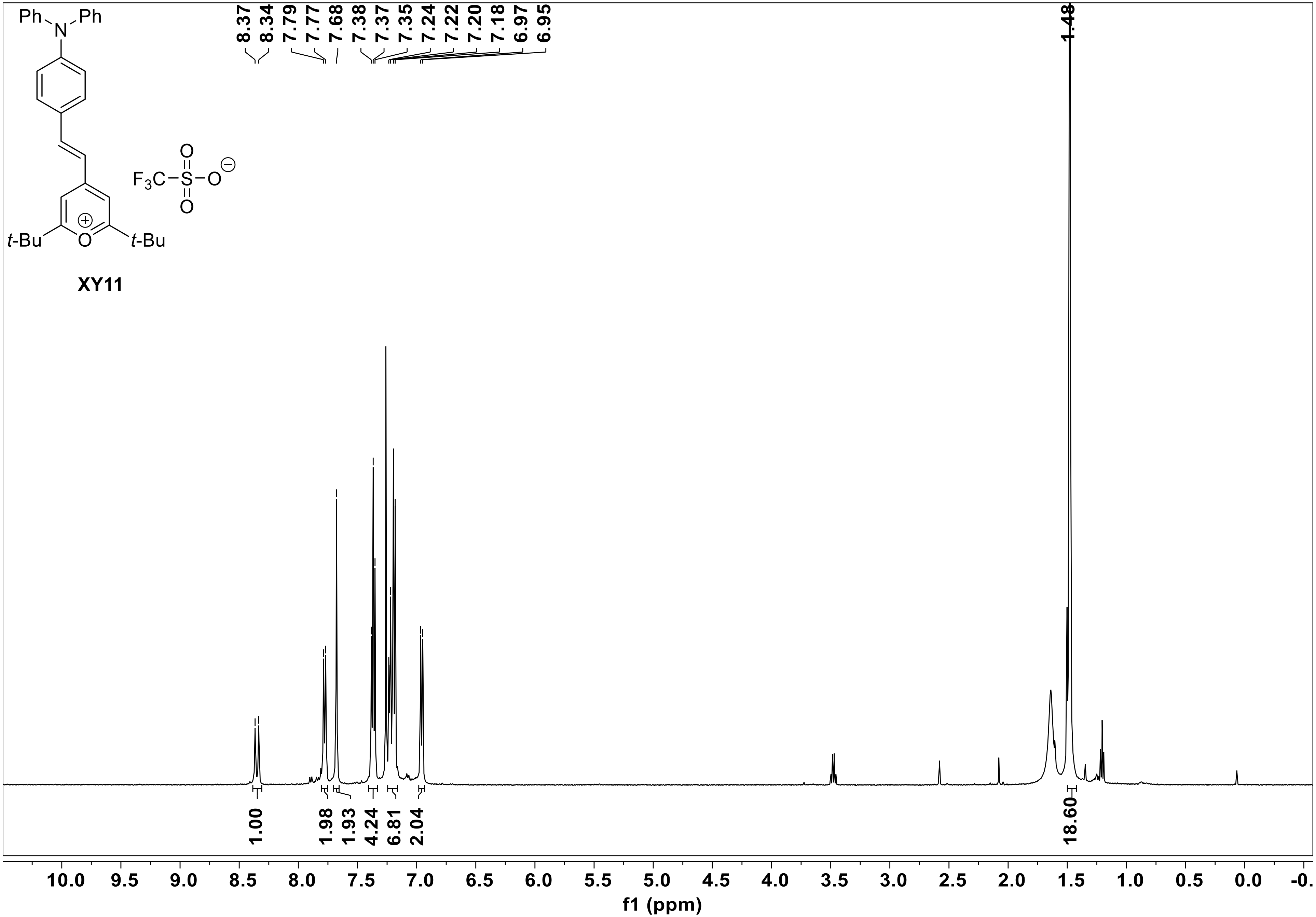

^{13}C NMR Spectrum of XY11 (126 MHz, $CDCl_3$)

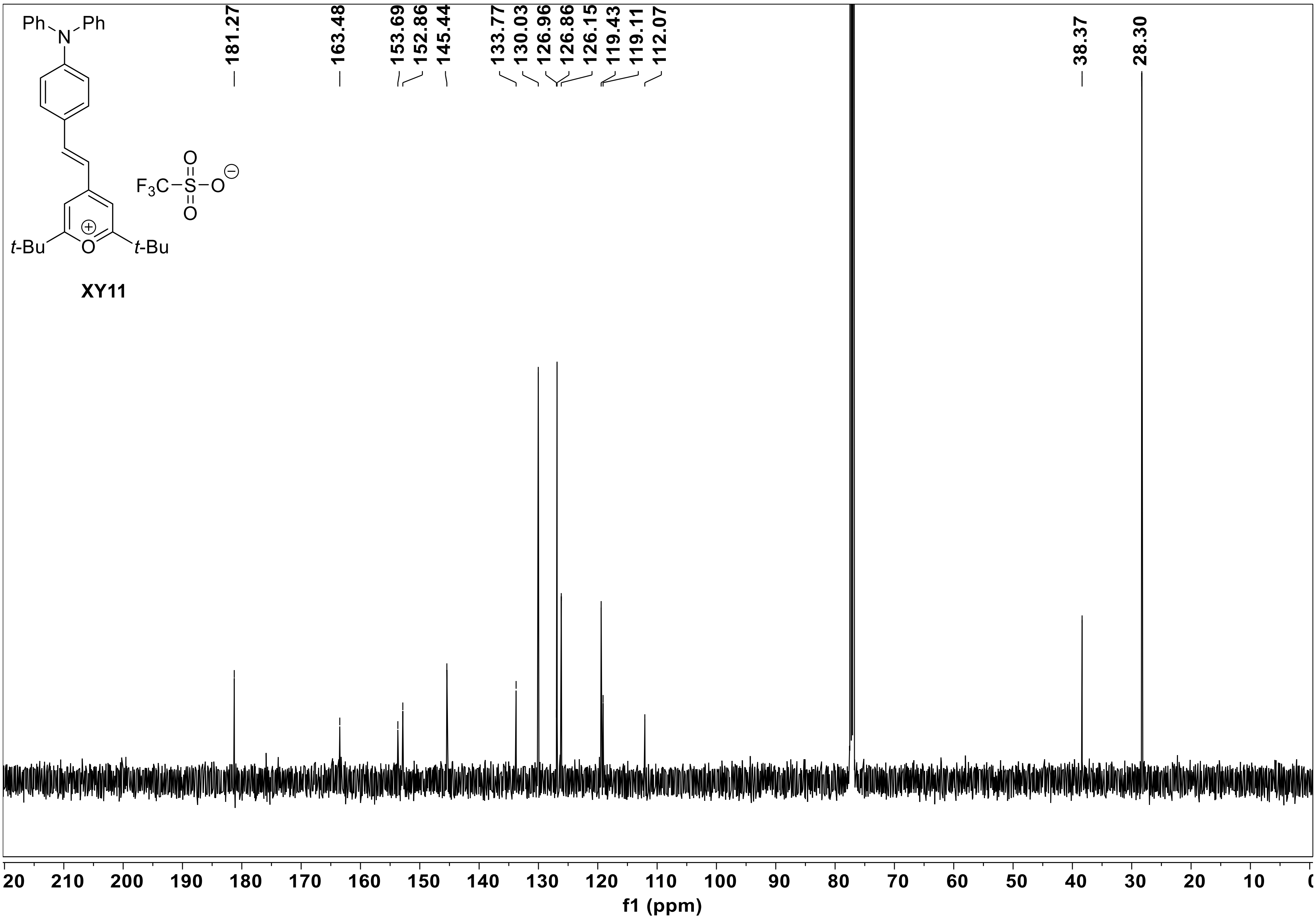

^{19}F NMR Spectrum of XY11 (282 MHz, $CDCl_3$)

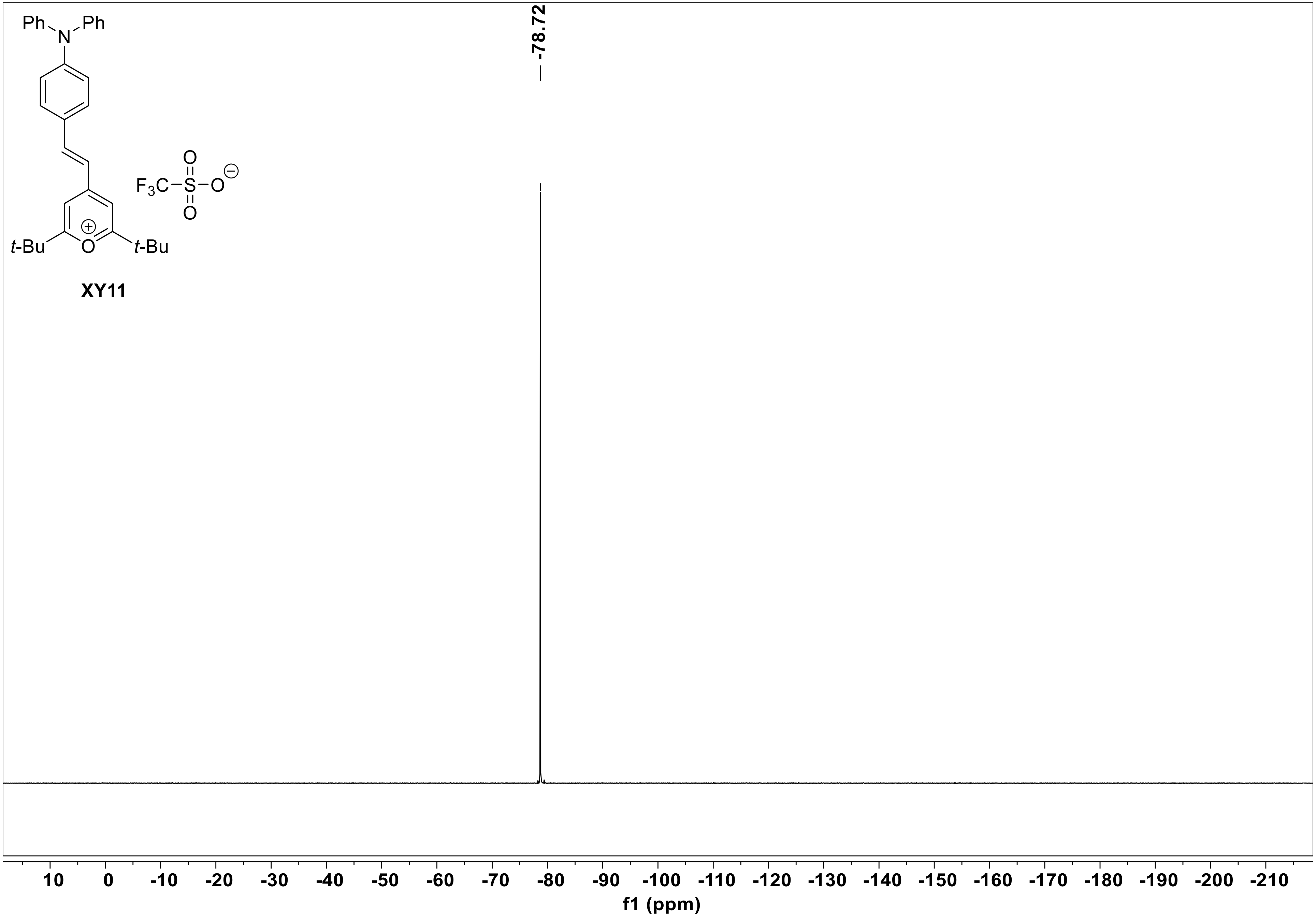

HR-MS Spectra of XY12

Best	ID Source	Name	Formula	Species	m/z	Score	Score (RT)	RT Diff	Diff (ppm)	Score (Lib)	Score (DB)	Score (MFG)
TRUE	MFG		C25 H34 N O	M+	364.2644	83.67			-1.71			83.67

Species	m/z	Score (iso. abund)	Score (mass)	Score (MFG, MS/MS)	Score (MS)	Score (MFG)	Score (iso. spacing)	Height	Ion Formula
M+	364.2644	56.42	97.65		83.67	83.67	88.41	9247484	C25 H34 N O

Height (Calc)	Height Sum% (Calc)	Height % (Calc)	m/z (Calc)	Diff (mDa)	Height	Height %	Height Sum %	m/z	Diff (ppm)
10335128.1	75.7	100	364.2635	-1	9247484	100	67.7	364.2644	-2.61
2876657.8	21.1	27.8	365.2668	0.6	3574874.2	38.7	26.2	365.2662	1.59
406395.4	3	3.9	366.27	-2	735675.4	8	5.4	366.272	-5.36
38929.5	0.3	0.4	367.2731	-3.3	99077.1	1.1	0.7	367.2764	-9.09

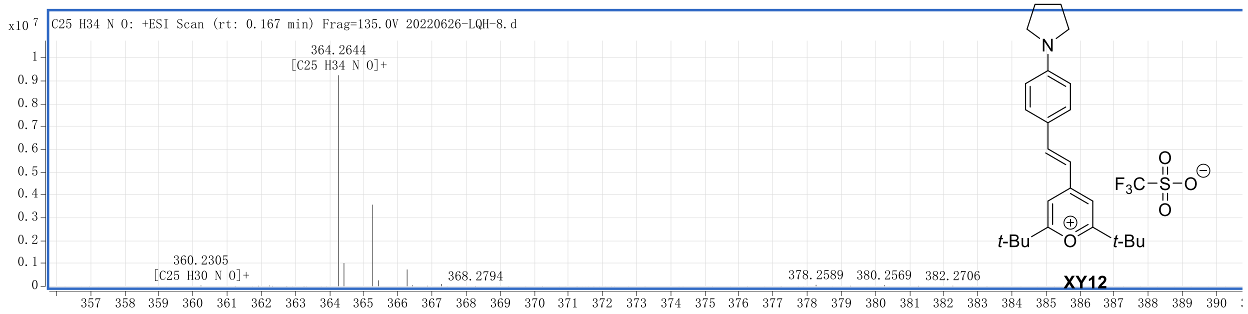

Best	ID Source	Name	Formula	Species	m/z	Score	Score (RT)	RT Diff	Diff (ppm)	Score (Lib)	Score (DB)	Score (MFG)
TRUE	MFG		C F3 O3 S	M-	148.9530	98.67			-2.71			98.67

Species	m/z	Score (iso. abund)	Score (mass)	Score (MFG, MS/MS)	Score (MS)	Score (MFG)	Score (iso. spacing)	Height	Ion Formula
M-	148.953	98.78	97.99		98.67	98.67	99.89	1875227	C F3 O3 S

Height (Calc)	Height Sum% (Calc)	Height % (Calc)	m/z (Calc)	Diff (mDa)	Height	Height %	Height Sum %	m/z	Diff (ppm)
1856708.9	93.4	100	148.9526	-0.4	1875227	100	94.3	148.953	-2.76
36863.2	1.9	2	149.9544	0.1	34732.7	1.9	1.7	149.9543	0.91
94717.7	4.8	5.1	150.9494	-0.5	78330.1	4.2	3.9	150.9499	-3.11

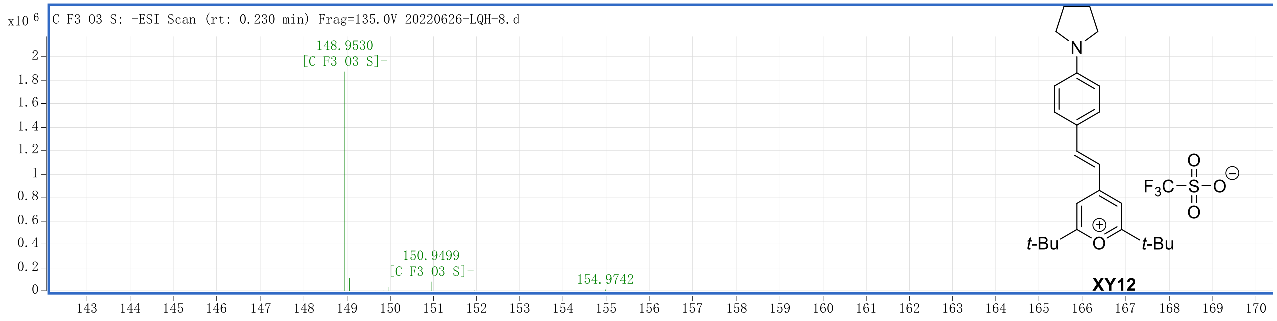

^{1}H NMR Spectrum of XY12 (500 MHz, $CDCl_3$)

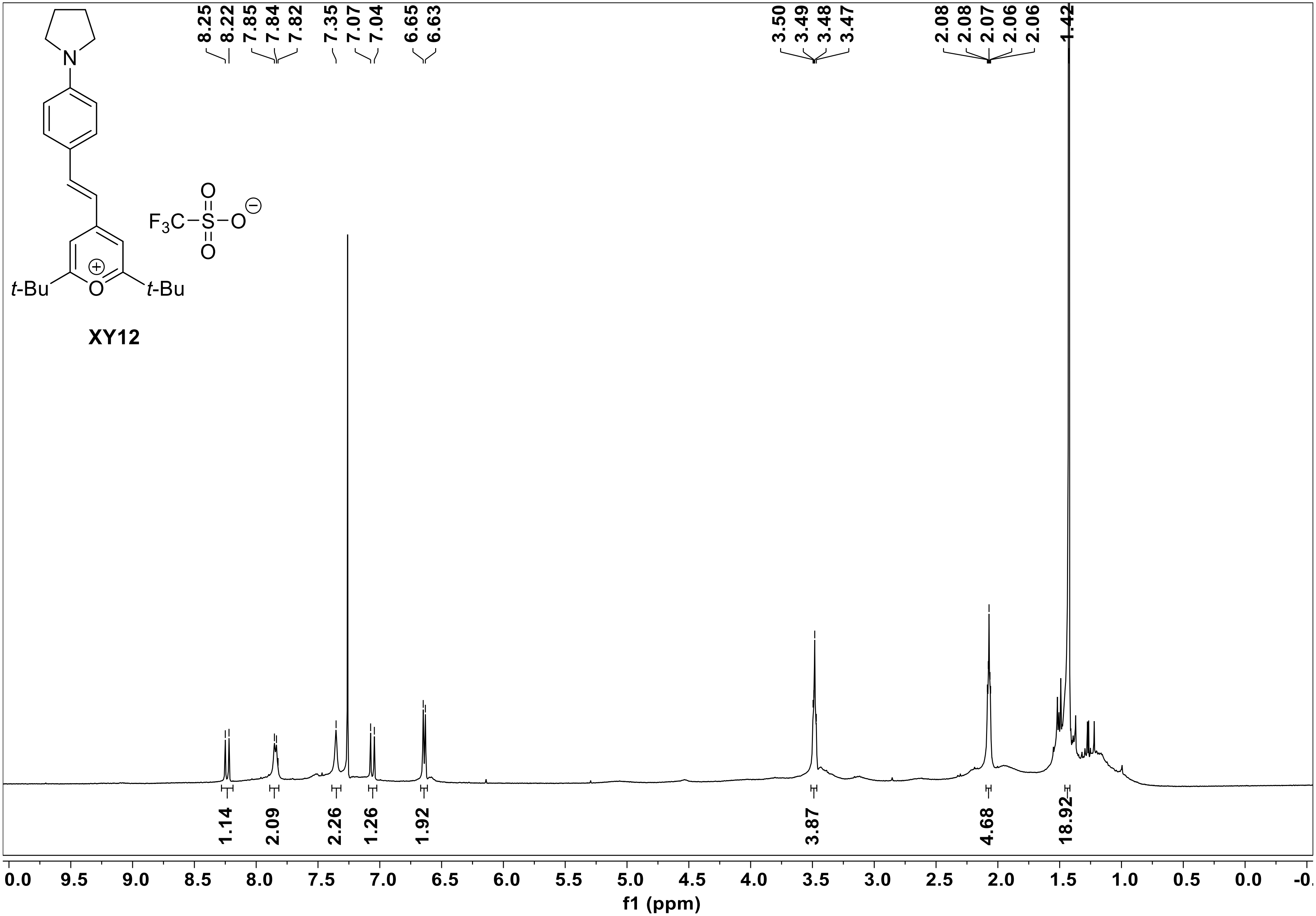

^{13}C NMR Spectrum of XY14 (126 MHz, $CDCl_3$)

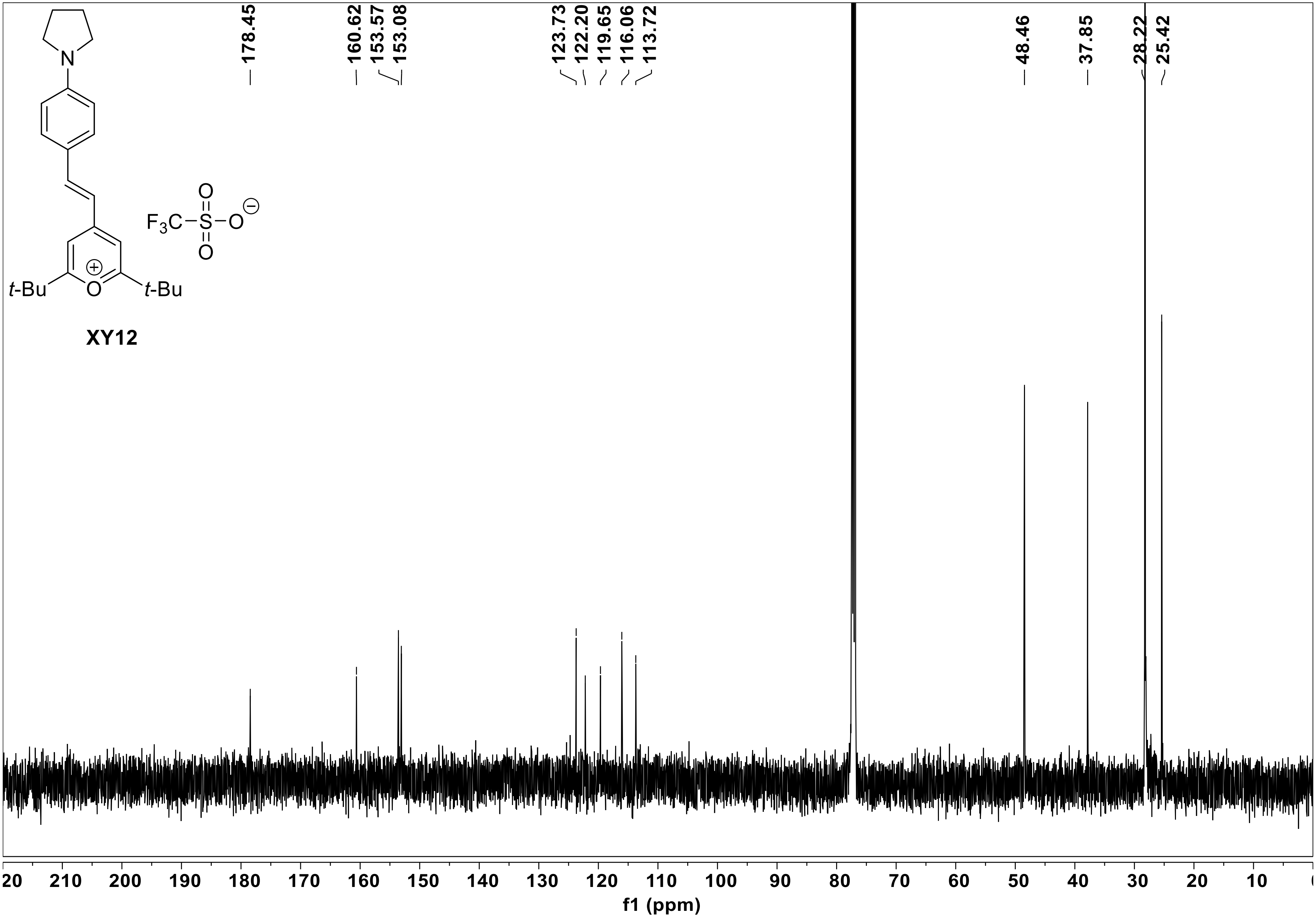

^{19}F NMR Spectrum of XY12 (282 MHz, $CDCl_3$+TFA)

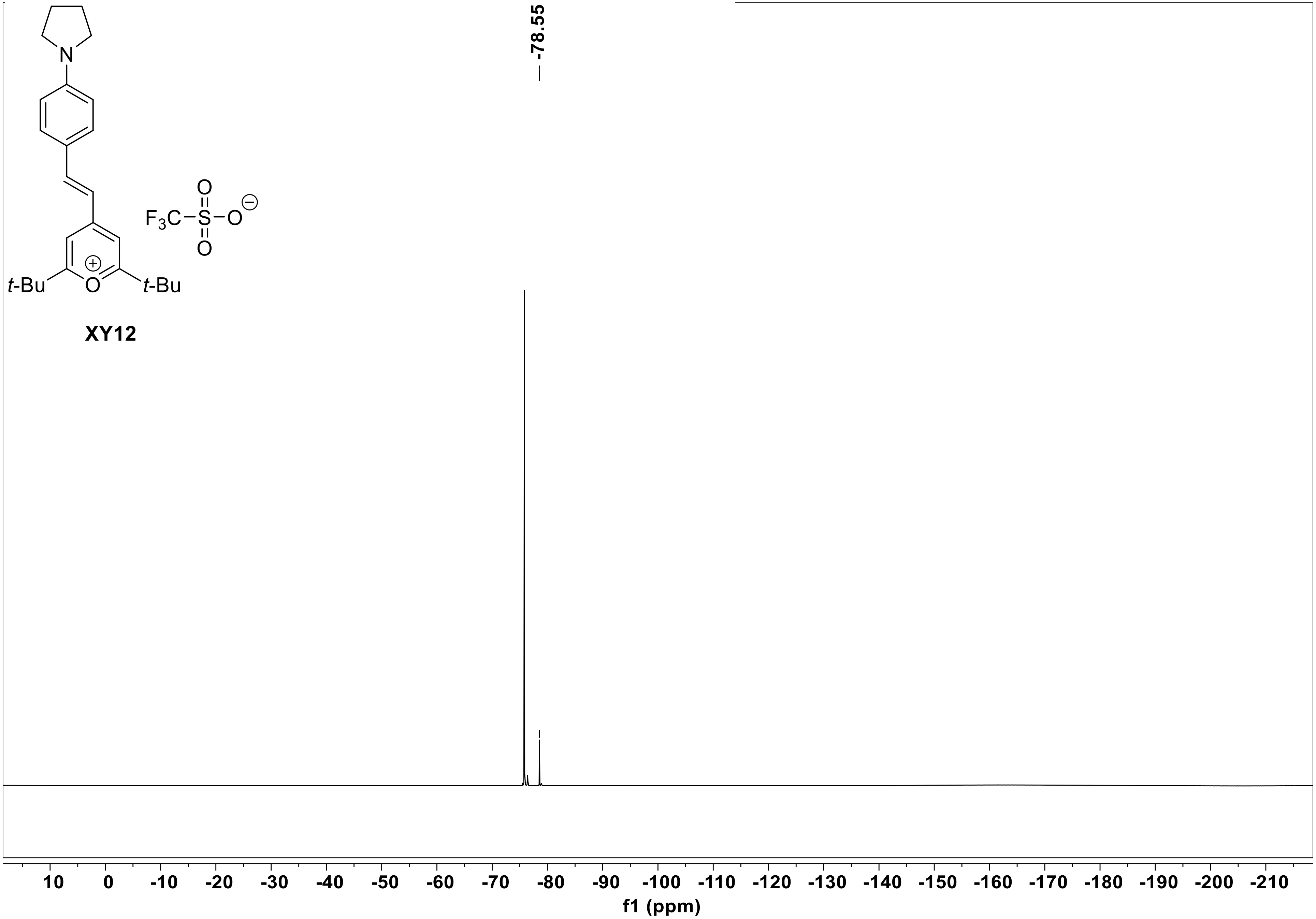

HR-MS Spectra of XY13

Best	ID Source	Name	Formula	Species	m/z	Score	Score (RT)	RT Diff	Diff (ppm)	Score (Lib)	Score (DB)	Score (MFG)
TRUE	MFG		C26 H36 N O	M+	378.2792	90.73			0.85			90.73

Species	m/z	Score (iso. abund)	Score (mass)	Score (MFG, MS/MS)	Score (MS)	Score (MFG)	Score (iso. spacing)	Height	Ion Formula
M+	378.2792	74.18	99.39		90.73	90.73	93.26	6700624.5	C26 H36 N O

Height (Calc)	Height Sum% (Calc)	Height % (Calc)	m/z (Calc)	Diff (mDa)	Height	Height %	Height Sum %	m/z	Diff (ppm)
7149057.2	75.1	100	378.2791	0	6700624.5	100	70.4	378.2792	-0.04
2068820.3	21.7	28.9	379.2825	1.1	2444286.5	36.5	25.7	379.2814	2.82
303110.9	3.2	4.2	380.2857	1.5	376077.3	5.6	3.9	380.2842	3.88

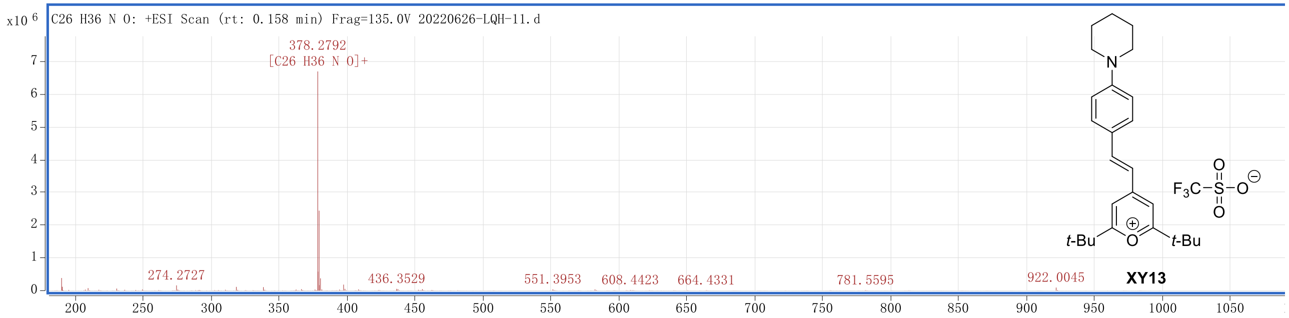

Best	ID Source	Name	Formula	Species	m/z	Score	Score (RT)	RT Diff	Diff (ppm)	Score (Lib)	Score (DB)	Score (MFG)
TRUE	MFG		C F3 O3 S	M-	148.9529	99.22			-2.27			99.22

Species	m/z	Score (iso. abund)	Score (mass)	Score (MFG, MS/MS)	Score (MS)	Score (MFG)	Score (iso. spacing)	Height	Ion Formula
M-	148.9529	99.79	98.58		99.22	99.22	99.8	3160977	C F3 O3 S

Height (Calc)	Height Sum% (Calc)	Height % (Calc)	m/z (Calc)	Diff (mDa)	Height	Height %	Height Sum %	m/z	Diff (ppm)
3150113.7	93.4	100	148.9526	-0.4	3160977	100	93.7	148.9529	-2.43
62542.6	1.9	2	149.9544	0	63125.7	2	1.9	149.9544	-0.31
160699.1	4.8	5.1	150.9494	0	149252.8	4.7	4.4	150.9494	0.22

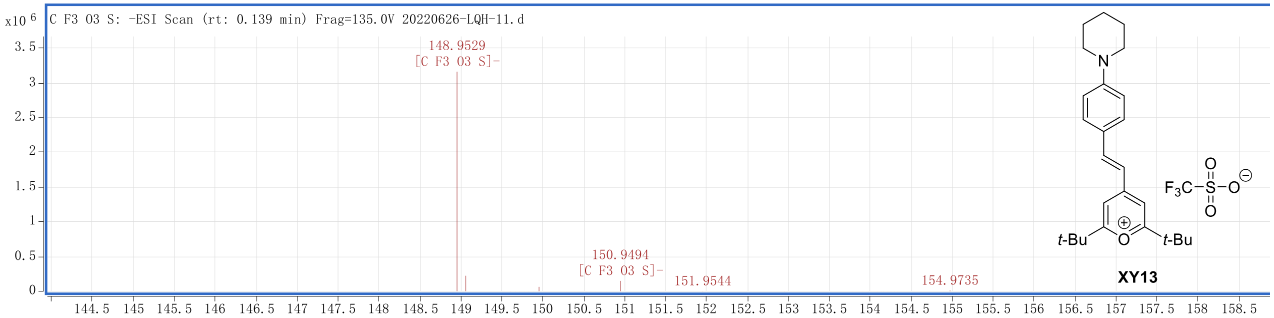

^{1}H NMR Spectrum of XY13 (500 MHz, $CDCl_3$)

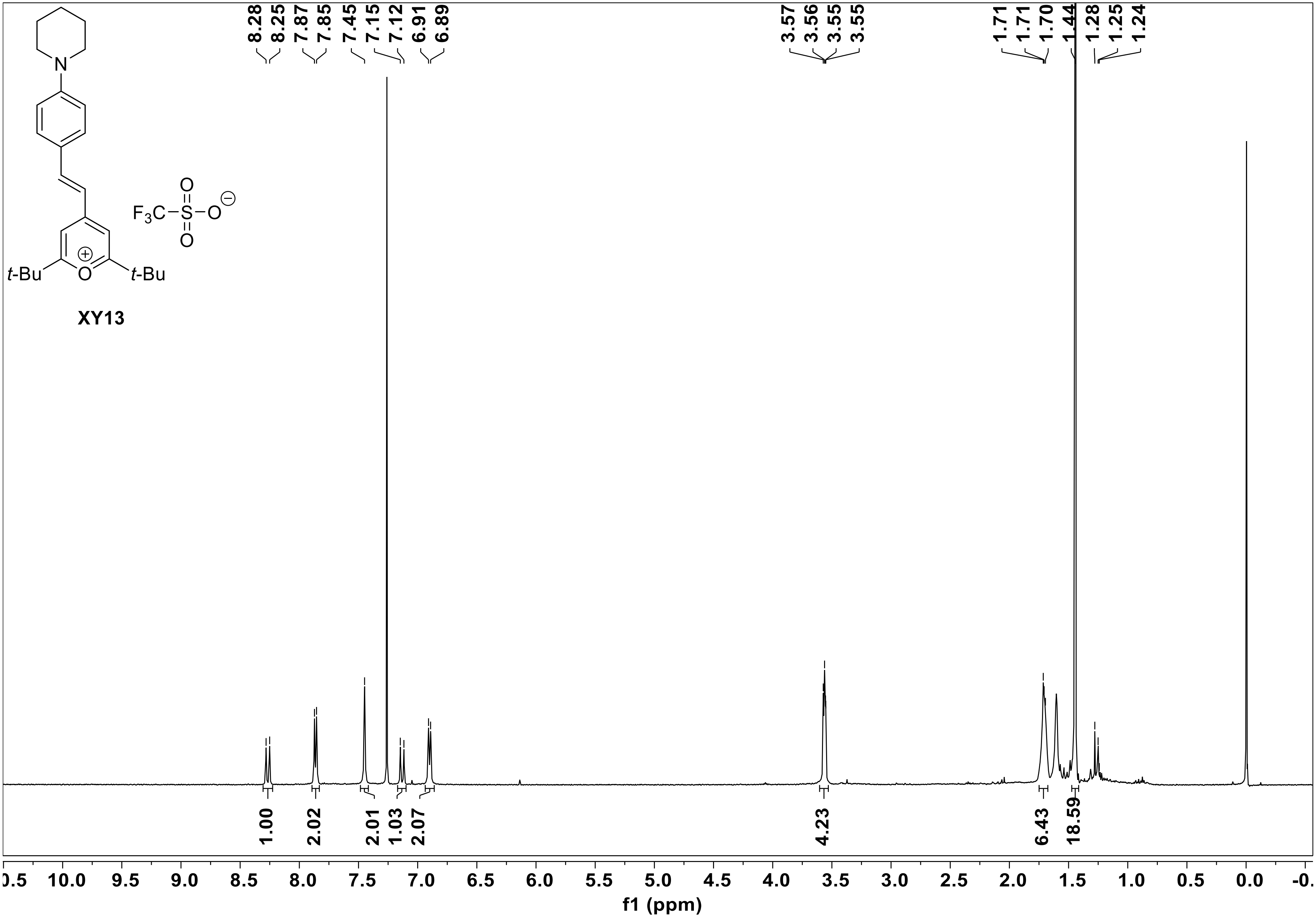

^{13}C NMR Spectrum of XY13 (126 MHz, $CDCl_3$+TFA)

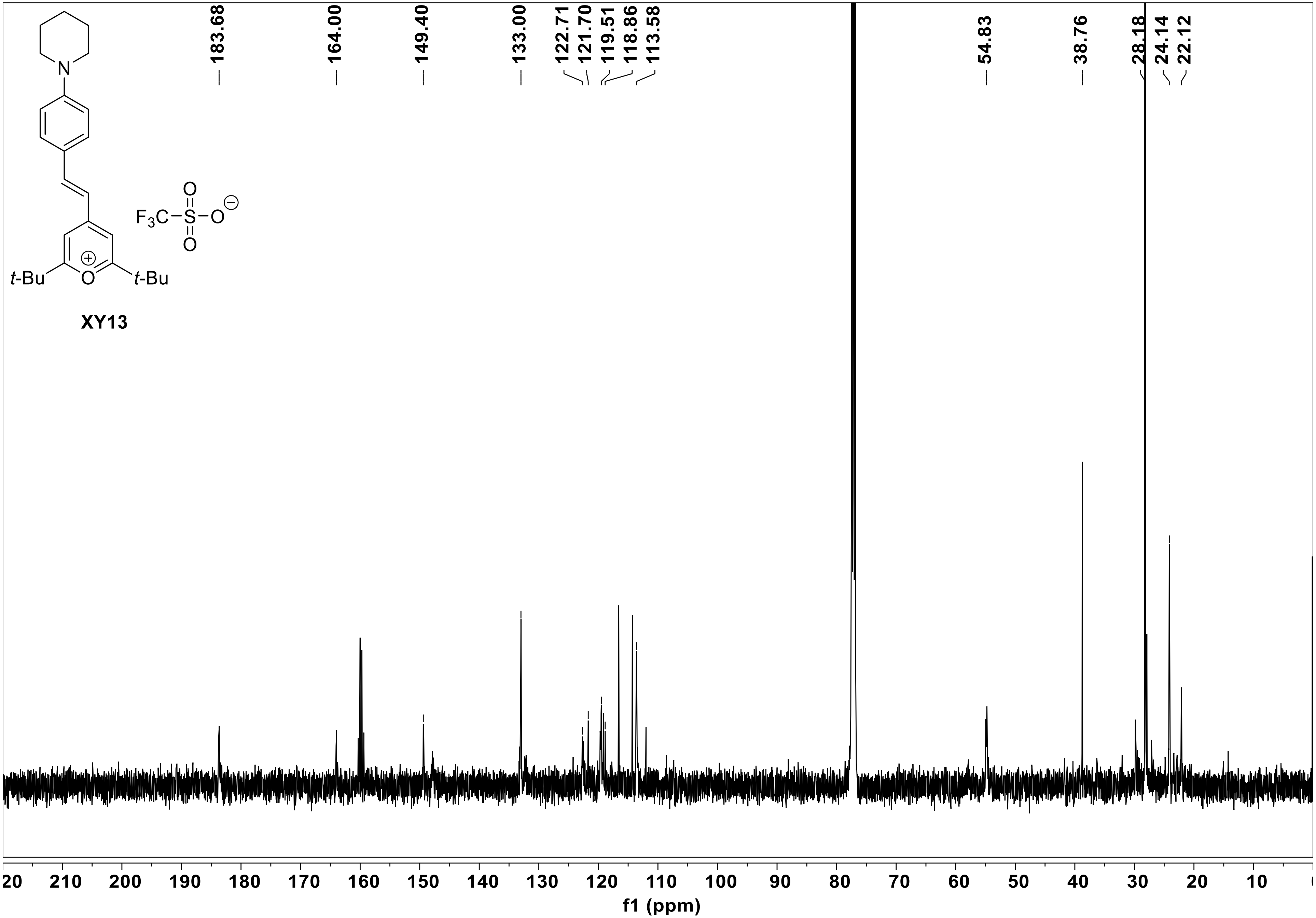

^{19}F NMR Spectrum of XY13 (282 MHz, $CDCl_3$)

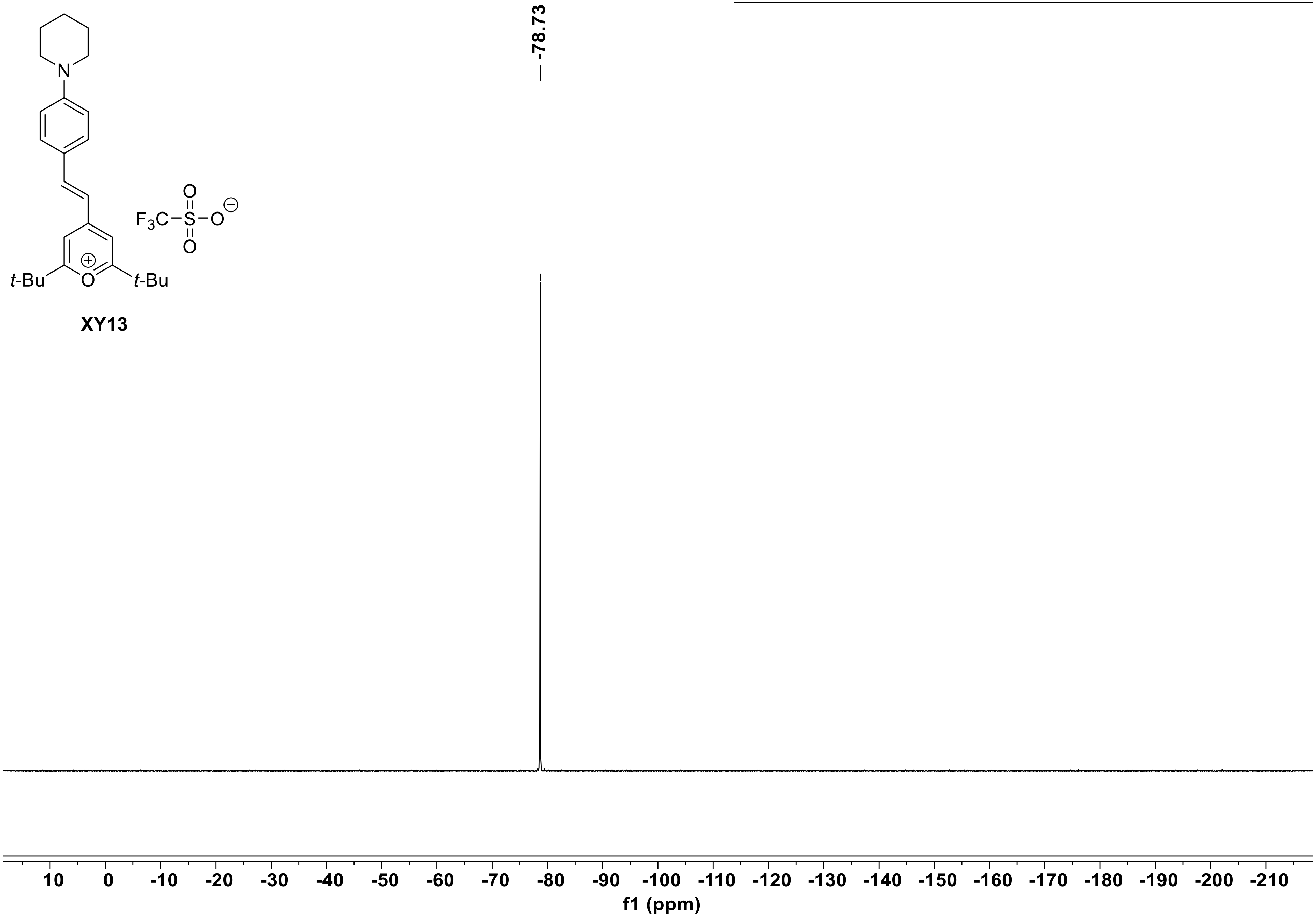

HR-MS Spectra of XY14

Best	ID Source	Name	Formula	Species	m/z	Score	Score (RT)	RT Diff	Diff (ppm)	Score (Lib)	Score (DB)	Score (MFG)
TRUE	MFG		C24 H32 N O	M+	350.2484	81.43			0.01			81.43

Species	m/z	Score (iso. abund)	Score (mass)	Score (MFG, MS/MS)	Score (MS)	Score (MFG)	Score (iso. spacing)	Height	Ion Formula
M+	350.2484	49.48	100		81.43	81.43	82.62	10068983	C24 H32 N O

Height (Calc)	Height Sum% (Calc)	Height % (Calc)	m/z (Calc)	Diff (mDa)	Height	Height %	Height Sum %	m/z	Diff (ppm)
11154655.9	76.7	100	350.2478	-0.6	10068983	100	69.2	350.2484	-1.58
2981551.5	20.5	26.7	351.2512	1.2	3865095.8	38.4	26.6	351.25	3.3
405659.3	2.8	3.6	352.2543	1.9	607788	6	4.2	352.2524	5.37

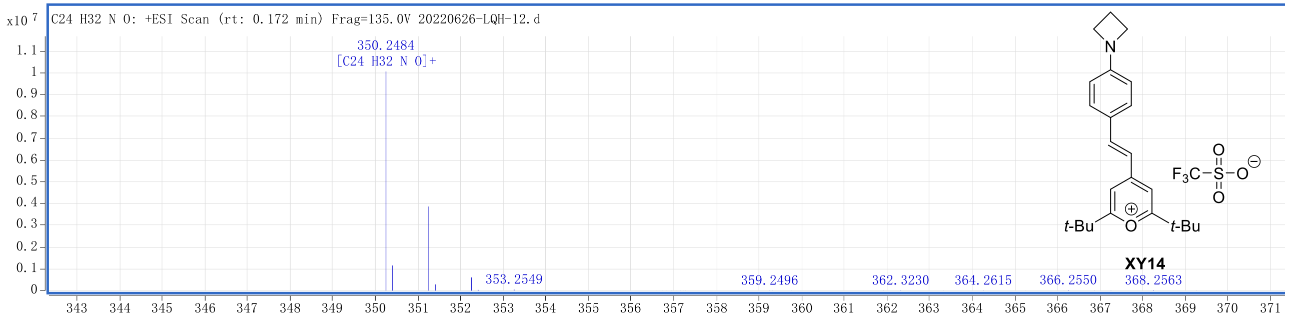

Best	ID Source	Name	Formula	Species	m/z	Score	Score (RT)	RT Diff	Diff (ppm)	Score (Lib)	Score (DB)	Score (MFG)
TRUE	MFG		C F3 O3 S	M-	148.9530	98.55			-2.86			98.55

Species	m/z	Score (iso. abund)	Score (mass)	Score (MFG, MS/MS)	Score (MS)	Score (MFG)	Score (iso. spacing)	Height	Ion Formula
M-	148.953	98.8	97.77		98.55	98.55	99.8	2098132.2	C F3 O3 S

Height (Calc)	Height Sum% (Calc)	Height % (Calc)	m/z (Calc)	Diff (mDa)	Height	Height %	Height Sum %	m/z	Diff (ppm)
2078829	93.4	100	148.9526	-0.4	2098132.2	100	94.2	148.953	-3.02
41273.2	1.9	2	149.9544	0	40270.1	1.9	1.8	149.9544	0.09
106048.9	4.8	5.1	150.9494	-0.1	87748.7	4.2	3.9	150.9495	-0.34

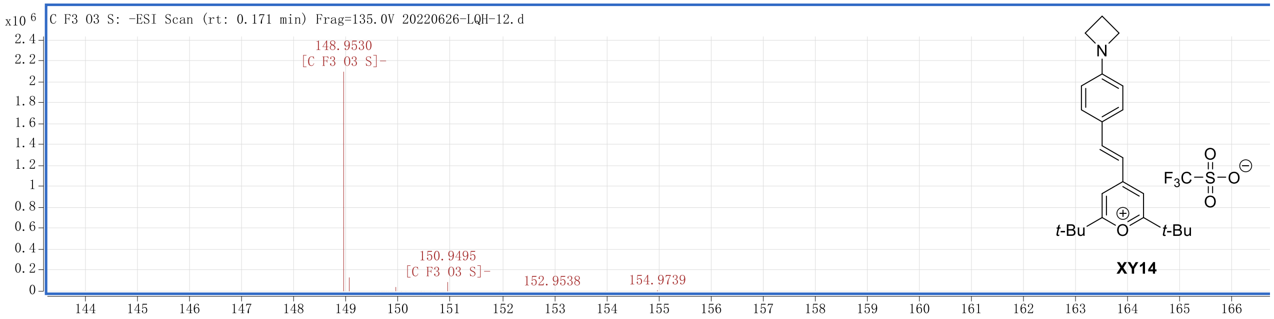

^{1}H NMR Spectrum of XY14 (500 MHz, $CDCl_3$)

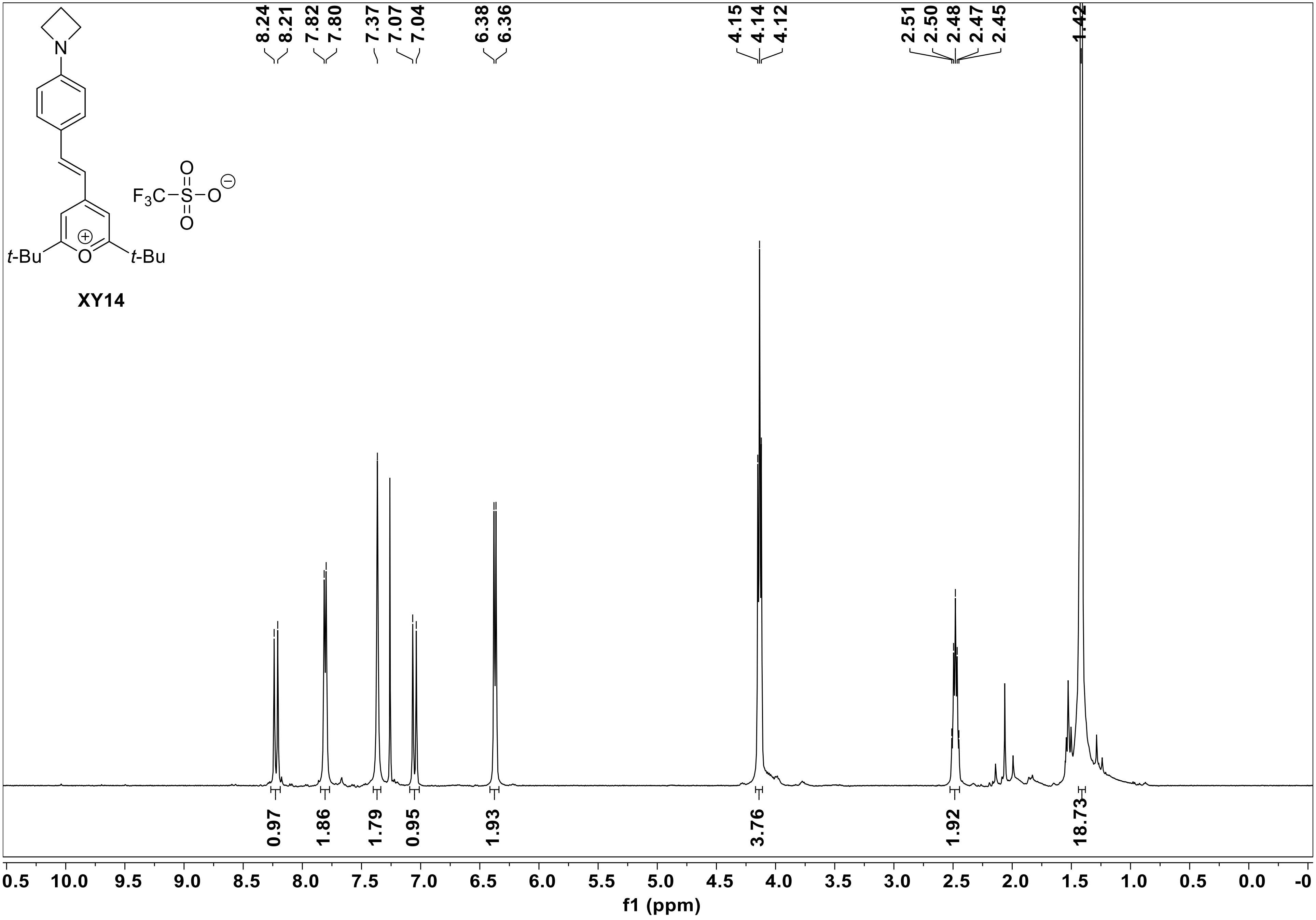

^{13}C NMR Spectrum of XY14 (126 MHz, $CDCl_3$)

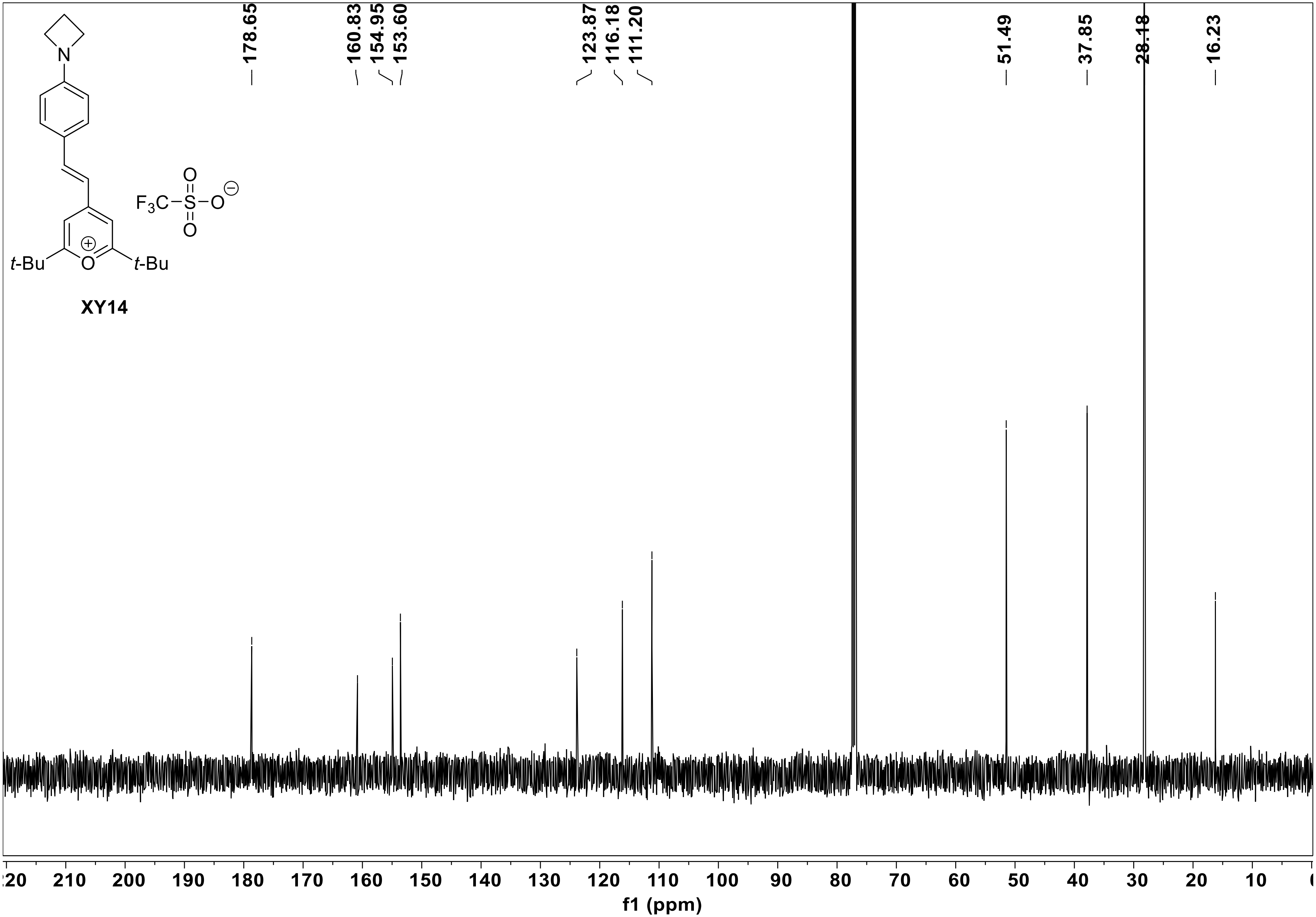

^{19}F NMR Spectrum of XY14 (282 MHz, $CDCl_3$)

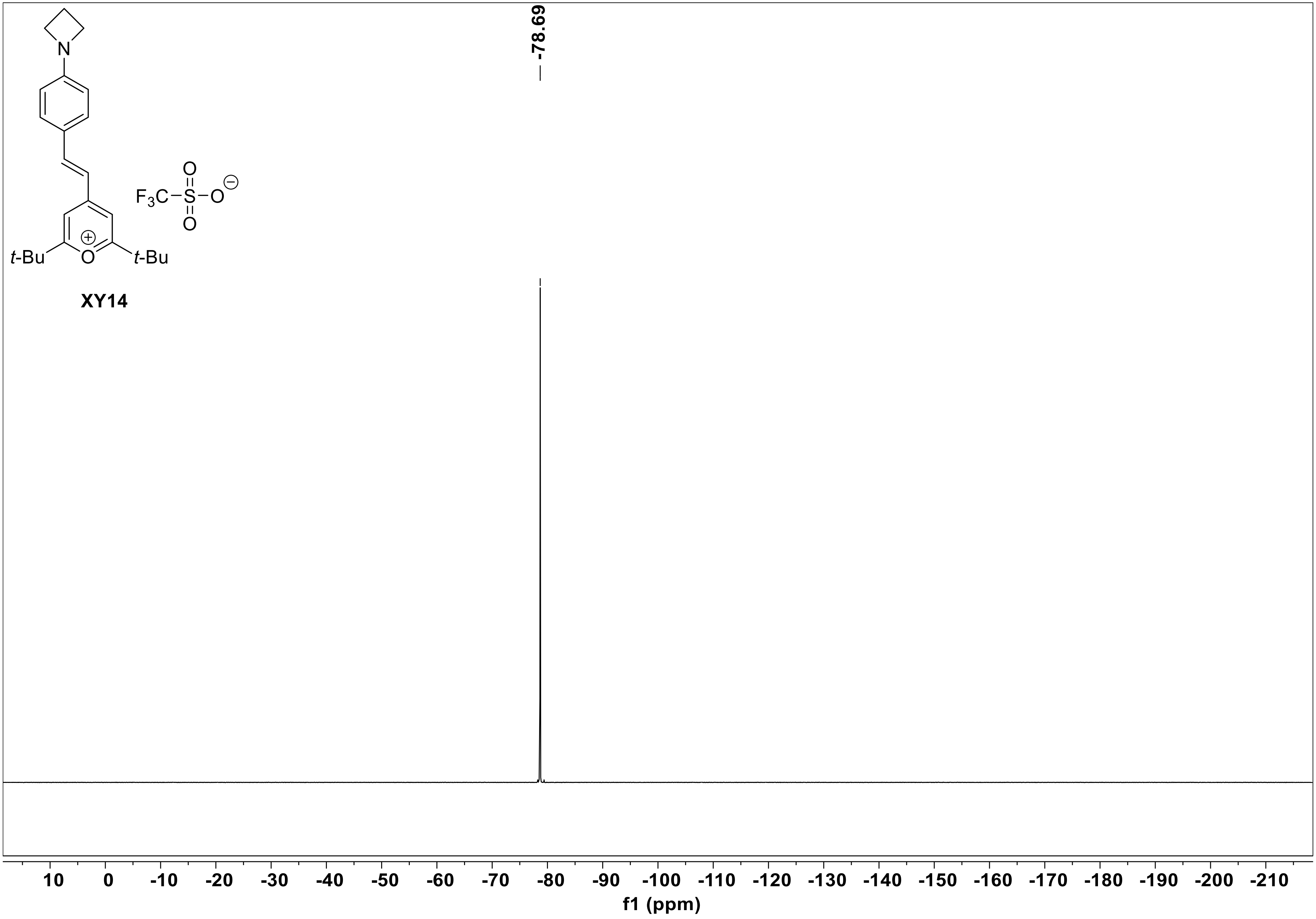

HR-MS Spectra of XY15

Best	ID Source	Name	Formula	Species	m/z	Score	Score (RT)	RT Diff	Diff (ppm)	Score (Lib)	Score (DB)	Score (MFG)
TRUE	MFG		C25 H34 N O2	M+	380.2589	87.66			-0.32			87.66

Species	m/z	Score (iso. abund)	Score (mass)	Score (MFG, MS/MS)	Score (MS)	Score (MFG)	Score (iso. spacing)	Height	Ion Formula
M+	380.2589	64.5	99.91		87.66	87.66	90.95	8297659.5	C25 H34 N O2

Height (Calc)	Height Sum% (Calc)	Height % (Calc)	m/z (Calc)	Diff (mDa)	Height	Height %	Height Sum %	m/z	Diff (ppm)
8987906.4	75.7	100	380.2584	-0.5	8297659.5	100	69.9	380.2589	-1.37
2505098.8	21.1	27.9	381.2617	0.7	3071386.2	37	25.9	381.261	1.95
372843.3	3.1	4.1	382.2648	1.2	496802.8	6	4.2	382.2636	3.05

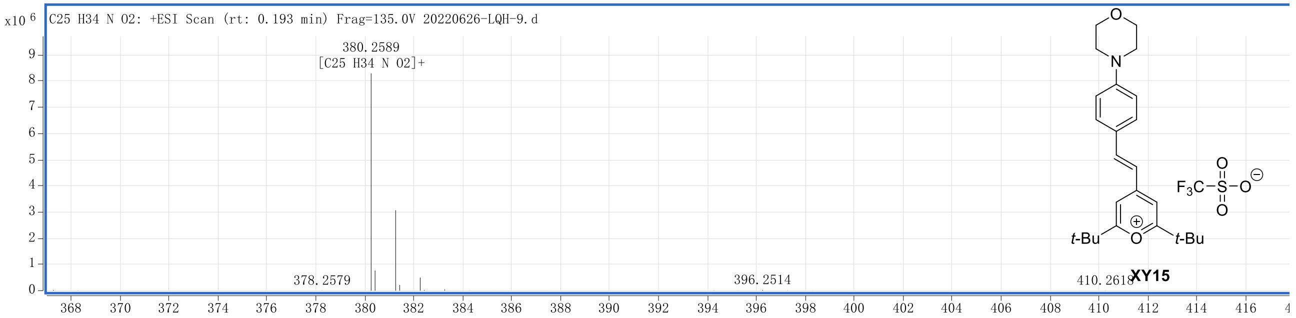

Best	ID Source	Name	Formula	Species	m/z	Score	Score (RT)	RT Diff	Diff (ppm)	Score (Lib)	Score (DB)	Score (MFG)
TRUE	MFG		C F3 O3 S	M-	148.9525	99.21			0.57			99.21

Species	m/z	Score (iso. abund)	Score (mass)	Score (MFG, MS/MS)	Score (MS)	Score (MFG)	Score (iso. spacing)	Height	Ion Formula
M-	148.9525	97.52	99.91		99.21	99.21	99.85	1144940.6	C F3 O3 S

Height (Calc)	Height Sum% (Calc)	Height % (Calc)	m/z (Calc)	Diff (mDa)	Height	Height %	Height Sum %	m/z	Diff (ppm)
1127288.6	93.4	100	148.9526	0.1	1144940.6	100	94.8	148.9525	0.7
22381.3	1.9	2	149.9544	-0.5	18565	1.6	1.5	149.9549	-3.35
57507.2	4.8	5.1	150.9494	-0.2	43671.6	3.8	3.6	150.9496	-1.14

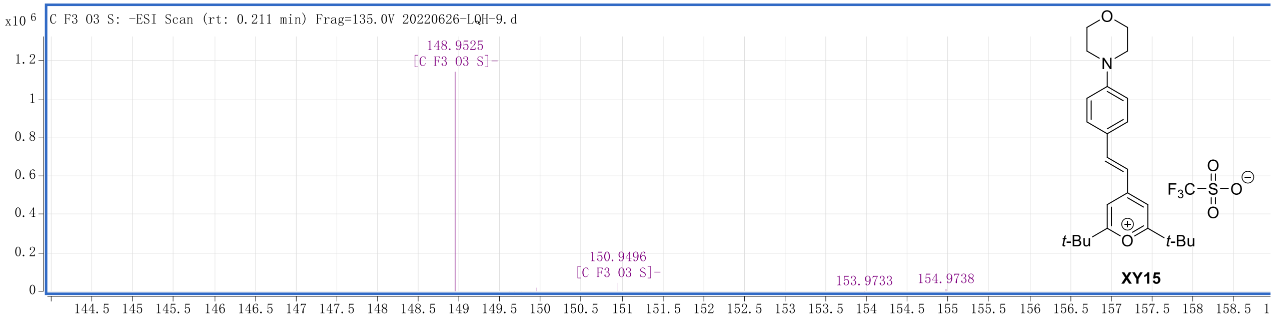

^{1}H NMR Spectrum of XY15 (500 MHz, $CDCl_3$)

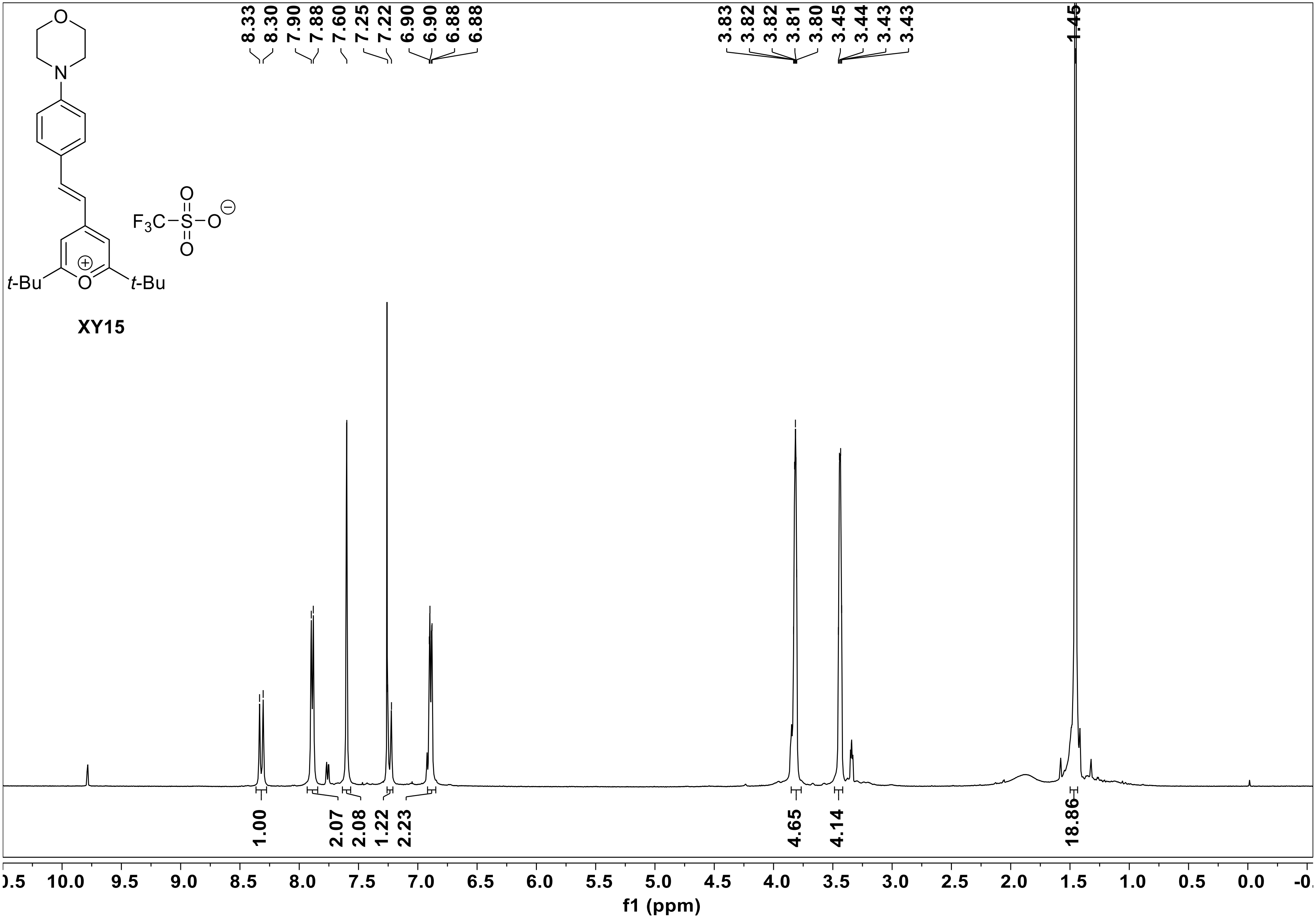

^{13}C NMR Spectrum of XY15 (126 MHz, $CDCl_3$)

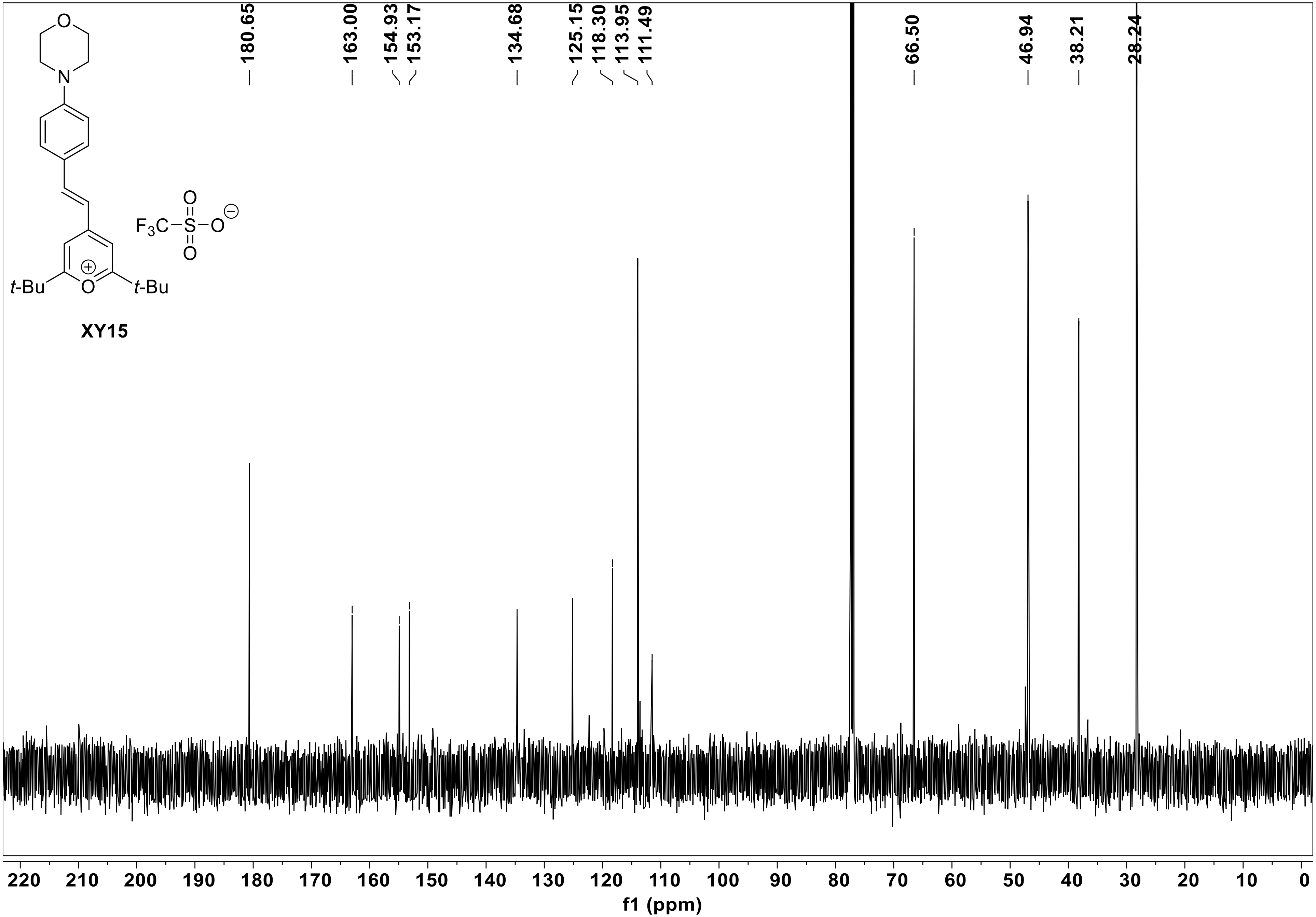

^{19}F NMR Spectrum of XY15 (282 MHz, $CDCl_3$)

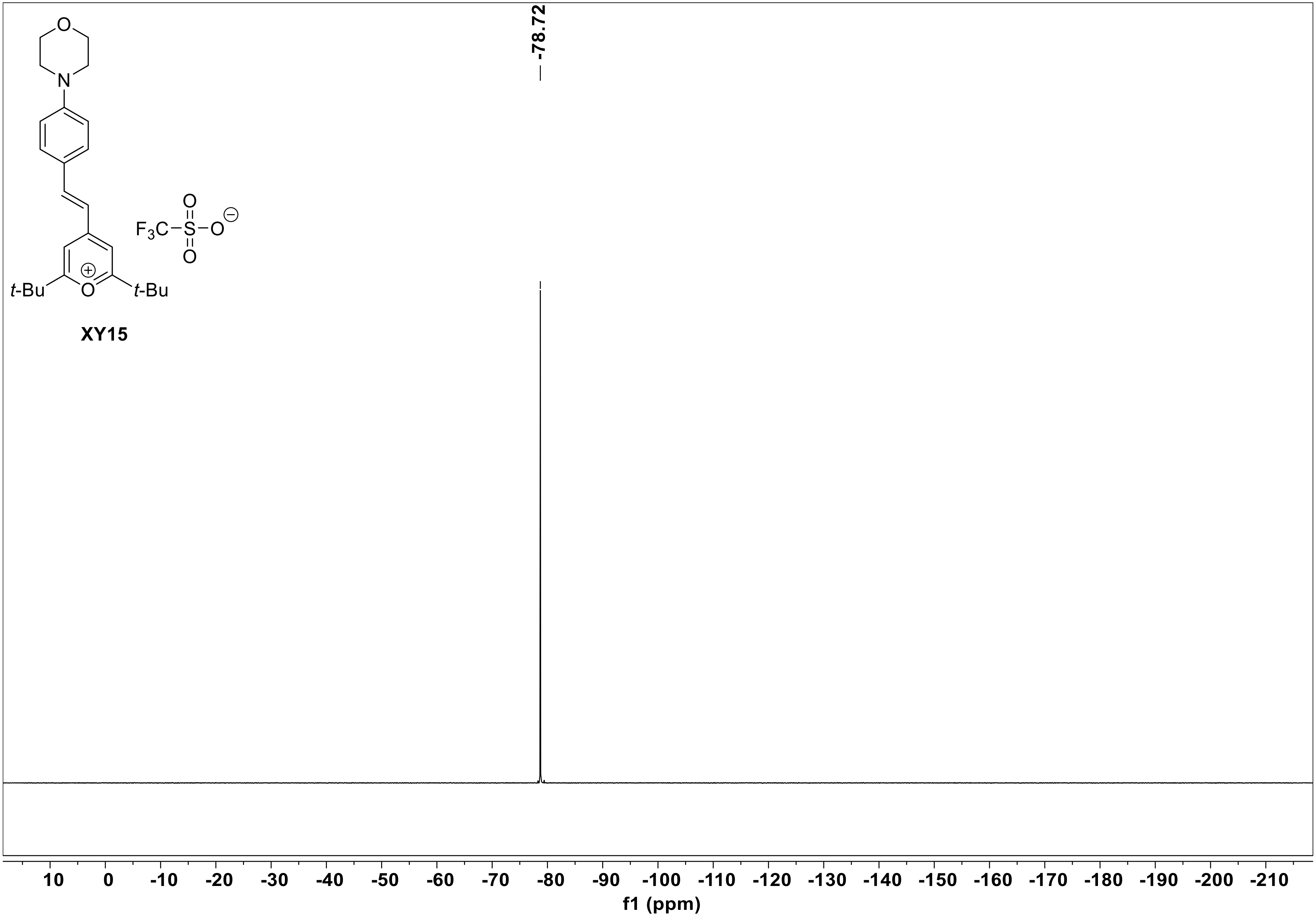

HR-MS Spectra of XY16

Best	ID Source	Name	Formula	Species	m/z	Score	Score (RT)	RT Diff	Diff (ppm)	Score (Lib)	Score (DB)	Score (MFG)
TRUE	MFG		C25 H33 F N O	M+	382.2544	87.38			0.28			87.38

Species	m/z	Score (iso. abund)	Score (mass)	Score (MFG, MS/MS)	Score (MS)	Score (MFG)	Score (iso. spacing)	Height	Ion Formula
M+	382.2544	63.06	99.93		87.38	87.38	91.44	8522137	C25 H33 F N O

Height (Calc)	Height Sum% (Calc)	Height % (Calc)	m/z (Calc)	Diff (mDa)	Height	Height %	Height Sum %	m/z	Diff (ppm)
9244886.2	75.9	100	382.2541	-0.3	8522137	100	70	382.2544	-0.75
2572138.9	21.1	27.8	383.2574	1	3171556	37.2	26	383.2564	2.59
363229.4	3	3.9	384.2606	1.2	486561.5	5.7	4	384.2594	3.08

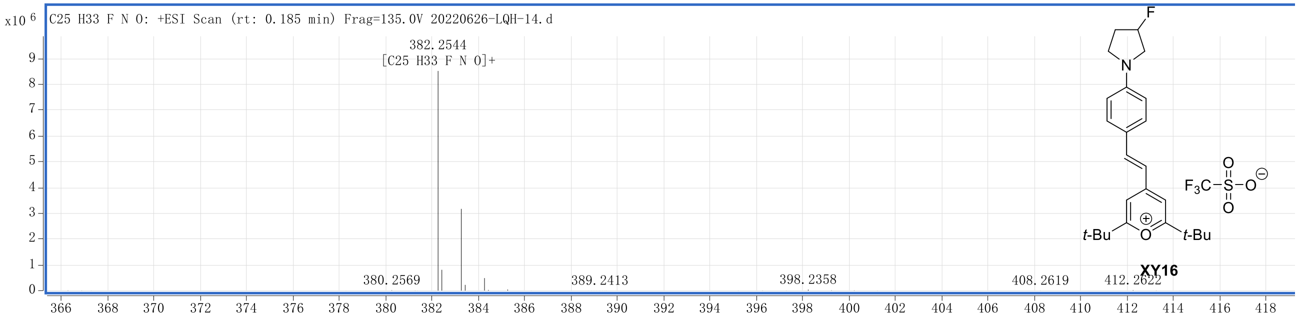

Best	ID Source	Name	Formula	Species	m/z	Score	Score (RT)	RT Diff	Diff (ppm)	Score (Lib)	Score (DB)	Score (MFG)
TRUE	MFG		C F3 O3 S	M-	148.9523	98.9			1.58			98.9

Species	m/z	Score (iso. abund)	Score (mass)	Score (MFG, MS/MS)	Score (MS)	Score (MFG)	Score (iso. spacing)	Height	Ion Formula
M-	148.9523	97.41	99.31		98.9	98.9	99.86	1131819.9	C F3 O3 S

Height (Calc)	Height Sum% (Calc)	Height % (Calc)	m/z (Calc)	Diff (mDa)	Height	Height %	Height Sum %	m/z	Diff (ppm)
1114804	93.4	100	148.9526	0.3	1131819.9	100	94.8	148.9523	1.7
22133.4	1.9	2	149.9544	-0.3	19390.8	1.7	1.6	149.9547	-2.16
56870.3	4.8	5.1	150.9494	0	42597.1	3.8	3.6	150.9494	0.01

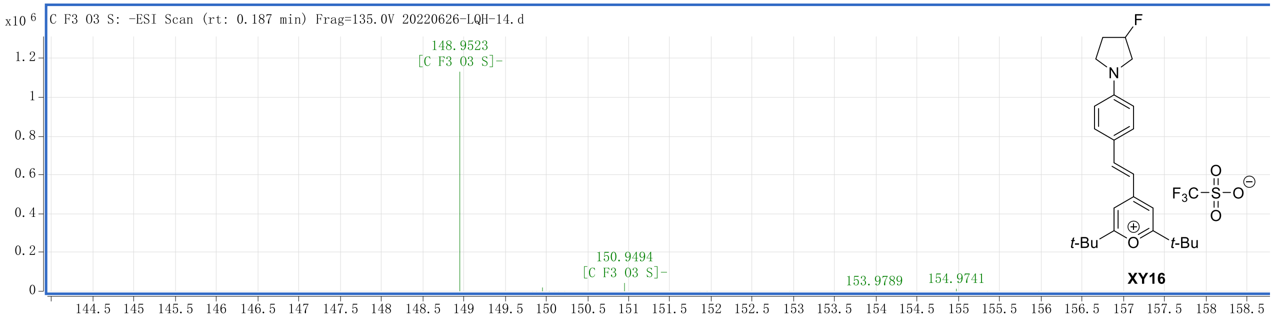

^{1}H NMR Spectrum of XY16 (500 MHz, $CDCl_3$)

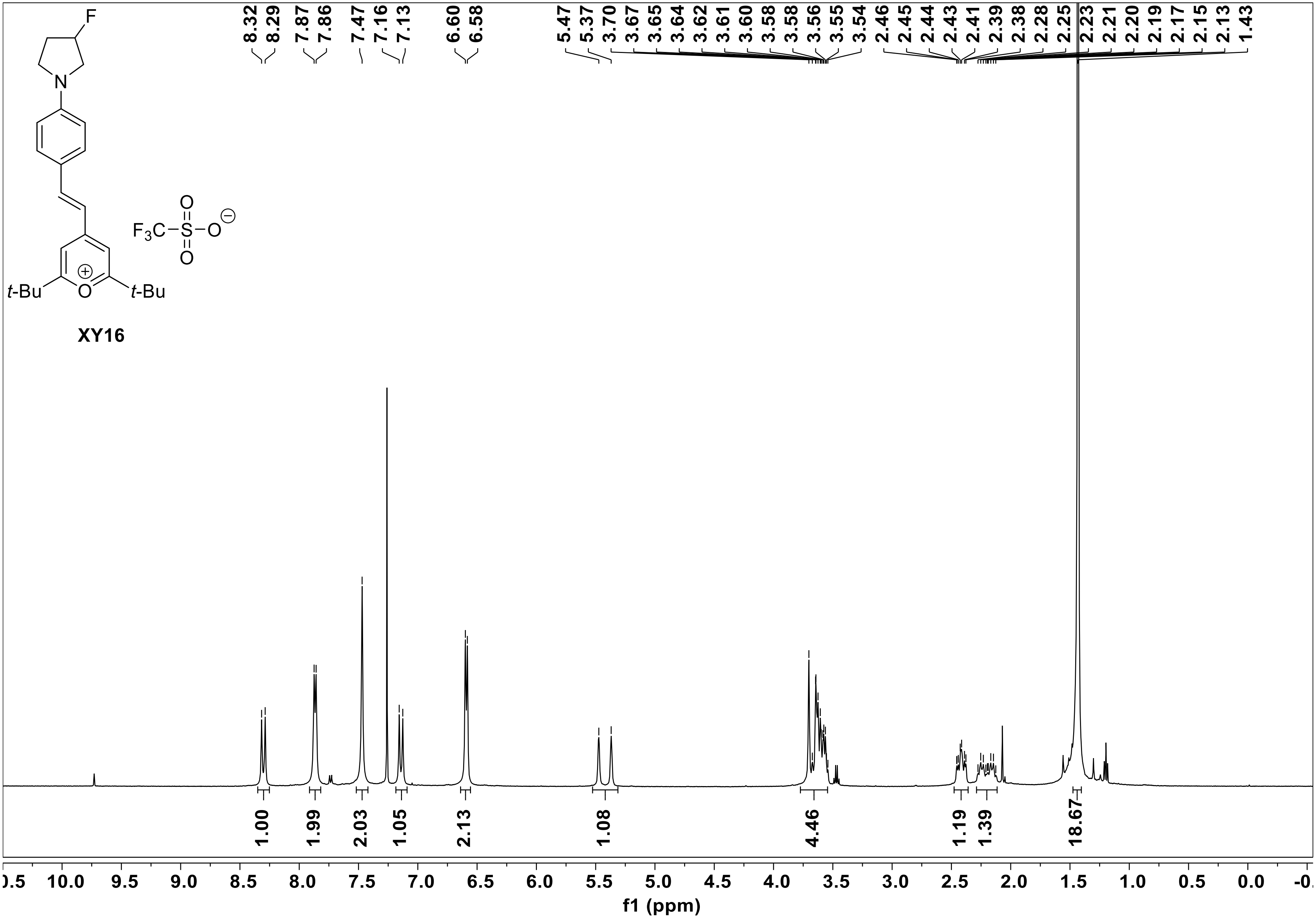

^{13}C NMR Spectrum of XY16 (126 MHz, $CDCl_3$)

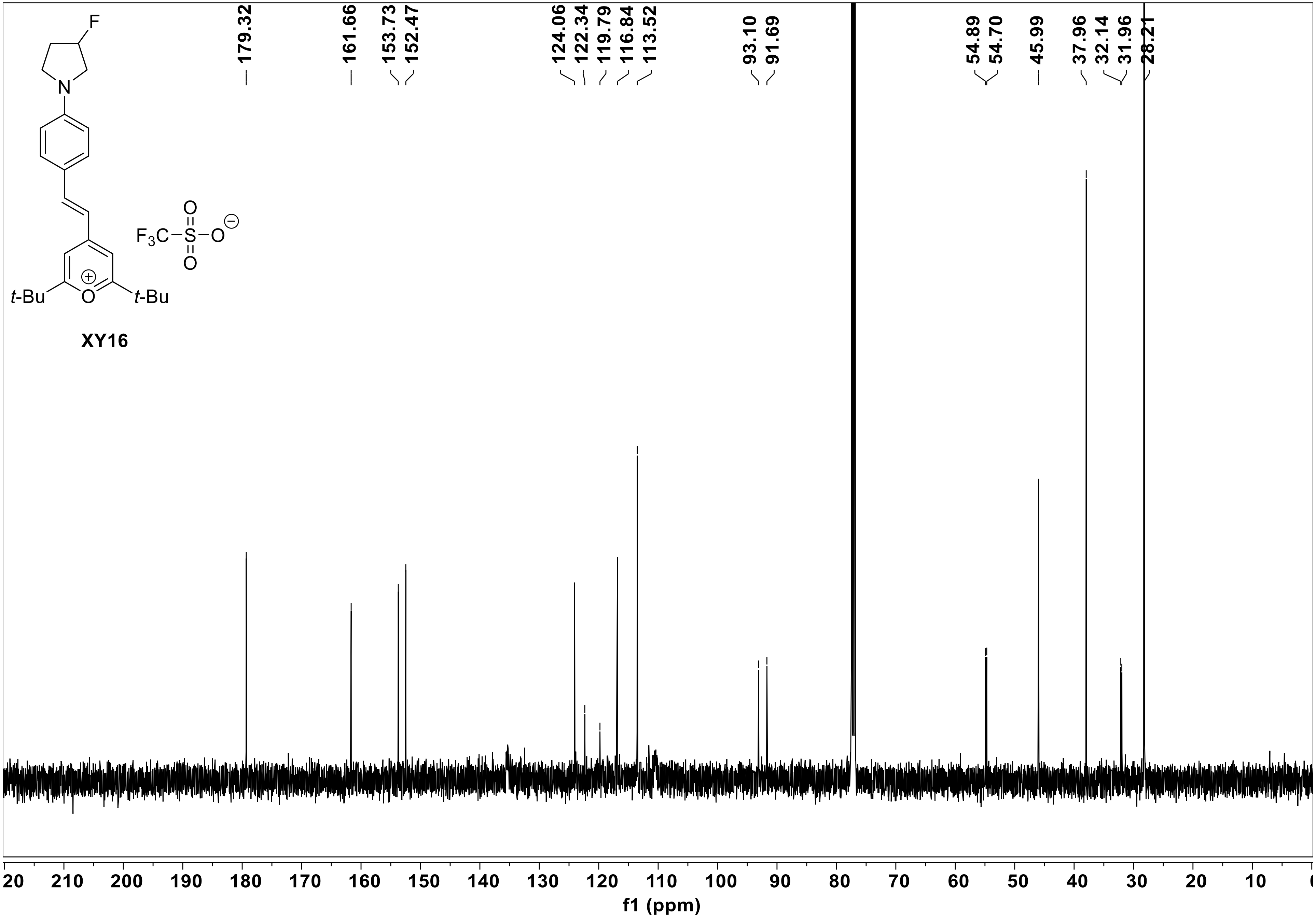

^{19}F NMR Spectrum of XY16 (282 MHz, $CDCl_3$)

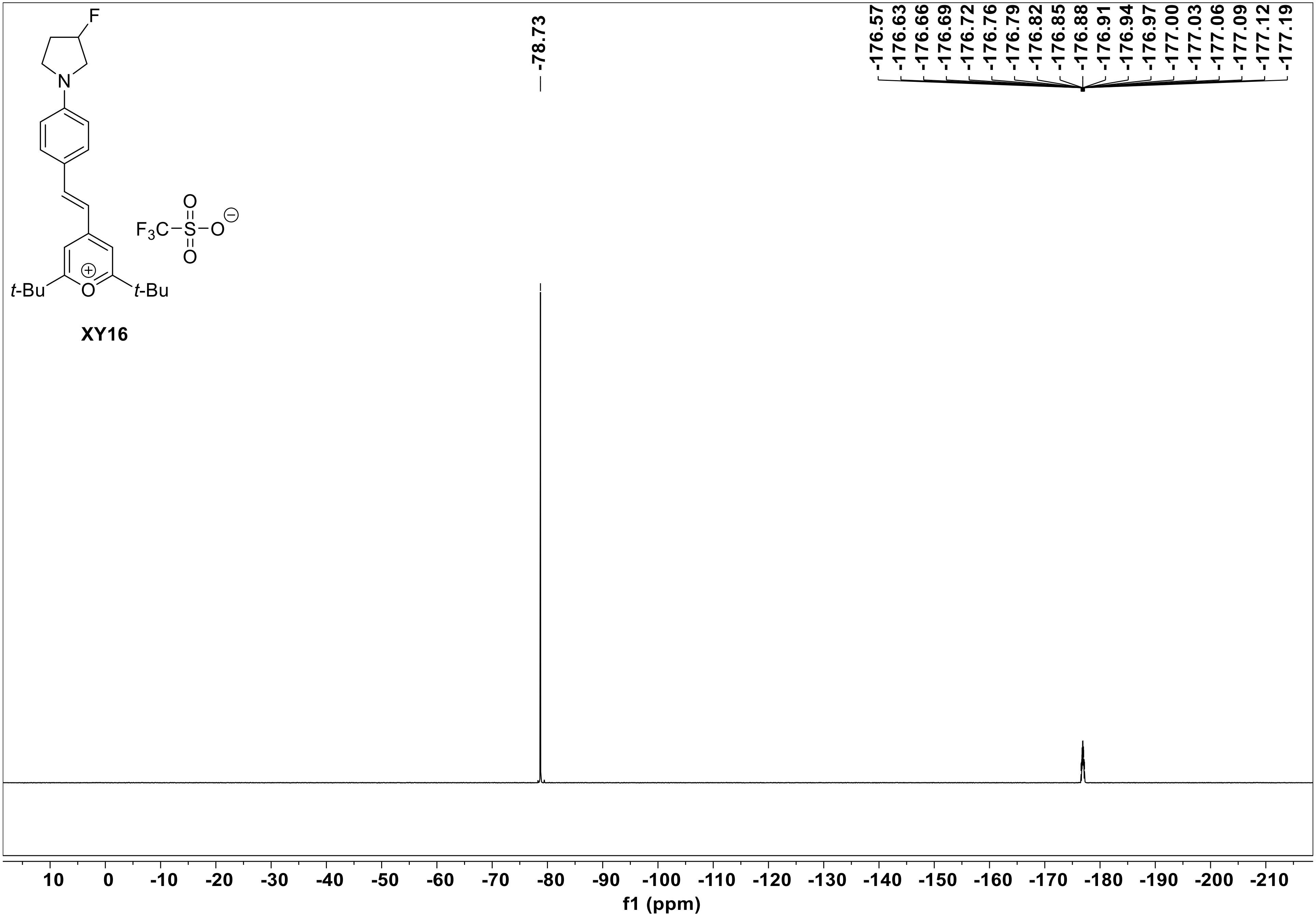

HR-MS Spectra of XY17

Best	ID Source	Name	Formula	Species	m/z	Score	Score (RT)	RT Diff	Diff (ppm)	Score (Lib)	Score (DB)	Score (MFG)
TRUE	MFG		C25 H34 N O2	M+	380.2590	81.73			-0.08			81.73

Species	m/z	Score (iso. abund)	Score (mass)	Score (MFG, MS/MS)	Score (MS)	Score (MFG)	Score (iso. spacing)	Height	Ion Formula
M+	380.259	48.89	100		81.73	81.73	84.59	10017262	C25 H34 N O2

Height (Calc)	Height Sum% (Calc)	Height % (Calc)	m/z (Calc)	Diff (mDa)	Height	Height %	Height Sum %	m/z	Diff (ppm)
11151925.8	75.7	100	380.2584	-0.6	10017262	100	68	380.259	-1.55
3108251.7	21.1	27.9	381.2617	1.2	3997594.2	39.9	27.2	381.2606	3.06
462612.9	3.1	4.1	382.2648	1.2	707934.2	7.1	4.8	382.2636	3.01

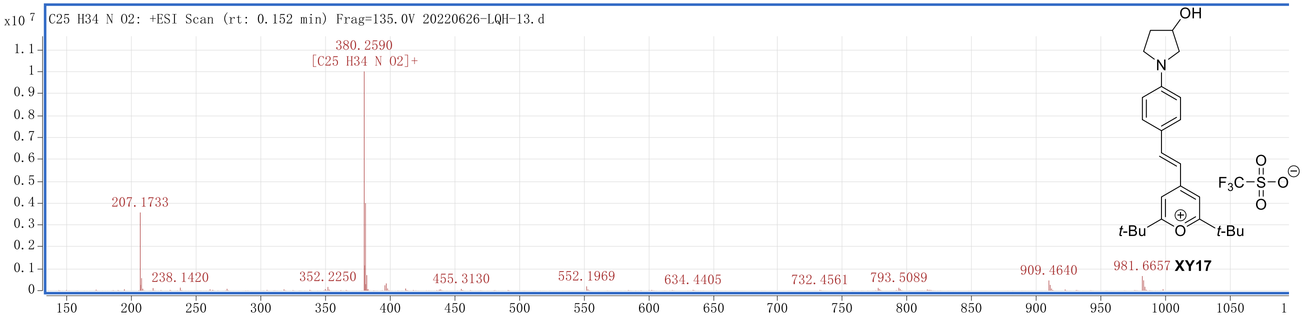

Best	ID Source	Name	Formula	Species	m/z	Score	Score (RT)	RT Diff	Diff (ppm)	Score (Lib)	Score (DB)	Score (MFG)
TRUE	MFG		C F3 O3 S	M-	148.9528	99.21			-1.64			99.21

Species	m/z	Score (iso. abund)	Score (mass)	Score (MFG, MS/MS)	Score (MS)	Score (MFG)	Score (iso. spacing)	Height	Ion Formula
M-	148.9528	98.52	99.26		99.21	99.21	99.92	1828718.4	C F3 O3 S

Height (Calc)	Height Sum% (Calc)	Height % (Calc)	m/z (Calc)	Diff (mDa)	Height	Height %	Height Sum %	m/z	Diff (ppm)
1809417.7	93.4	100	148.9526	-0.3	1828718.4	100	94.4	148.9528	-1.74
35924.3	1.9	2	149.9544	0.1	34307.2	1.9	1.8	149.9543	0.67
92305.2	4.8	5.1	150.9494	-0.1	74621.6	4.1	3.9	150.9495	-0.41

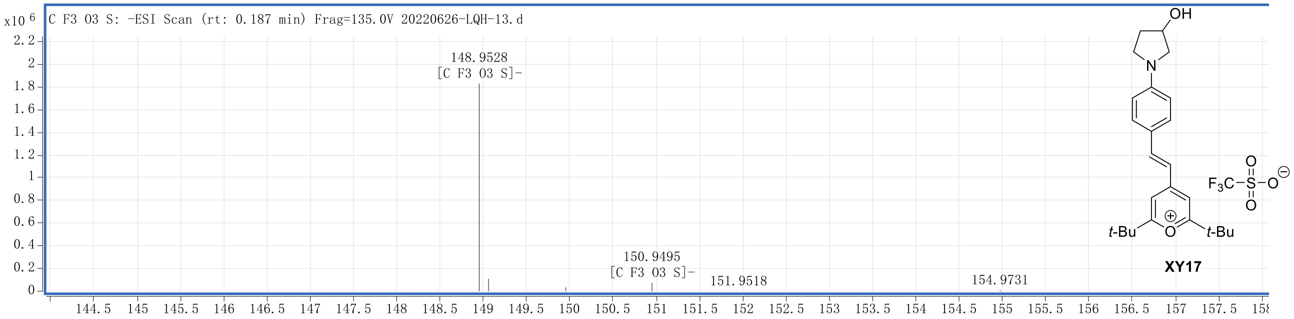

^{1}H NMR Spectrum of XY17 (500 MHz, $CDCl_3$)

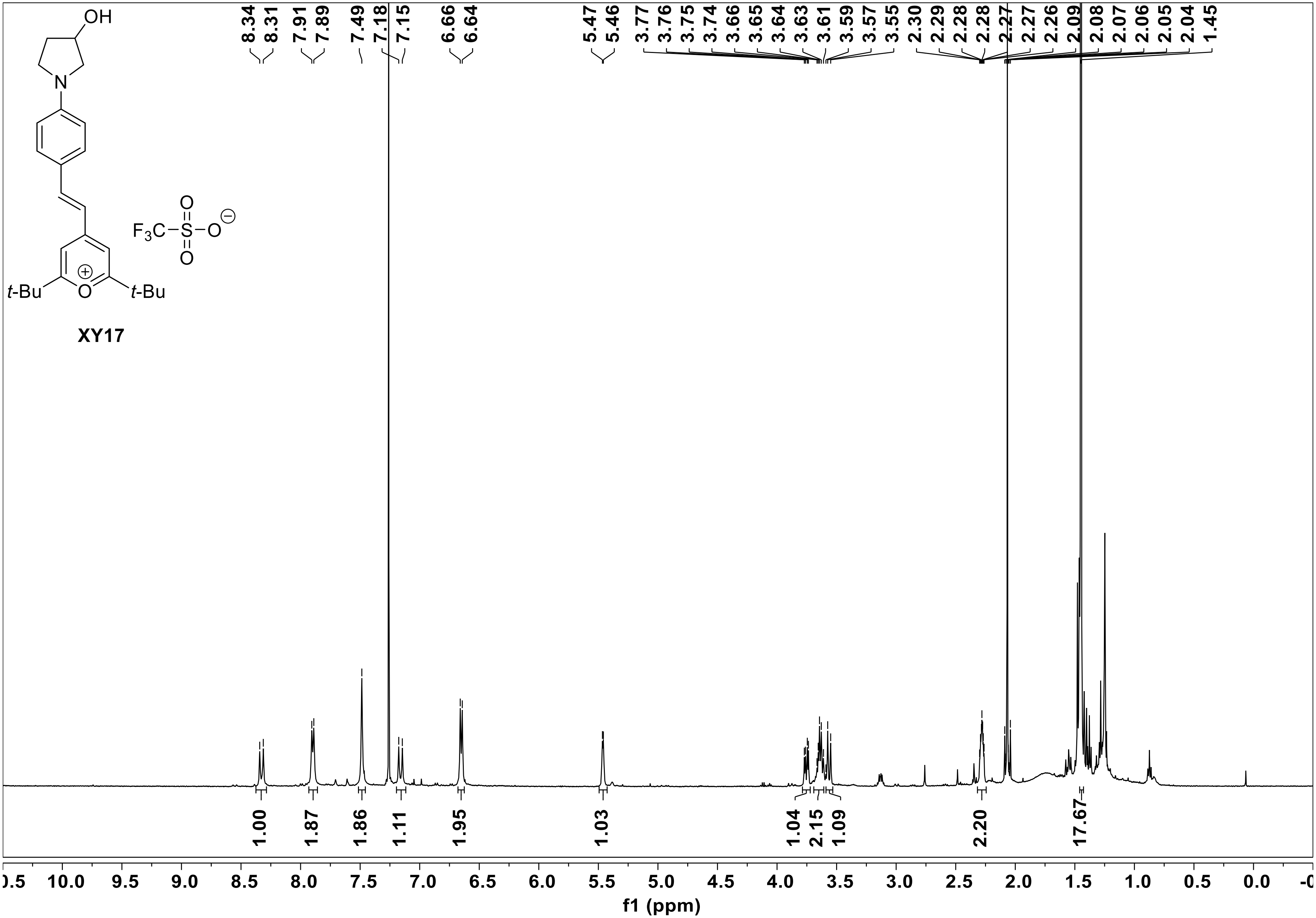

^{13}C NMR Spectrum of XY17 (126 MHz, $CDCl_3$)

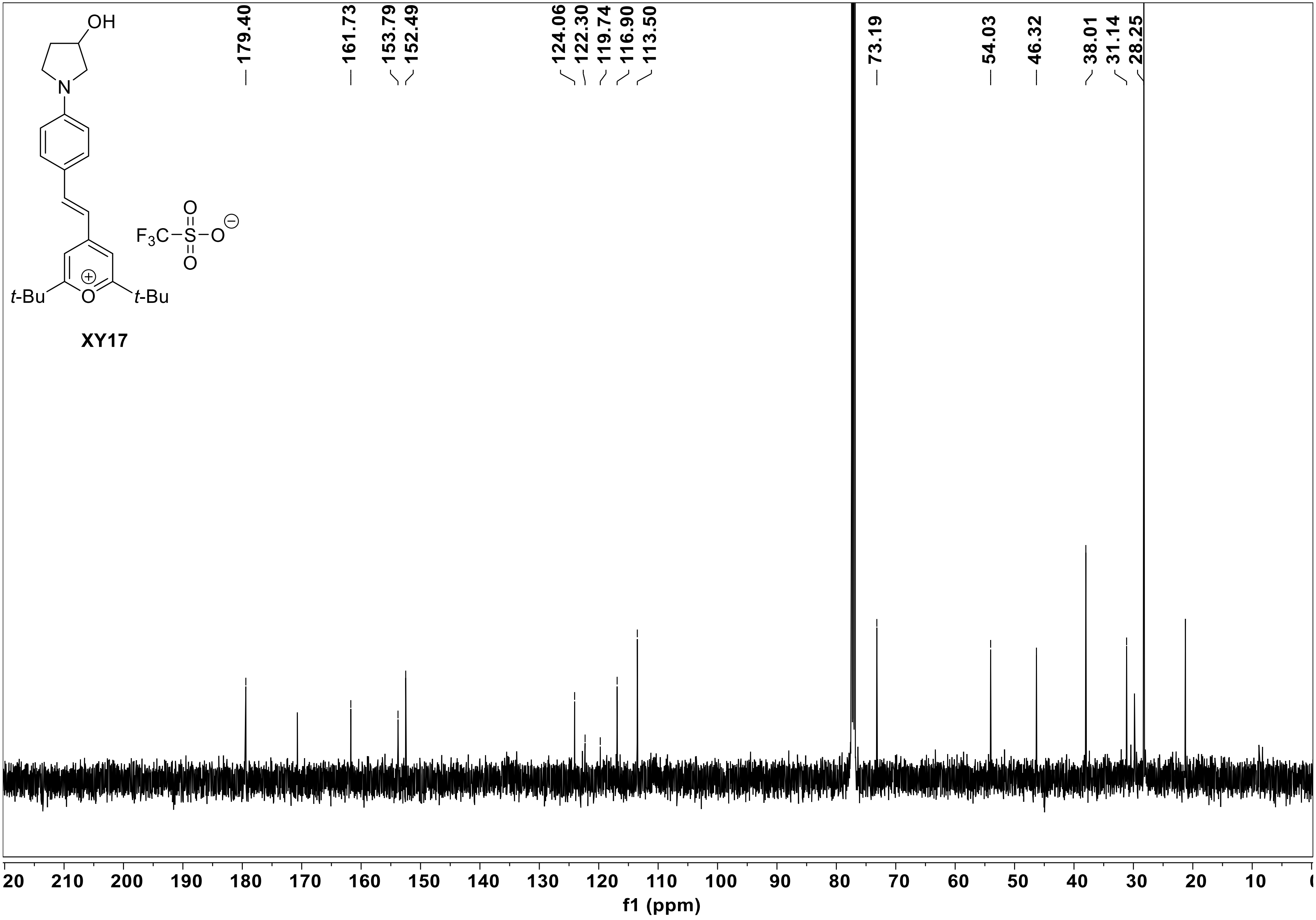

^{19}F NMR Spectrum of XY17 (282 MHz, $CDCl_3$)

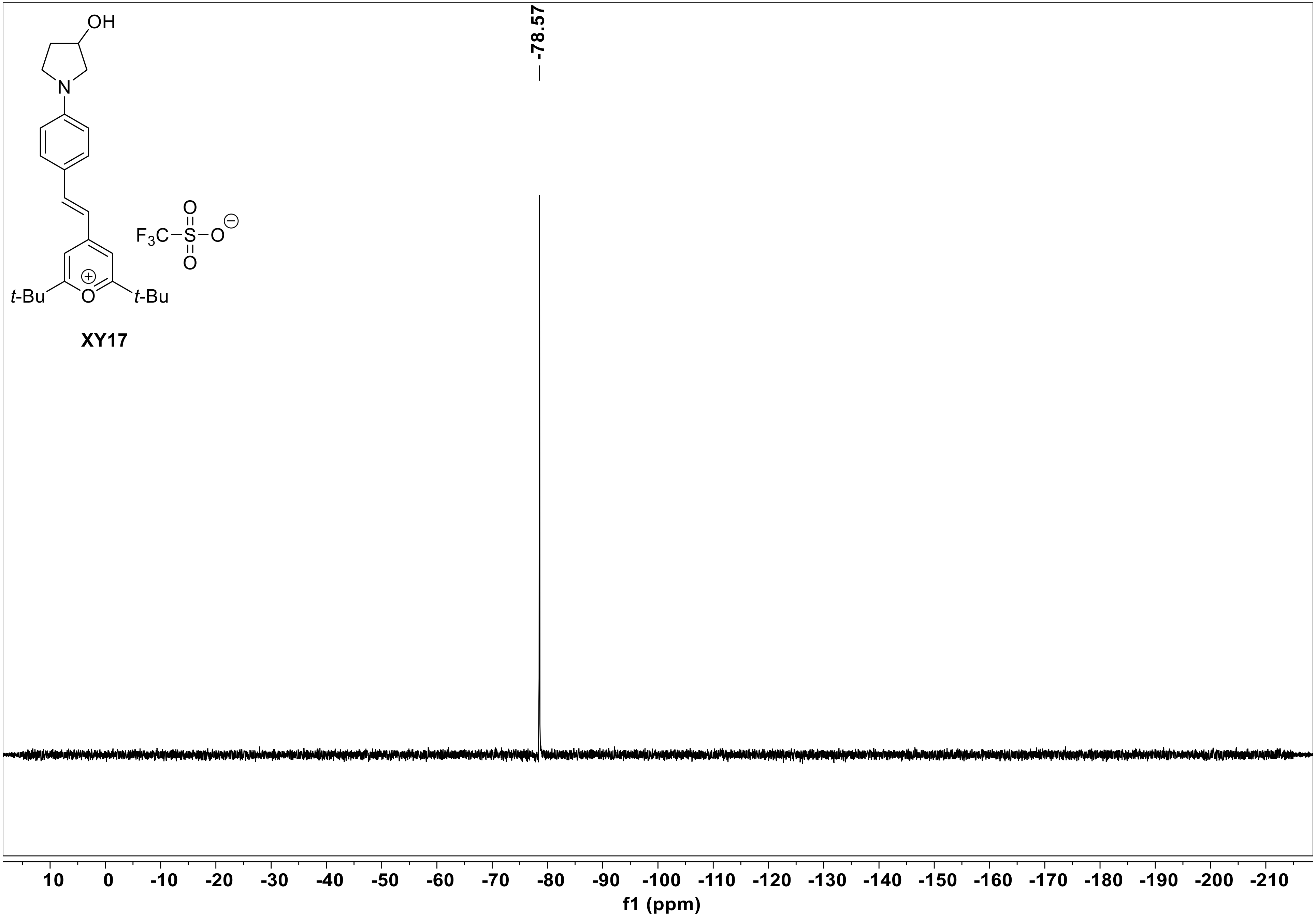

HR-MS Spectra of XY19

Best	ID Source	Name	Formula	Species	m/z	Score	Score (RT)	RT Diff	Diff (ppm)	Score (Lib)	Score (DB)	Score (MFG)
TRUE	MFG		C29 H26 N O	M+	404.2014	77.84			0.52			77.84

Species	m/z	Score (iso. abund)	Score (mass)	Score (MFG, MS/MS)	Score (MS)	Score (MFG)	Score (iso. spacing)	Height	Ion Formula
M+	404.2014	45.35	99.76		77.84	77.84	73.01	10205678	C29 H26 N O

Height (Calc)	Height Sum% (Calc)	Height % (Calc)	m/z (Calc)	Diff (mDa)	Height	Height %	Height Sum %	m/z	Diff (ppm)
11642221.1	72.6	100	404.2009	-0.5	10205678	100	63.6	404.2014	-1.31
3733435.7	23.3	32.1	405.2042	1.2	4708929.5	46.1	29.4	405.203	3.08
602716.9	3.8	5.2	406.2074	2.5	1038870.9	10.2	6.5	406.2049	6.26
65435.8	0.4	0.6	407.2105	3	90331.1	0.9	0.6	407.2076	7.28

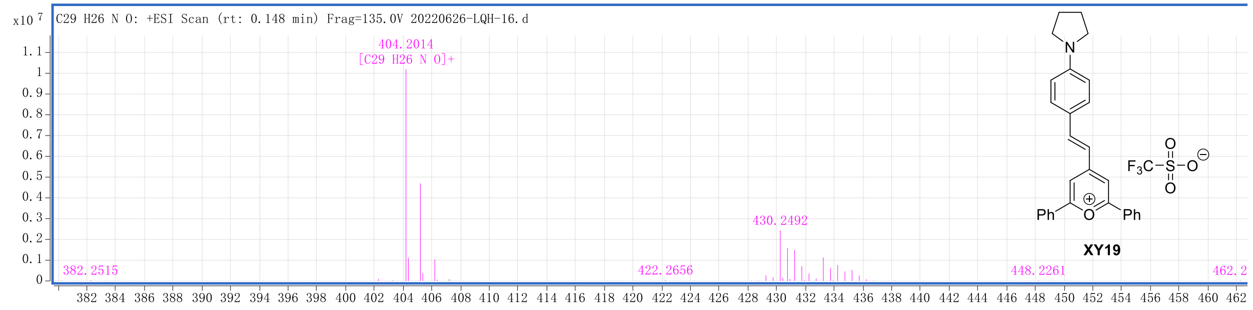

Best	ID Source	Name	Formula	Species	m/z	Score	Score (RT)	RT Diff	Diff (ppm)	Score (Lib)	Score (DB)	Score (MFG)
TRUE	MFG		C F3 O3 S	M-	148.9528	99.4			-1.4			99.4

Species	m/z	Score (iso. abund)	Score (mass)	Score (MFG, MS/MS)	Score (MS)	Score (MFG)	Score (iso. spacing)	Height	Ion Formula
M-	148.9528	98.87	99.46		99.4	99.4	99.91	1681705.8	C F3 O3 S

Height (Calc)	Height Sum% (Calc)	Height % (Calc)	m/z (Calc)	Diff (mDa)	Height	Height %	Height Sum %	m/z	Diff (ppm)
1662344.2	93.4	100	148.9526	-0.2	1681705.8	100	94.5	148.9528	-1.5
33004.3	1.9	2	149.9544	0.1	26164.3	1.6	1.5	149.9543	0.91
84802.4	4.8	5.1	150.9494	0	72280.8	4.3	4.1	150.9494	0.23

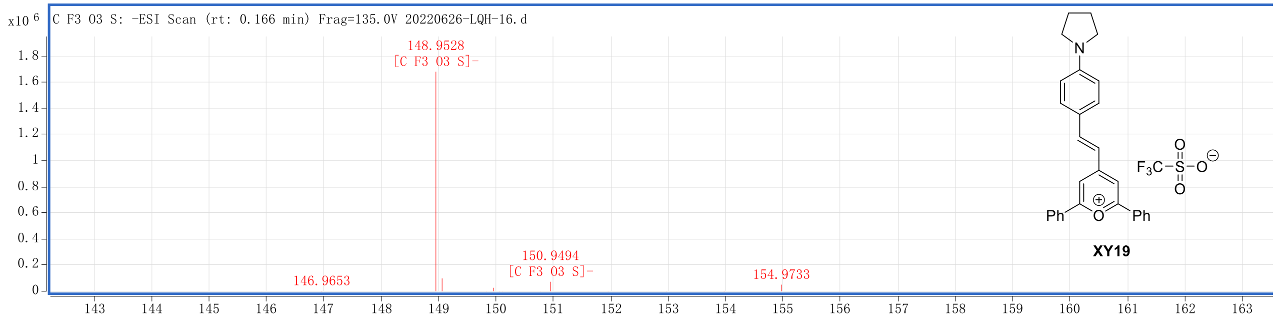

^{1}H NMR Spectrum of XY19 (500 MHz, $CDCl_3$)

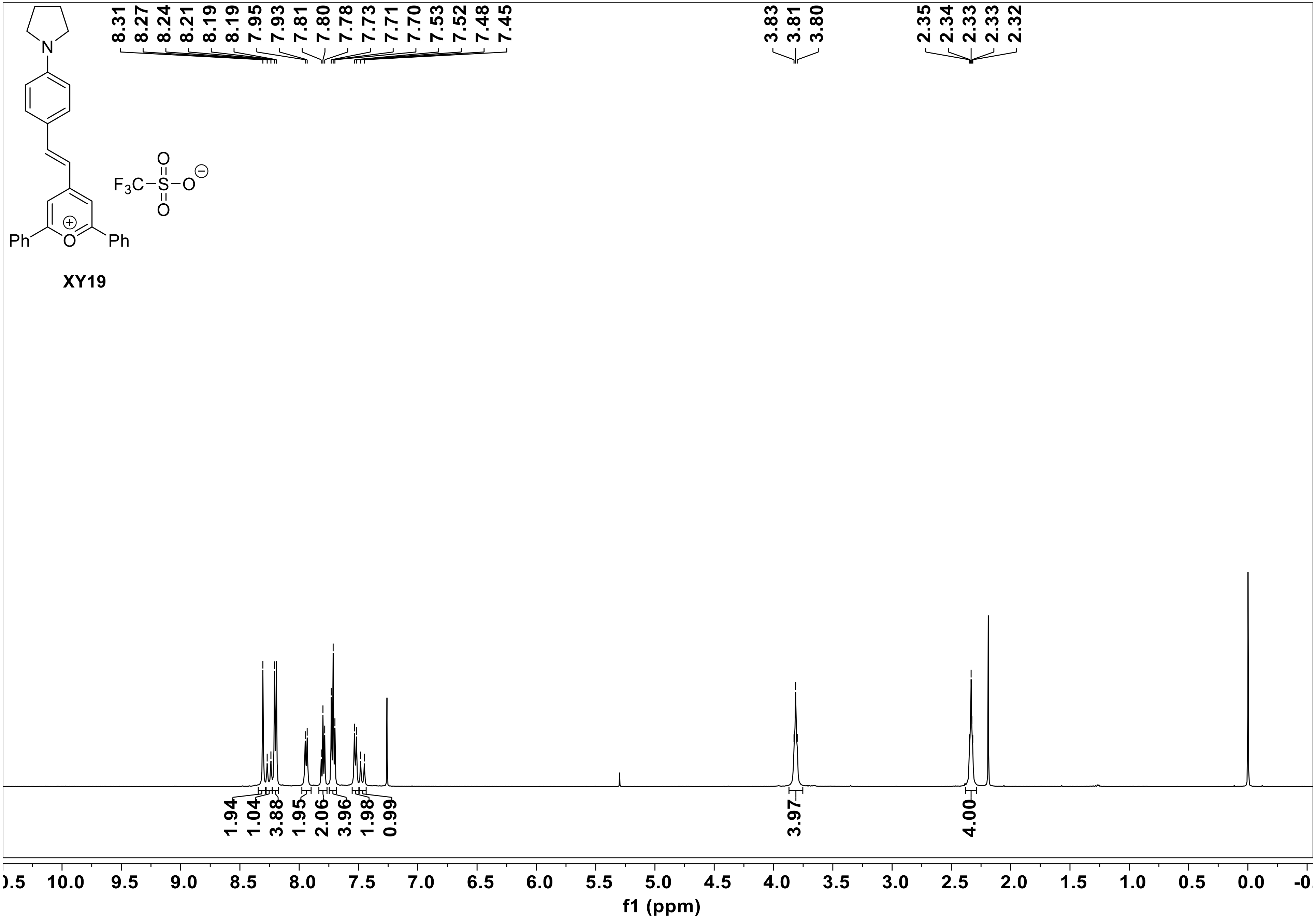

^{13}C NMR Spectrum of XY19 (126MHz, $CDCl_3$+TFA)

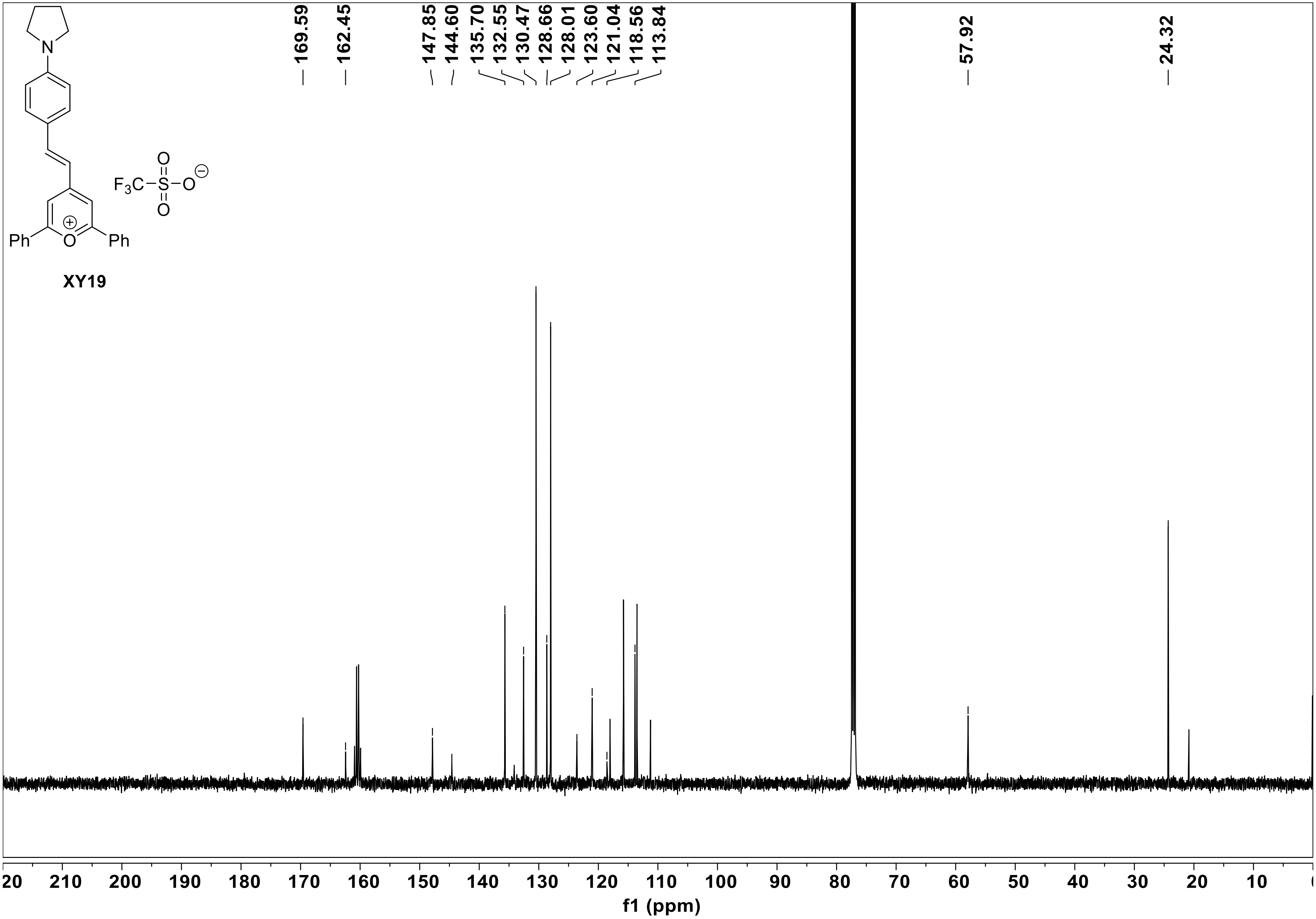

^{19}F NMR Spectrum of XY19 (282 MHz, $CDCl_3$+TFA)

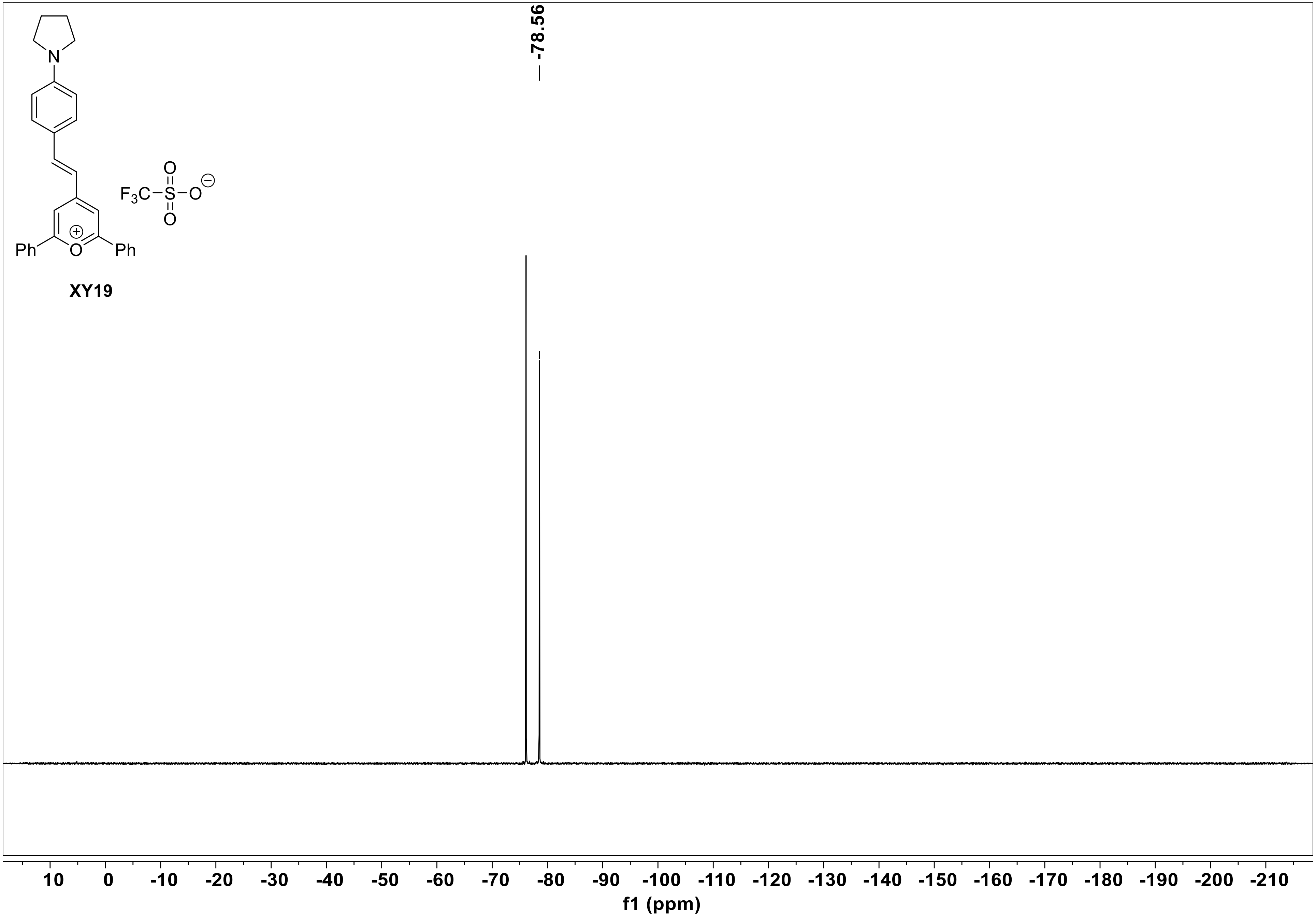

HR-MS Spectrum of XY20

Best	ID Source	Name	Formula	Species	m/z	Score	Score (RT)	RT Diff	Diff (ppm)	Score (Lib)	Score (DB)	Score (MFG)
TRUE	MFG		C26 H35 N	(M+H)+	362.2841	99.14			0.7			99.14

Species	m/z	Score (iso. abund)	Score (mass)	Score (MFG, MS/MS)	Score (MS)	Score (MFG)	Score (iso. spacing)	Height	Ion Formula
(M+H)+	362.2841	99.58	99.61		99.14	99.14	97.67	2224502	C26 H36 N

Height (Calc)	Height Sum% (Calc)	Height % (Calc)	m/z (Calc)	Diff (mDa)	Height	Height %	Height Sum %	m/z	Diff (ppm)
2237186.7	75.2	100	362.2842	0.1	2224502	100	74.8	362.2841	0.26
646553	21.7	28.9	363.2875	0.6	647749	29.1	21.8	363.287	1.54
90010.1	3	4	364.2909	1.8	101498.8	4.6	3.4	364.2891	4.83

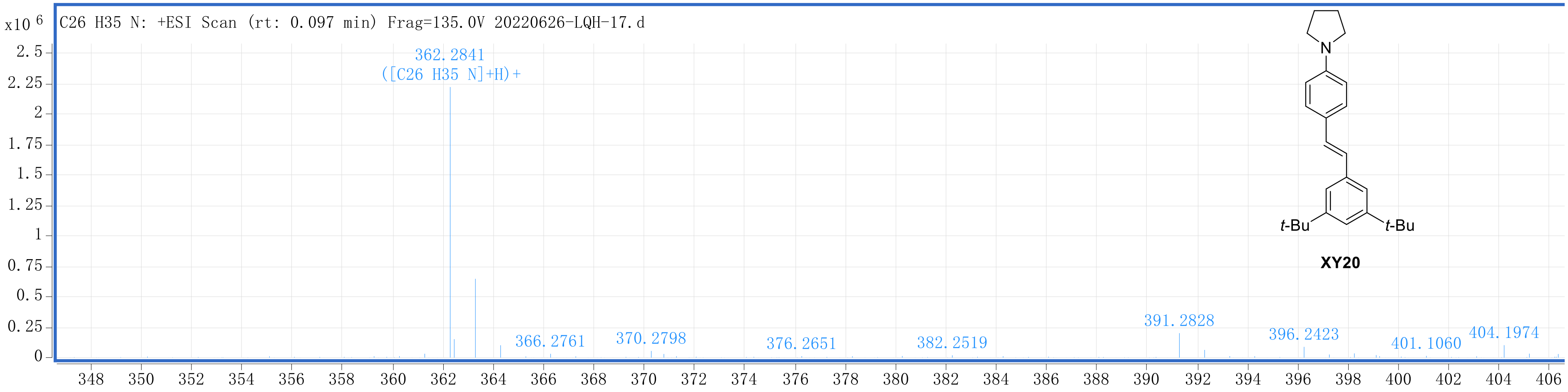

^{1}H NMR Spectrum of XY20 (300 MHz, $CDCl_3$)

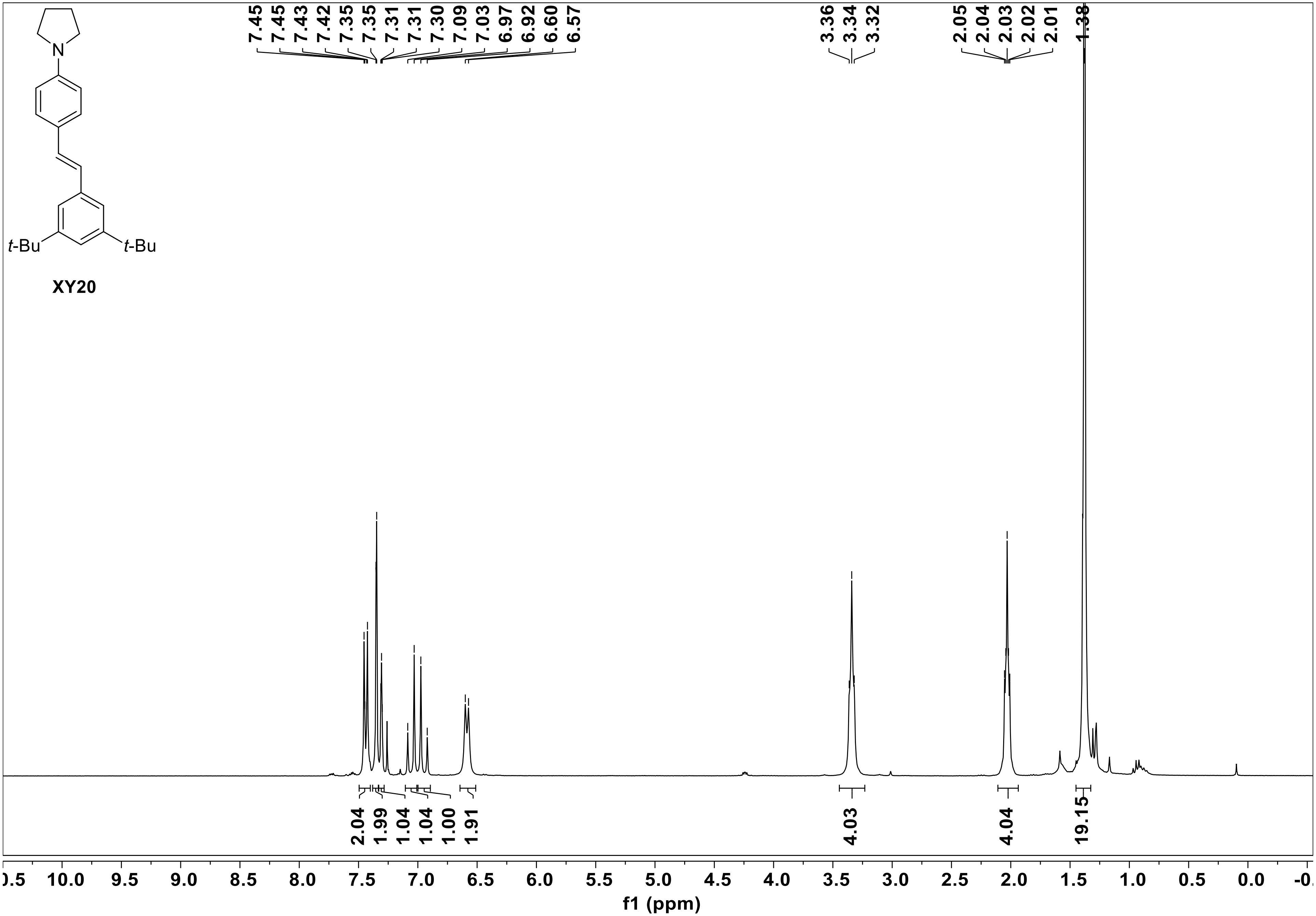

^{13}C NMR Spectrum of XY20 (75 MHz, $CDCl_3$)

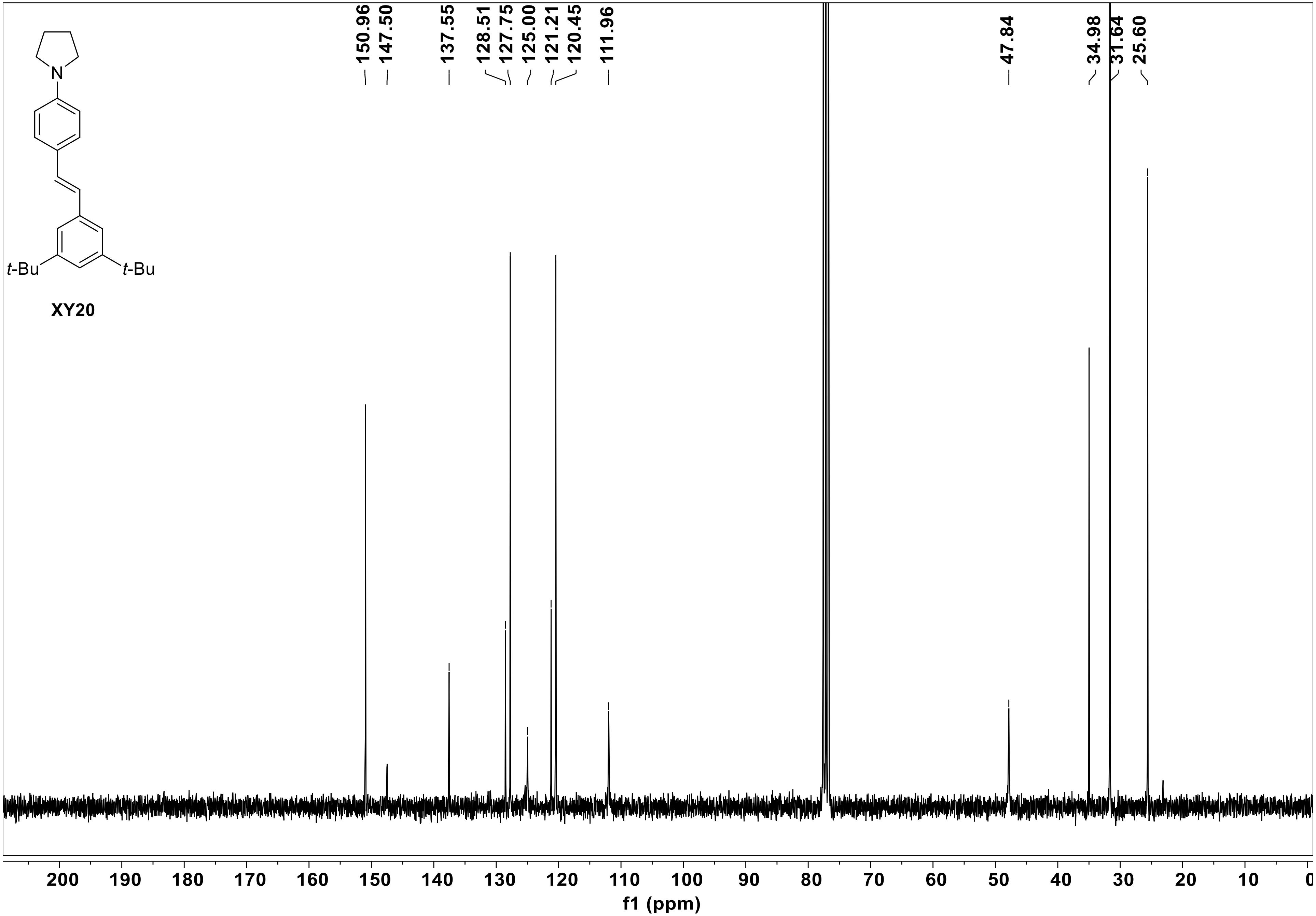

Supplement: Supplementary file 1 [file molecules-27-04450-s001.zip › molecules-1785566-supplementary.pdf]
